# High throughput sequencing of small RNAs reveals dynamic microRNAs expression of lipid metabolism during *Camellia oleifera* and *C. meiocarpa* seed natural drying

**DOI:** 10.1186/s12864-017-3923-z

**Published:** 2017-07-20

**Authors:** Jin-Ling Feng, Zhi-Jian Yang, Shi-Pin Chen, Yousry A. El-Kassaby, Hui Chen

**Affiliations:** 1 College of Forestry, Fujian Agriculture and Forestry University, Fuzhou, 350002 China; 2 Department of Forest and Conservation Sciences, Faculty of Forestry, University of British Columbia, Forest Sciences Centre, 2424 Main Mall, Vancouver, BC V6T 1Z4 Canada

**Keywords:** miRNAs, Lipid metabolism, Natural drying, *Camellia meiocarpa*, *Camellia oleifera*

## Abstract

**Background:**

Camellia species are ancient oilseed plants with a history of cultivation over two thousand years. Prior to oil extraction, natural seed drying is often practiced, a process affecting fatty acid quality and quantity. MicroRNAs (miRNA) of lipid metabolism associated with camellia seed natural drying are unexplored. To obtain insight into the function of miRNAs in lipid metabolism during natural drying, Illumina sequencing of *C. oleifera* and *C. meiocarpa* small-RNA was conducted.

**Results:**

A total of 274 candidate miRNAs were identified and 3733 target unigenes were annotated by performing a BLASTX. Through integrated GO and KEGG function annotation, 23 miRNA regulating 131 target genes were identified as lipid metabolism, regulating fatty acid biosynthesis, accumulation and catabolism. We observed one, two, and four miRNAs of lipid metabolism which were specially expressed in *C. Meiocarpa*, *C. oleifera*, and the two species collectively, respectively. At 30% moisture contents, *C. meiocarpa* and *C. oleifer* produced nine and eight significant differentially expressed miRNAs, respectively, with high fatty acid synthesis and accumulation activities. Across the two species, 12 significant differentially expressed miRNAs were identified at the 50% moisture content.

**Conclusions:**

Sequencing of small-RNA revealed the presence of 23 miRNAs regulating lipid metabolism in camellia seed during natural drying and permitted comparative miRNA profiles between *C. Meiocarpa* and *C. oleifera.* Furthermore, this study successfully identified the best drying environment at which the quantity and quality of lipid in camellia seed are at its maximum.

**Electronic supplementary material:**

The online version of this article (doi:10.1186/s12864-017-3923-z) contains supplementary material, which is available to authorized users.

## Background

*C. oleifera* and *C. meiocarpa* belong to the camellia family, Theaceae, and are known for their high quality oilseed that is dubbed as the Eastern olive oil [1, 2]. Some of the camellia species are ancient oilseed plants with history of cultivation for over two thousand years [3]. Camellia oil is known for its edible and medicinal uses with an oleic acid content reaching above 80%, a high content of monounsaturated lipid, and the lowest level of saturated fats [4]. Camellia oil aids in cholesterol reduction, resistance to stress, oxidation reduction, reduced inflammation, and improved human immunity [5]. The drying management of camellia seed prior to oil extraction is a fundamental factor affecting its fatty acid quality and quantity [6–8].

MicroRNAs (miRNAs) are small regulatory molecules that have been shown to be involved in a wide range of biological pathways by modulating expression of specific mRNAs [9]. Sequencing small RNA libraries demonstrated the role of miRNAs in lipid metabolism in plants (safflower [10]; *Camelina sativa* [11]; soybean [12]; wheat [13]; *Jatropha* [14]; *Arabidopsis* [15]), animals [16, 17], and insects [18]). Different oil crops were differentially expressed with different seed oil content and composition. There were 28 miRNAs regulated lipid metabolism from soybean [12], 30 miRNAs from *Camelina sativa* [11], and 13 miRNAs differentially expressed from two safflower genotypes that have difference to regulate oleic acid accumulation [10]. So there may be different miRNA expression between *C. Meiocarpa* and *C. oleifera.*

Recent research uncovered miRNAs regulation of lipid metabolism, which related to diverse pathways, such as fatty acid synthesis, accumulation, and catabolism. For example, miR159b, miR5026, and miR2911 were identified to encode ∆12-desaturase (FAD2) [10, 15, 19, 20], miR408a and miR159b for Fatty acid elongase (FAE1) [11, 15, 44], which related to fatty acid synthesis. Additionally, miR319a, miR001 and miR007 were identified to encode 1-acylglycerol-3-phosphocholine acyltransferase (LPCAT) [11, 14], which involved in fatty acid accumulation and miR166a, miR2910, miR824, miR414, and miR5206 were identified to encode fatty acid oxidase [11, 13, 14, 21], which regulate fatty acid catabolism. As above, the same fatty acid protein can be regulated by different miRNA, and the same miRNA can encode different fatty acid protein. In *C. Meiocarpa* and *C. oleifera*, the presence of different miRNAs expression pattern at different environments and, in particular, in dry seed suggest that miRNAs may play critical roles in lipid metabolism during natural seed drying [22–27].

In order to explore the potential role of miRNAs in lipid metabolism during *C. Meiocarpa* and *C. oleifera* natural drying, miRNA expression profiles of seed samples at different moisture content (10, 20, 30, 40, and 50%) were investigated using high throughput next generation small RNA sequencing technology, so the differentially expressed miRNAs are unraveled to help understand their involvement in lipid metabolism.

## Methods

### Plant material

In 2012, mature fruits of *C. meiocarpa* and *C. oleifera* were collected from the four cardinal directions of 3 superior trees’ crowns per species. The trees are growing at the Minhou Tongkou State Forest Farm (26°05′ N, 119°17′ E), Fujian Province, China. The collected fruits were placed in a ventilated room until they naturally cracked and seeds were extracted by manual shell cutting. The seed moisture content at the time of extraction was closed to 50%. Seeds were naturally dried and their moisture content was determined daily. Over time, seed samples were collected at moisture content of 50, 40, 30, 20, and 10% and were sequentially identified as S01 to S05 and S06 and S10 for *C*. *meiocarpa* and *C. oleifera*, respectively, and sampled three times, then were flash frozen in liquid nitrogen and stored at −80 °C until RNA extraction.

### Oil content analysis

To obtain the camellia seed oil content, the Soxhlet method was used by a fatty acid instrument (SZF-06A, Shanghai Hongji Instrument Equipment company) [28]. Camellia seeds at different moisture content were cut into thin slices, dried by silica gel and liquid nitrogen, milled into a powder by a pulverizer (FW100, Tianjin Taisite Instrument company). About 2 g of powder were weighed out (M1), packed in a folded filter paper bag and bound with a skim cotton thread, placed into the Soxhlet extraction thimble with 15 mL of petroleum ether for 1 h. Soxhlet extraction bottle were weight (M2) into which extraction thimble was placed, extracted for 5 h using 70 ml petroleum ether (75 °C). After extraction, the solvent was evaporated in Soxhlet extraction bottle and dried at 75 °C drying oven. Then Soxhlet extraction bottle was weighed again (M3).

$$ \mathrm{Seed}\ \mathrm{oil}\ \mathrm{content}=\left(\mathrm{M}3-\mathrm{M}2\right)/\mathrm{M}1\times 100\% $$

All experiments were carried out at least in triplicate and data were analyzed using the SPSS statistics 17.0 software.

### RNA isolation

Total RNA was extracted from the seed samples of the two camellia species using RNA kit (RNA simply total RNA kit, Tiangen, Beijing, China) according to the manufacturer’s instructions. The purity and quality of the RNA were determined by assessing the absorbance ratio OD260/280 using NanoDrop ND1000 Spectrophotometer (NanoDrop, Wilmington, DE). The RNA was quantified with a Qubit 2.0 Fluoremeter (Invitrogen Corporation, Carlsbad, CA, USA) in accordance with the manufacturer’s instructions. The integrity of the RNA samples was verified using an Agilent 2100 Bioanalyzer (Agilent, Santa Clara, CA, USA).

### Small RNA library construction and sequencing

Equal amount of total RNA from the three samples at each moisture content of the two camellia species, was mixed to construct a transcriptome library using an Illumina TruSeq RNA Sample PrepKit following the manufacturer’s instructions. Small RNAs of 18–30 nt in length were separated and purified by denatureing polyacrylamide gel electrophoresis. After dephosphorylation and ligation of a pair of Solexa adaptors to their 5′ and 3′ ends, the products were reverse-transcribed and amplified by RT-PCR and gel purification. After the library was constructed, the Qubit 2.0 Fluorometer (Invitrogen Corporation, Carlsbad, CA, USA) were used to calculate the molar concentration and confirm the insert size. The cDNA libraries were sequenced using the Illumina HiSeq2500 Genome analyzer (Illumina Inc., San Diego, CA, USA).

### miRNA-Seq data analysis

After sequencing, the raw reads (FASTQ files) were processed into clean reads, then the adaptor sequences, poy(A) tails and the inserted tag were removed, followed by filtering the low-quantity reads (ambiguous bases ‘N’ ≥ 10% and more than 20% with Quality Score < 30 bases), the clean 18–30 nt sRNAs were mapped to GenBank (http://www.ncbi.nlm.nih.gov/), Rfam (version 10.1) database (http://rfam.sanger.ac.uk), tRNAdb [29], SILVA rRNA [30] and Repbase (http://www.girinst.org/), and rRNA, tRNA, snRNA, and snoRNA were discarded from the sRNA reads using bowtie2 software with perfect matches (0 mismatches) used for further analysis [31].

The unannotated sequences were then analyzed by miRDeep-P software package to predict miRNAs, which was developed by modifying miRDeep2 [32]. All mature sequences, star sequences and precursor sequences cored by miRDeep2 were retained and regarded as putative miRNAs and used for further analysis to identify known and novel miRNAs [33, 34]. Known miRNAs were annotated by identifying their homologous imRNAs in miRBase database (http://www.mirbase.org/) using the following criteria: 1) seed region, nucleotides 2–7 must be identical; and 2) the remainder of the sequence alignment must contain no more than two mismatches [35, 36]. The putative miRNAs produced by miRDeep2 analysis were regarded as conservative miRNAs when it met the above criteria. Those miRNAs produced by miRDeep2 analysis that did not meet the above criteria were considered as novel miRNAs. In order to predict novel miRNAs with high confidence, only those with a miRDeep-P score higher than ≥0 were retained as true novel miRNAs [34, 37].

### Screening of differentially expressed miRNAs

Differentially expressed miRNAs were identified using the TPM [38] and IDEG6 software [39]. TPM (Tags Per Million reads) is a standardized method for calculating miRNA expression levels. TPM values were calculated using the following equation: TPM = number of mapped miRNA reads/number of clean sample reads × 10^6^. In order to calculate the levels of differential expressed miRNAs, normally the value was set as 0.01 by default when the sequencing read is 0 (no reads) [40]. We calibrated miRNA expression levels using multiple hypothesis tests with a false discovery rate (FDR) ≤0.01, performed generalized chi-square tests for differential miRNA expression using the IDEG6 software (http://telethon.bio.unipd.it/bioinfo/IDEG6/), and screened the miRNAs for those with *P*-values less than 0.01 or TPM ratios between samples that were greater than 1 (fold change ≥1) or FDR ≤0.01. The miRNAs that met these criteria were identified as being differentially expressed [41].

### miRNA target prediction

The putative target sites of miRNAs were identified by aligning mature miRNA sequences with a draft genome sequence using TargetFinder, (http://carringtonlab.org/resources/targetfinder). miRNA targets were computationally predicted essentially as described [38, 42, 43]. Briefly, potential targets from FASTA searches were scored using a position-dependent, mispair penalty system. Penalties were assessed for mismatches, bulges, and gaps (+1 per position) and G:U pairs (+0.5 per position). Penalties were doubled if the mismatch, bulge, gap, or G:U pair occurred at positions 2 to 13 relative to the 59 end of the miRNAs. Only one single-nucleotide bulge or single-nucleotide gap was allowed, and targets with penalty scores of four or less were considered to be putative miRNA targets [42, 44].

Functional annotation of predicted target genes was assigned using Nr (non-redundant protein database, NCBI), Nt (non-redundant nucleotide database, NCBI), Swiss-Prot, GO (gene ontology, http://www.geneontology.org/) and COG (clusters of orthologous groups) databases. BLASTX was employed to identify related sequences in the protein databases based on an Evalue of less than 10^−5^ [45]. Gene ontology (GO) and Kyoto encyclopedia of genes and genomes (KEGG) enrichment analysis were performed with package GO stats (http://www.geneontology.org/) of *P* value <0.05 was set as the cut-off to select out significantly enriched terms [46].

### Quantitative real-time PCR analysis of miRNAs

qRT-PCR was used to validate the miRNAs identified using deep sequencing and to analyze their expression patterns. Total RNA of *C. meiocarpa* and *C. oleifera* seeds samples, was extracted using TRUzol Universal Reagent according to the manufacturer’s protocol. They were then reverse-transcribed into cDNA using the microRNA cDNA First Strand cDNA Synthesis Kit (CWBIO, Beijing, China) according to the manufacturer’s instructions. The cDNA was quantified by microRNA Real-Time PCR Assay Kit (CWBIO, Beijing, China) using a 20 μL reaction mixture, which consisted of 1 μL of diluted cDNA, 0.25 μM forward and reverse primer, and 10 μL of 2 × SYBR Green PCR Master Mix (CWBIO, Beijing, China). All reactions were performed under the following conditions: 95 °C for 5 min, 40 cycles of 95 °C for 15 S, 62 °C for 45 S. Melting curve analysis was performed to verify specific amplification (from 72 to cycles at 60 °C for 15 S). Each sample was processed in triplicate, and 5.8S rRNA was used as an internal control [47, 48]. The equation ratio 2 ^-∆∆CT^ was applied to calculate the relative expression level of miRNAs. The qRT-PCR primers are listed in file (Additional file 1: Table S1) and Ct value of 5.8S (Additional file 1: Table S2).

## Results

### Oil content of two camellia species during natural seed drying

Camellia oil content were determined by Soxhlet extraction method during natural seed drying (Table 1). The two species showed increased in oil content at 50 to 30% moisture contents followed by a slow decline at 30 to 10% moisture content, with 30% moisture content producing the highest oil content. Oil content accumulation was largest when the seed moisture content dropped from 50 to 40% and 40 to 30% for *C. meiocarpa* and *C. oleifera*, respectively. At 30% moisture content, *C. meiocarpa* and *C. oleifera* showed increase in their seed oil content of 8.80 and 10.23%, respectively, indicating that the effect of appropriate seed natural drying on oil accumulation can be great. While the percentages of oil content increase seem different between the two species, the relative increase amounted to 4.40%, indicating that natural drying can promote oil accumulation in both camellia specie.

**Table 1:** Seed oil content during camellia seed natural drying

Moisture content (%)	Seed oil content (%)	
	*C. meiocarpa*	*C. oleifera*
50	33.41 ± 0.001d	37.66 ± 0.117e
40	39.91 ± 0.002b	38.72 ± 0.116d
30	42.21 ± 0.001a	47.89 ± 0.001a
20	40.35 ± 0.002b	44.25 ± 0.001b
10	37.83 ± 0.001c	42.09 ± 0.001c

different letters indicate significant at *P* < 0.05

### Sequence analysis of small RNAs

To obtain a comprehensive profile of the sRNAs involved in natural drying, camellia seed from both species were subjected to Solexa high-throughput sequencing, with 5 libraries for each species corresponding to the sampled moisture contents. Average total of 30,641,435 and 31,012,304 reads were generated from *C. meiocarpa* and *C. oleifera*, respectively (Table 2). After filtering the low quality reads, such as 3′ insert null, poly(A), length < 18 nt or length > 30 nt, and other artifacts, the majority of the small RNAs were 21 to 24 nt in length. A total of 24,070,601 and 21,653,584 clean reads of 18–30 nt were obtained for *C. meiocarpa* and *C. oleifera*, respectively (Table 2). GC content of clean reads were more than 47.75 and 48.99 and the Q30 (meaning 1 error in 1000 reads) of clean reads were more than 85.29 and 85.25% for *C. meiocarpa* and *C. oleifera*, respectively (Table 2). Through quality control, each sample of clean data were greater than 19.40 M, indicating the high sRNA quality (Tables 2, 3 and 4).

**Table 2:** Statistics relating to *C. meiocarpa* and *C. oleifera* sRNA sequences produced by Solexa sequencing

Species	Samples	Raw reads	<18 nt reads	>30 nt reads	Clean reads	GC(%)	Q20(%)	CycleQ20(%)	Q30(%)
*C. meiocarpa*	S01	29,906,668	1,658,148	7,025,688	20,417,236	49.32	93.1	100	86.98
	S02	32,844,767	1,179,743	3,844,931	27,795,553	47.75	91.9	100	86.17
	S03	28,888,019	1,676,660	3,899,605	23,290,661	48.54	91.42	100	85.47
	S04	28,599,822	1,902,807	3,697,488	2,2978,269	48.32	91.22	100	85.29
	S05	32,967,901	2,333,282	4,738,442	25,871,287	48.67	91.24	100	85.36
	Average	30,641,435	1,750,128	4,641,231	24,070,601	48.52	91.78	100	85.86
*C. oleifera*	S06	34,169,238	4,386,045	4,606,664	25,151,946	49.13	91.23	100	85.25
	S07	31,865,014	1,751,974	9,877,436	19,405,898	49.28	93.21	100	87.02
	S08	30,501,297	2,833,009	4,644,107	23,001,131	48.99	91.35	100	85.43
	S09	31,087,840	3,519,595	5,720,360	21,016,629	49.7	93.05	100	86.74
	S10	27,438,132	3,369,136	3,659,237	19,692,318	49.55	93.09	100	86.86
	Average	31,012,304	3,171,952	5,701,561	21,653,584	49.33	92.39	100	86.26

**Table 3:** Distribution of small RNAs among different categories during natural drying in *C. meiocarp*a

Category	S01	S02	S03	S04	S05	Average
rRNA	2,538,153	2,904,882	3,126,948	3,295,085	4,259,745	3,224,963
snRNA	3532	6061	3311	5799	5000	4741
snoRNA	161	194	118	141	140	151
tRNA	337,182	247,988	383,889	368,857	485,157	364,615
Repbase	9927	9090	15,485	12,733	14,597	12,366
Unannotated	17,528,281	24,627,338	19,760,910	19,295,654	21,106,648	20,463,766
Total	20,417,236	27,795,553	23,290,661	22,978,269	25,871,287	24,070,601

**Table 4:** Distribution of small RNAs among different categories during natural drying in *C. oleifera*

Category	S06	S07	S08	S09	S10	Average
rRNA	4,061,785	3,880,373	4,384,408	5,417,932	4,269,148	4,402,729
snRNA	5381	4753	6280	6407	3761	5316
snoRNA	376	255	652	351	1224	572
tRNA	368,965	437,180	641,000	662,085	677,875	557,421
Repbase	18,113	11,547	11,476	14,753	13,396	13,857
Unannotated	20,697,326	15,071,790	17,957,315	14,915,101	14,726,914	16,673,689
Total	25,151,946	19,405,898	23,001,131	21,016,629	19,692,318	21,653,584

After searching against GenBank, Rfam, tRNAdb, Silva, and Repbase database for small RNA sequences by bowtie2 software, rRNA (3,224,963), snRNA (4741), snoRNA (151), tRNA (364,615), Repbase-associated sRNAs (12,366) were annotated and removed, and other unannotated RNAs (20,463,766) were obtained for *C .meiocarpa* on average (Table 3). The same process was conducted for *C. oleifera* and rRNA (4,402,729), snRNA (5316), snoRNA (572), tRNA (557,421), Repbase-associated sRNAs (13,857) were annotated and removed, and other unannotated RNAs (16,673,689) were obtained for *C. oleifera* on average (Table 4). The unannotated RNAs were subjected to further analyses for miRNA identification.

**Fig. 1:**
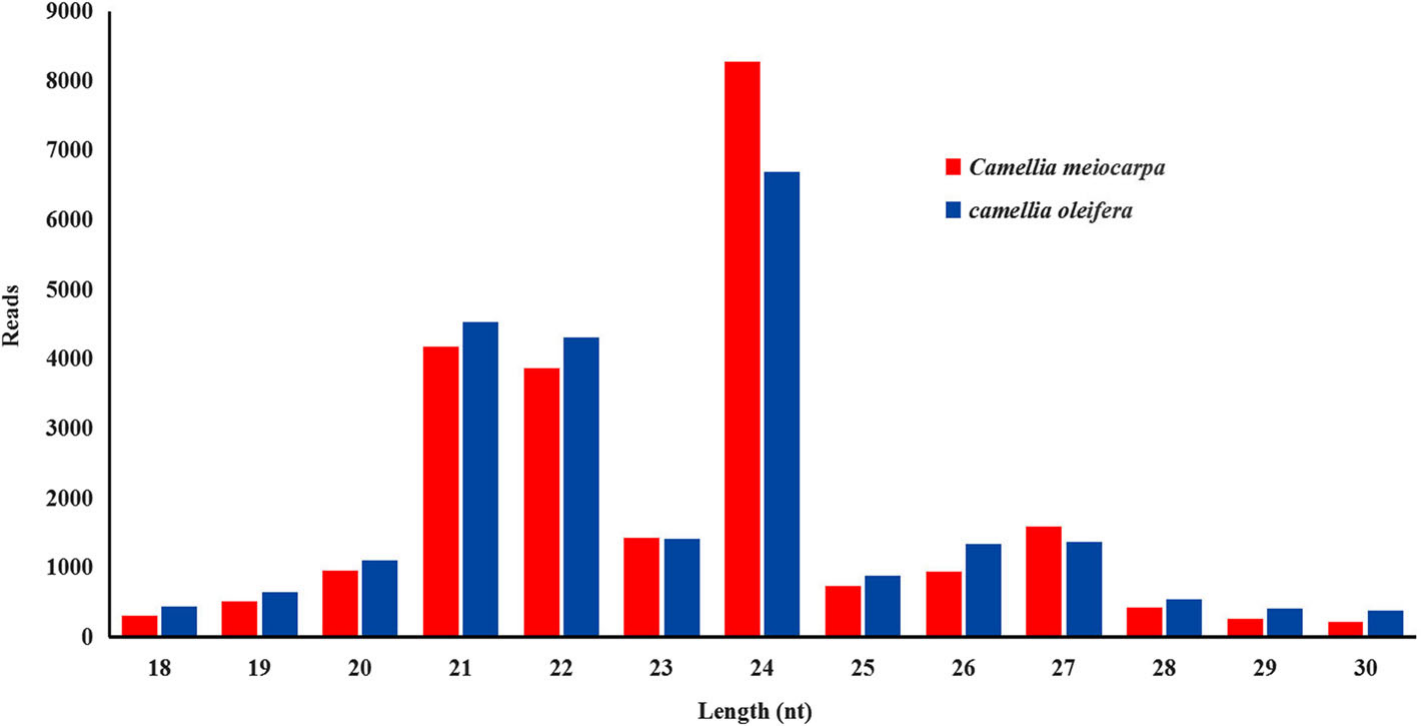
Length distributions of small RNAs in two camellia species. Data of *C. meiocarpa* and *C. oleifera* were averaged across five level moisture content (50, 40, 30, 20 and 10%) for each species separately

The majority of small RNAs were 21 to 24 nt in length, producing similar length distributions in both species (Fig. 1). The 24 nt small RNAs were the most abundant representing 35.07 and 27.85% of small RNAs in *C. meiocarpa* and *C. oleifera*, respectively, the second was 21 nt representing 17.72 and 18.85%, third was 22 nt representing 16.35 and 17.97% (Additional file 1: Tables S3 and S4). The 40% moisture level (sample S02) for *C. meiocarpa*, produced the highest 24-nt RNA peak while 10% moisture level (sample S05) produced the highest 21-nt and 22-nt RNA peaks among the studied moisture content levels (Additional file 1: Table S3), conversely, 50% moisture (sample S06) produced the highest 24-nt, 21-nt, and 22-nt RNA peaks in *C. oleifera* (Additional file 1: Table S4).

### Identification of miRNAs during natural drying

We used the software miRDeep2 to map the retained sequence reads to identify candidate miRNAs. Across the five moisture content levels, *C. meiocarpa* produced successful 2,355,539 reads (11.51%) that were mapped to the plant genomes, of which the 10 and 50% moisture content levels (S05 and S01) produced the most (2,724,598) and least (1,902,986) mapped reads, respectively. Similarly, on average, *C. oleifera* produced 2,396,805 (14.37%) successful mapped reads, of which 50 and 20% moisture content levels (S06 and S09) produced most (2,803,519) and least (2,121,984) mapped reads. In total, 2,288,508 (12.80%) mapped reads were successfully annotated (Table 5).

**Table 5:** Alignment information statistics with the plant genomes

Species	Sample	Unannotated	Mapped reads	Percent
*C. meiocarpa*	S01	17,528,281	1,902,986	10.86
	S02	24,627,338	2,509,074	10.19
	S03	19,760,910	2,288,508	11.58
	S04	19,295,654	2,352,529	12.19
	S05	21,106,648	2,724,598	12.91
	Average	20,463,766	2,355,539	11.51
*C. oleifera*	S06	20,697,326	2,803,519	13.55
	S07	15,071,790	2,295,301	15.23
	S08	17,957,315	2,527,567	14.08
	S09	14,915,101	2,121,984	14.23
	S10	14,726,914	2,235,656	15.18
	Average	16,673,689	2,396,805	14.37
Two species		18,568,728	2,376,172	12.80

**Fig. 2:**
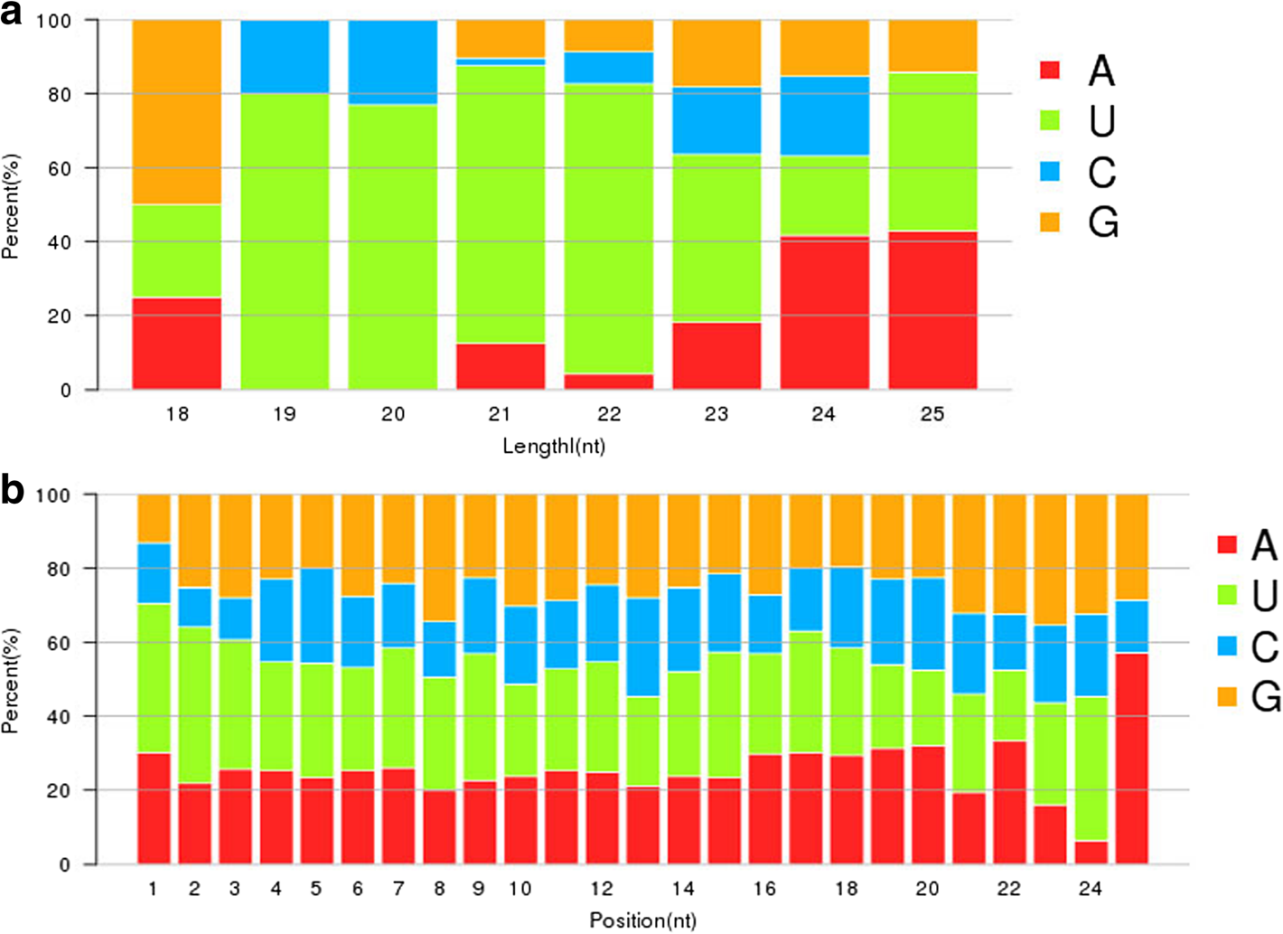
First nucleotide (**a**) and position nucleotide (**b**) biases of miRNA in two camellia species

A total of 274 candidate miRNAs, 248 and 246 unique sequences were assigned to *C. meiocarpa* and *C. oleifera*, respectively (Additional file 1: Table S5). Out of the identified candidate miRNA sequences, 110 were identified to 64 families, 57 families belonging to each of *C. meiocarpa* (99 out of 248 candidate miRNA) and *C. oleifera* (98 out of 246 candidate miRNA) (Additional file 1: Tables S5, S6 and S7). The diversity of miRNA families could be determined from their members’ number. As shown, MIR482 families were the largest with 10 members, followed by MIR159 and MIR535 with 5 members, and MIR160, MIR169_2 and MIR5272 with 4 members (Additional file 1: Table S6). Most of the conserved miRNA families had one member (65.63%) (Additional file 1: Table S6). The miRNAs sequences ranged in length from 18 to 25 nt, with a peak of 24 nt (Additional file 1: Figure S1). The miRNA first nucleotide preference distributions are show in Fig. 2a. miRNAs of 24 nt tended to start with 5′-A while the 21 nt tended with 5′-U (Fig. 2a). Tall miRNAs tended to start with 5′-U and not 5′-G (Fig. 2b). During the seed natural drying process, the number of miRNAs across moisture content levels showed a decreasing trend which was followed by increase with a peak at 40% moisture content (S02) for *C. meiocarpa* (Table 6). *C. oleifera*, on the other hand, showed a trend of reduction, followed by increase, followed by reduction in the number of miRNAs across the studied moisture content levels with a pronounced peak at 50% moisture content (S06) (Table 6).

**Table 6:** Sum of annotated miRNA during camellia seed natural drying

Species	Sample	All miRNA	miRNA with target	Target gene
*C. meiocarpa*	S01	169	169	3541
	S02	196	196	5936
	S03	190	190	3768
	S04	183	183	3311
	S05	173	173	3523
	Total	248	248	6057
*C. oleifera*	S06	191	191	3738
	S07	173	173	3397
	S08	177	177	3616
	S09	178	178	3451
	S10	169	169	3545
	Total	246	246	4532
Two species		274	274	6250

Next we conducted sequence homology search of these candidate miRNAs against known mature miRNAs in miRBase. Any miRNA that met the sequence homology criteria of Yang et al. [35] and Jain et al. [36] was considered a conserved miRNA gene. Through this analysis, we identified a total of 151 conserved miRNAs which belonging to 47 miRNA families in both camellia species (Additional file 1: Table S6). miRDeep2 score above 1.0 resulted in 98 pre-miRNAs (64.90%) of which 35 with a read count ranging between 10 and 100 (23.19%), 41 with read count above 100 (27.15%), and 75 with read count below 10 (49.67%) (Additional file 1: Table S6).

Those miRNA sequences, which met the threshold of miRDeep2 analysis but did not have any known homologous miRNA gene families in miRBase, were further analyzed to predict novel miRNAs in the two camellia species. The remaining miRNAs, which met the total score of >0, were considered to be true novel miRNAs. A total 123 novel mature miRNAs sequences were discovered and belonged to 36 miRNA families in both camellia species (Additional file 1: Table S7). miRDeep2 score above 1.0 had 87 pre-miRNAs (70.73%). The majority of pre-miRNA (69) read count ranged from 10 to 100 (56.10%), followed by 35 precursors in above 100 (28.45%) and 19 precursors below 10 (15.45%) (Additional file 1: Table S7).

### Prediction and annotation of miRNAs target genes

To better understand the functions of the identified miRNAs, putative target genes were predicted using TargetFinder software [42]. A total of 6250 target genes were identified (Table 6). Only 3733 target unigenes (59.73%) were annotated by performing a BLASTX search against diverse protein databases, revealing that 1368 (21.89%), 2190 (35.04%), 901 (14.42%), 2160 (34.56%), 2730 (43.68%), 2735 (43.76%), and 3718 (59.49%) unigenes have significant matches with sequences in COG, GO, KEGG, KOG, Pfam, Swissprot, and nr protein databases respectively (Table 7). A total of 2743 (73.48%) annotated target genes had the length of ≥1000 (Table 7).

**Table 7:** Functional annotation of the two camellia species

Database	Annotated Number	300 ≤ length < 1000	length ≥ 1000	%Annotated target gene
COG_Annotation	1368	174 (12.72%)	1194 (87.28%)	21.89
GO_Annotation	2190	469 (21.42%)	1721 (78.58%)	35.04
KEGG_Annotation	901	198 (21.98%)	703 (78.02%)	14.42
KOG_Annotation	2160	430 (19.91%)	1730 (80.09%)	34.56
Pfam_Annotation	2730	424 (15.53%)	2306 (84.47%)	43.68
Swissprot_Annotation	2735	584 (21.35%)	2151 (78.65%)	43.76
nr_Annotation	3718	977 (26.28%)	2741 (73.72%)	59.49
All_Annotated	3733	990 (26.52%)	2743 (73.48%)	59.73

**Fig. 3:**
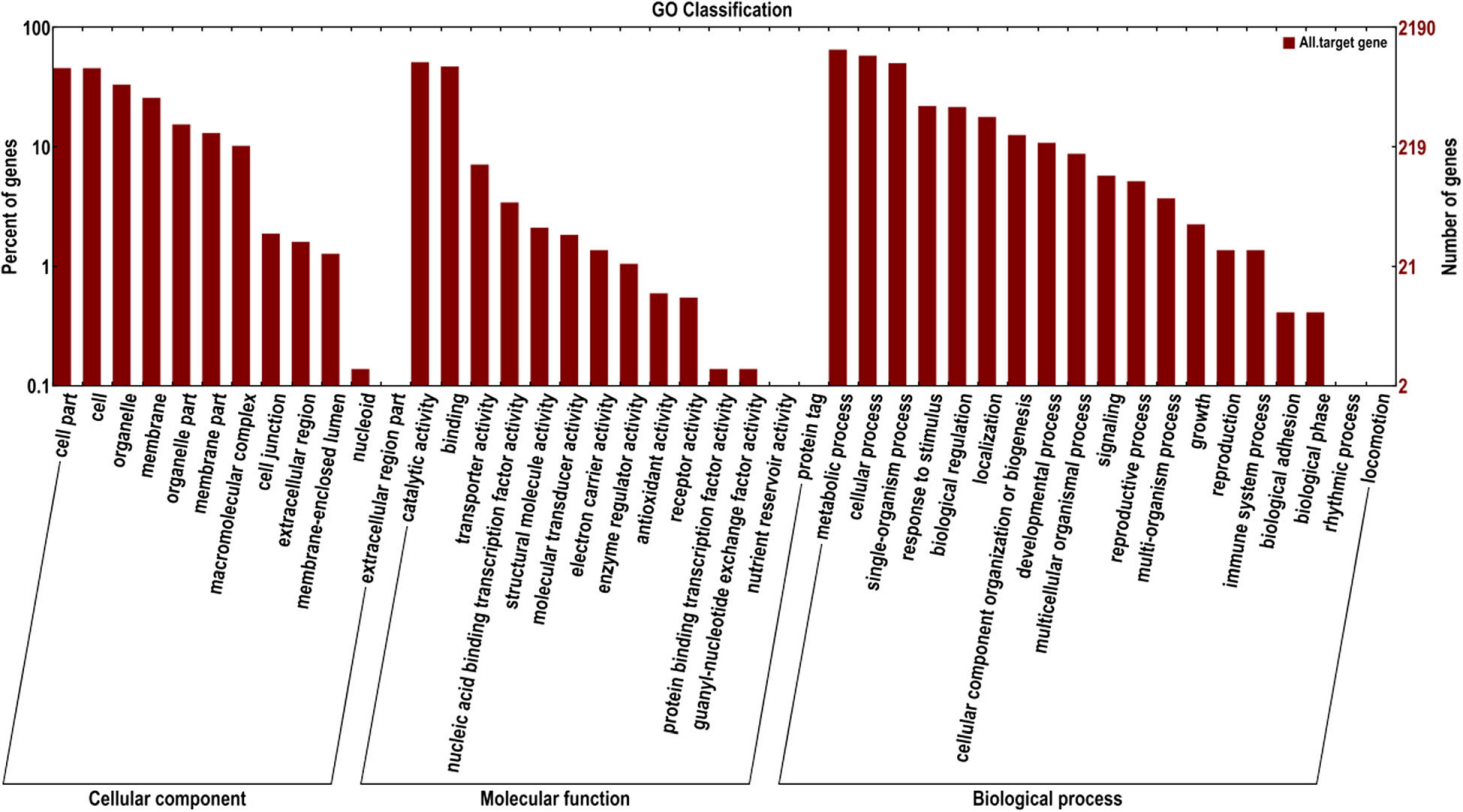
GO categories and distribution of miRNA targets in two camellia species

To evaluate the potential functions of these miRNA target genes, we next applied gene ontology (GO) and KEGG pathway analyses to categorize the miRNA targets. The miRNA target genes were categorized according to biological process, cellular component and molecular function by Go analysis (Fig. 3). A total of 1860 target genes were categorized into 19 biological process. Based on molecular function, 1722 target genes were classified to 14 categories. A total of 1212 target genes were categorized as cellular components. Target genes related to lipid metabolism were found among 51 GO terms, in which 18 GO terms are related to biological process and 33 related to molecular function. There were 31 miRNAs involved in lipid metabolism and targeted 148 unigenes (Additional file 1: Table S8). The KEGG enrichment analysis revealed 12 pathways related to lipid metabolism, involved in 15 miRNA targeted 93 unigenes. There were 19 target genes in Glycolysis/Gluconeogenesis pathway, 12 in Fatty acid biosynthesis pathway, and 7 in biosynthesis of unsaturated fatty acids pathway (Additional file 1: Table S9). Integrated GO and KEGG function annotation, identified 23 miRNA regulating 131 target genes that were annotated as lipid metabolism (Additional file 1: Table S10). These miRNAs regulated the changes of seed oil content at different moisture content levels. There were high correlations between transcript abundance with added value of oil content, for example MIR482 (Additional file 1: Table S11). Finally, miRNA of lipid metabolism only expressed were identified in *C. Meiocarpa* (Group1_Unigene_BMK.30485_635795) and *C. oleifera* (Group1_Unigene_BMK.37364_696840 and Group1_Unigene_BMK.38037_703962) (Table 8).

**Table 8:** miRNA of lipid metabolism transcript abundance in the two camellia species during seed natural drying

Pre-miRNA	miRNA	*C. meiocarpa*						*C. oleifera*					
		S01	S02	S03	S04	S05	Average	S06	S07	S08	S09	S10	Average
CL22479Contig1_234494	Unkown	0	0	0	1	0	0.2	1	0	1	0	1	0.6
CL22738Contig1_61999	Unkown	0	0	1	0	0	0.2	0	0	0	0	1	0.2
Group1_Unigene_BMK.23434_588836	MIR5067	20	21	33	25	24	24.6	19	20	15	9	10	14.6
Group1_Unigene_BMK.30485_635795	Unkown	0	1	0	0	0	0.2	0	0	0	0	0	0.0
Group1_Unigene_BMK.37364_696840	Unkown	0	0	0	0	0	0.0	1	2	0	0	0	0.6
Group1_Unigene_BMK.37987_703484	MIR2118	302	563	747	807	602	604.2	650	280	434	325	359	409.6
Group1_Unigene_BMK.38037_703962	Unkown	0	0	0	0	0	0.0	0	0	0	0	1	0.2
Group2_Unigene_BMK.25259_1025465	MIR482	302	563	747	807	602	604.2	650	280	434	325	359	409.6
Group2_Unigene_BMK.25259_1025498	MIR482	302	563	747	807	602	604.2	650	280	434	325	359	409.6
Group2_Unigene_BMK.34335_1,093,229	MIR5067	20	21	33	25	24	24.6	19	20	15	9	10	14.6
Group2_Unigene_BMK.50808_1268477	Unkown	0	0	0	1	0	0.2	1	0	0	1	0	0.4
Group2_Unigene_BMK.9543_1378570	Unkown	15	22	11	26	21	19.0	17	10	15	11	7	12.0
CL19455Contig1_54014	Unkown	26	26	23	22	27	24.8	40	18	28	25	14	25.0
CL19777Contig1_314088	Unkown	23	34	30	29	26	28.4	35	18	31	26	20	26.0
CL2440Contig1_359627	MIR1861	2	6	13	5	4	6.0	5	1	5	3	4	3.6
CL4378Contig1_191450	Unkown	11	8	10	14	15	11.6	35	13	22	17	12	19.8
CL9644Contig1_380257	Unkown	26	24	21	21	18	22.0	20	12	18	10	24	16.8
Group1_Unigene_BMK.45675_802511	Unkown	4	4	8	1	10	5.4	9	4	4	5	3	5.0
Group2_Unigene_BMK.24252_1018543	Unkown	1	1	1	0	0	0.6	1	2	2	1	0	1.2
Group2_Unigene_BMK.38504_1137258	Unkown	35	31	29	40	33	33.6	45	25	24	13	25	26.4
Group2_Unigene_BMK.38504_1137263	Unkown	6	3	4	3	4	4.0	2	0	2	2	1	1.4
Group2_Unigene_BMK.63506_1315063	Unkown	58	47	89	49	53	59.2	96	49	34	50	20	49.8
Group2_Unigene_BMK.39605_1150110	Unkown	0	1	1	0	0	0.4	2	1	0	1	0	0.8

### Differentially expressed miRNAs of lipid metabolism during natural drying

The differences between the miRNA profiles of the two camellia species are possibly related to differences in their responses to natural drying and were investigated using the IDEG6 software. For *C. Meiocarpa,* miRNA abundance pairwise analysis between the different moisture content libraries, indicated that 40, 46, 63, and 38 significantly differentially expressed miRNAs were identified between 40 and 50% (S01 vs. S02), 30–40% (S02 vs. S03), 20–30% (S03 vs. S04), and 10–20% (S04 vs. S05) moisture contents, respectively. The highest number of significantly differentially expressed miRNAs was observed between 20 and 30% (S03 vs. S04) moisture contents, with the 1owest up- and highest down-regulated numbers of 10 and 53, respectively (Table 9), with 9 significantly different miRNAs involved in lipid metabolism (Table 10 and Additional file 1: Table S12). *C. oleifera* produced similar results with 71, 54, 36, and 56 significantly differentially expressed miRNAs observed between 40 and 50% (S06 vs. S07), 30–40% (S07 vs. S08), 20–30% (S08 vs. S09), and 10–20% (S09 vs. S10) moisture contents, respectively. The highest number of significantly differentially expressed miRNAs was observed between 40 and 50% (S06 vs. S07) moisture contents, with the highest up- and lowest down-regulated numbers of 61 and 10, respectively (Table 9), with 8 significantly different miRNAs involved in lipid metabolism (Tables 11 and S13).

**Table 9:** Comparison of the number of differentially expressed miRNAs between samples with different moisture content within and across the two camellia species during seed natural drying

Type	Compared type	Number	Up-regulated	Down-regulated
*C. meiocarpa*	S01 vs. S02	40	21	19
	S02 vs. S03	46	20	26
	S03 vs. S04	63	10	53
	S04 vs. S05	38	29	9
	Average	47	20	27
*C. oleifera*	S06 vs. S07	71	61	10
	S07 vs. S08	54	9	45
	S08 vs. S09	36	19	17
	S09 vs. S10	56	16	40
	Average	54	26	28
Between species	S06. vs. S01	78	69	9
	S07 vs. S02	51	22	29
	S08 vs. S03	44	27	17
	S09 vs. S04	58	19	39
	S10 vs. S05	61	44	17
	Average	58	36	22

**Table 10:** Significant differentially expressed miRNAs of lipid metabolism during *C. meiocarpa* seed natural drying across different moisture content levels (%)^1^

Pre-miRNA	50 vs. 40%	40 vs. 30%	30 vs. 20%	20 vs. 10%
Group2_Unigene_BMK.25259_1025498	1	1	1	0
Group1_Unigene_BMK.37987_703484	1	1	1	0
Group2_Unigene_BMK.34335_1,093,229	0	0	1	0
CL2440Contig1_359627	0	0	1	0
Group1_Unigene_BMK.23434_588836	0	0	1	0
CL19777Contig1_314088	0	0	1	0
Group2_Unigene_BMK.25259_1025465	1	1	1	0
Group1_Unigene_BMK.45675_802511	0	0	1	1
Group2_Unigene_BMK.63506_1315063	0	1	1	0

1 Represents significant DEG and 0 represents non-significant DEG

^1^significance at *P* < 0.05

**Table 11:** Significant differentially expressed miRNAs of lipid metabolism during *C. oleifera* seed natural drying across different moisture content levels (%)^1^

Pre-miRNA	50 vs. 40%	40 vs. 30%	30 vs. 20%	20 vs. 10%
Group2_Unigene_BMK.63506_1315063	1	1	0	1
CL19455Contig1_54014	0	0	0	1
Group1_Unigene_BMK.37987_703484	1	1	0	1
Group1_Unigene_BMK.23434_588836	1	1	0	0
Group2_Unigene_BMK.25259_1025498	1	1	0	1
Group2_Unigene_BMK.25259_1025465	1	1	0	1
Group2_Unigene_BMK.38504_1137258	1	1	0	0
Group2_Unigene_BMK.34335_1,093,229	1	1	0	0

1 Represents significant DEG and 0 represents non-significant DEG

^1^significance at *P* < 0.05

Comparing across the two species, the average number of miRNAs in *C. oleifera* seeds was higher than that of *C. Meiocarpa* (Table 9). Pairwise analysis of miRNA abundance between the two species for the same moisture level libraries indicated that there were 78, 51, 44, 58, and 61 significant differentially expressed miRNAs for the 50, 40, 30, 20, and 10% moisture contents, respectively. There were three differentially expressed miRNAs of lipid metabolism during the seed natural drying process of the studied two camellia species (Group1_Unigene_BMK.37987_703484, Group2_Unigene_BMK.25259_1025465, and Group2_Unigene_BMK.25259_1025498) (Tables 12 and S14). The highest up- (69) and lowest down-regulated number (9) of significant differentially expressed miRNAs were detected for 50% moisture contents (S06 vs. S01) (Table 9). This indicated that the greatest difference between the two species was observed at the 50% moisture content, with 12 significant differentially expressed miRNAs regulating lipid metabolism during seed natural drying (Tables 12 and S14).

**Table 12:** Significant differentially expressed miRNAs of lipid metabolism during the two camellia species seed natural drying across the same moisture content level (*C. oleifera* vs. *C*. *meiocarpa* MC%)^1^

Pre-miRNA	50 vs. 50%	40 vs. 40%	30 vs. 30%	20 vs. 20%	10 vs. 10%
CL19777Contig1_314088	1	0	0	0	0
CL19455Contig1_54014	1	0	0	0	0
Group2_Unigene_BMK.25259_1025465	1	1	1	1	1
Group2_Unigene_BMK.9543_1378570	1	0	0	0	0
CL9644Contig1_380257	1	0	0	0	0
Group2_Unigene_BMK.25259_1025498	1	1	1	1	1
Group2_Unigene_BMK.38504_1137258	1	0	0	0	0
Group2_Unigene_BMK.34335_1,093,229	1	0	1	0	0
Group2_Unigene_BMK.63506_1315063	1	0	1	1	1
Group2_Unigene_BMK.38504_1137263	1	0	0	0	0
Group1_Unigene_BMK.23434_588836	1	0	1	0	0
Group1_Unigene_BMK.37987_703484	1	1	1	1	1

^1^Represents significant DEG and ^0^represents non-significant DEG

^1^significance at *P* < 0.05

### Validation of the expression patterns of differentially expressed miRNAs related lipid metabolism by RT-qPCR

**Fig. 4:**
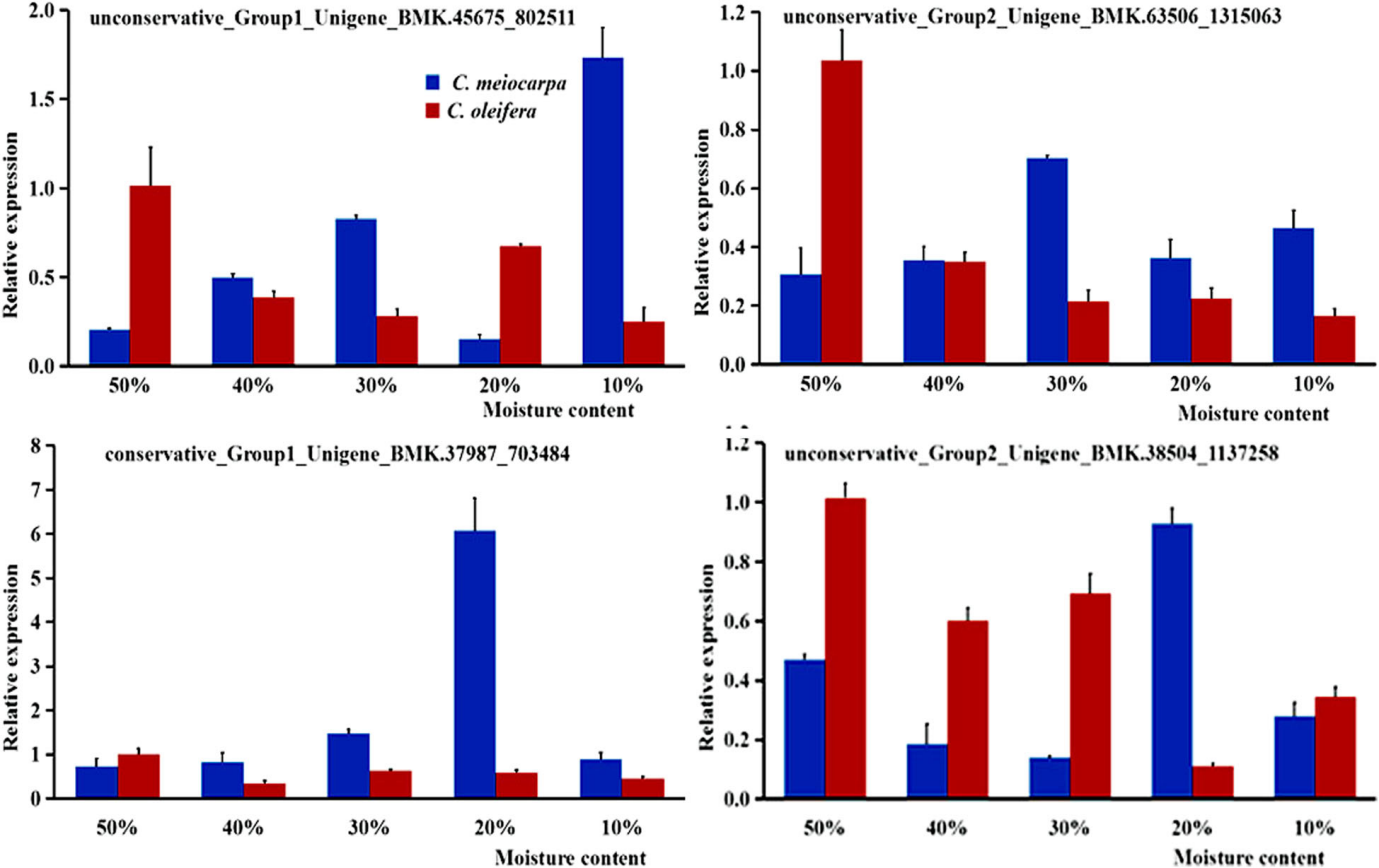
qRT-PCR validation of miRNA in *C. meiocarpa* and *C. oleifera*. Relative expression of miRNA in 10, 20, 30, 40, and 50% moisture contents. Reference gene was 5.8 rRNA. Normalized miRNA in 50% moisture content of *C. oleifera* were arbitrarily set to 1. *Error bars* were calculated based on three replicates

To validate the data obtained from the high-throughput sequencing, four significantly differentially expressed miRNAs (Group1_Unigene_BMK.45675_802511, Group2_Unigene_BMK.63506_1315063, Group1_Unigene_BMK.37987_703484, and Group2_Unigene_BMK.38504_1137258) were predicted to target genes involved in lipid metabolism and their expression levels were quantified using stem-loop qRT-PCR (Fig. 4). The results were consistent with deep sequencing data (Table 8) and the majority of analyzed miRNAs showed moisture content- and species-specific expression. For *C*. *meiocarpa*, Group1_Unigene_BMK.45675_802511 and Group2_Unigene_BMK.63506_1315063 peaked at 10 and 30% moisture content while the other two miRNAs (Group1_Unigene_BMK.37987_703484, and Group2_Unigene_BMK.38504_1137258) peaked at 20% moisture content. Additionally, all four miRNAs had different lowest point (Fig. 4). *C. oleifera* showed four miRNAs peaked at 50% moisture content but had different lowest point at 10 (Group1_Unigene_BMK.45675_802511 and Group2_Unigene_BMK.63506_1315063), 20 (Group2_Unigene_BMK.38504_1137258), and 40% (Group1_Unigene_BMK.37987_703484) moisture content (Fig. 4). These expression patterns indicate that lipid metabolism of the two camellia species were regulated by miRNA during the seed natural drying process.

## Discussion

### miRNAs related to the lipid metabolism of camellia species

Recently, sequencing of oil crops produced a large amount of miRNA associated with lipid metabolism-related genes (e.g., 97, 30, 10, and 4 miRNAs targeting 89, 133, 21, and 4 lipid biosynthesis genes were reported for soybean [12], *Camelina sativa* [11], castor bean [49], and peanut [19], respectively). In the present study, we detected 23 pre-miRNAs targeting 131 candidate genes regulating lipid metabolism functions during camellia seed natural drying (Additional file 1: Table S10). Of these pre-miRNAs, 5, 5, and 11 regulated fatty acid biosynthesis, accumulation, and catabolism, respectively, and additional 2 (Group1_Unigene_BMK.30485_635795 and Group1_Unigene_BMK.45675_802511) regulated not only fatty acid biosynthesis and accumulation, but also fatty acid catabolism (Additional file 1: Table S10). Group1_Unigene_BMK.30485_635795 and Group1_Unigene_BMK.45675_802511 encode 56 and 41 target genes involved in fatty acid biosynthesis, accumulation, and catabolism, respectively (Additional file 1: Table S10).

Concurring with the above, the most abundant miRNAs (osa-miR2118e: Group1_Unigene_BMK.37987_703484, Group2_Unigene_BMK.25259_1025465, and Group2_Unigene_BMK.25259_1025498 (Additional file 1: Table S10 and Table 8)) that target genes encoding a long-chain-alcohol oxidase involving fatty acid-oxidation [50]. The second abundant miRNAs (Group2_Unigene_BMK.63506_1315063) targeting diacylglycerol kinase, a lipid kinase converting diacylglycerol to phosphatidic acid, regulating balance of diacylglycerol and phosphatidic acid [51], and potentially involved in regulating lipid deposition [52]. The third, Group2_Unigene_BMK.38504_1137258 (Additional file 1: Table S10 and Table 8), targeting lipases/acylhydrolase, fatty acid hydroxylase, and carboxylesterase, which are hydrolytic enzymes involved in degradation of fatty acid [53–55], collectively suggesting that these miRNAs may play important roles in decreasing the rate of lipid breakdown during camellia seed natural drying. This can be confirmed by the 4% increase in oil content in two camellia seed through seed nature drying (Table 1).

### miRNAs regulate lipid metabolism during camellia seed natural drying

Several studies have shown that miRNAs are differentially regulated in response to stress [56] and that this differential regulation varied among different plant species [57]. For example, distinct responses to drought stresses were reported for miRNAs in *Arabidopsis* [58], switchgrass [59], *Populus* [60] and *Caragana intermedia* [61]. Especially, drought stress in switchgrass [59] and *Populus* [60] were regulated by miRNAs related to lipid metabolism; however, the linkage between drought responses and lipid metabolism miRNAs changes is not established.

In the present study, *C. Meiocarpa* produced 9 significant differentially expressed miRNAs regulating lipid metabolism with only 4 at 20–30% moisture contents. These pre-miRNAs belonged to Group2_Unigene_BMK.34335_1,093,229, Group1_Unigene_BMK.23434_588836, CL19777_Contig1_314088, and CL2440_Contig1_359627, with the former three showing more than 100 TPM (Table 10 and Additional file 1: Table S12) and targeting 3-ketoacyl-CoA synthase III, which catalyze the initial elongation step of fatty acid biosynthetic process [62] and glycerol-3-phosphate transporter, a precursor protein for phospholipid biosynthesis [63]. It is interesting to note that these three pre-miRNAs were down-regulated resulting in a reduction in the fatty acid biosynthesis, so seeds with 30% moisture content have high fatty acid synthesis and accumulation activities (Table 1).

Similarly, *C. oleifera* produced 8 significant differentially expressed miRNAs during seed natural drying, with up- and down-regulated for the 40–50 and 30–40% moisture contents, respectively (Tables 11 and S13). The largest fold changes were observed for Group1_Unigene_BMK.23434_588836 and Group2_Unigene_BMK.34335_1,093,229, which target 3-ketoacyl-CoA synthase III (Additional file 1: Table S10 and Table S13). These pre-miRNAs regulate fatty acid biosynthesis with seeds at 40–50% moisture content showing low fatty acid biosynthesis activities while those at 30–40% moisture content exhibiting high activities (Table S13). This can be confirmed by the observed 1.06 and 9.17% increase in oil content at 40–50 and 30–40% moisture contents, and reaching the highest point at 30% moisture content (Table 1). So seeds with 30% moisture content have also high fatty acid synthesis and accumulation activities.

Comparing the significant differentially expressed miRNAs of *C. meiocarpa* (9) with those of *C. oleifera* (8), indicated that the two species share 6 miRNAs involved in lipid metabolism*,* with unique miRNAs belonging to each species (CL19455Contig1_54014 and Group2_Unigene_BMK.38504_1137258 in *C. oleifera*, and CL2440Contig1_359627, CL19777Contig1_314088 and Group1_Unigene_BMK.45675_802511 in *C. meiocarpa*) (Tables 10 and 11). Group2_Unigene_BMK.38504_1137258 and CL2440Contig1_359627 regulated fatty acid catabolism, CL19455Contig1_54014 and CL19777Contig1_314088 regulated fatty acid accumulation, and Group1_Unigene_BMK.45675_802511 regulated fatty acid synthesis, accumulation, and catabolism (Additional file 1: Table S10). These indicate that oil content differences between the two camellia species are mainly due to their differential abilities miRNAs of fatty acid accumulation and catabolism.

Collectively, the studied two camellia species produced 12 significant differentially expressed miRNAs to regulate lipid metabolism during seed natural drying (Tables 12 and S14). These pre-miRNAs indicated that *C*. *meiocarpa* has higher activities to regulate the lipid metabolism and this can be confirmed by its lower oil content as compared to *C. oleifera* (Table 1). It should be stated that all these 12 differentially expressed miRNAs were significantly expressed in the 50% moisture content stage (Tables 12 and S14). Seeds with 50% moisture content had only significant differentially expressed pre-miRNAs (CL19777Contig1_314088, CL19455Contig1_54014, Group2_Unigene_BMK.9543_1378570, CL9644Contig1_380257, Group2_Unigene_BMK.38504_1137258 and Group2_Unigene_BMK.38504_1137263) (Tables 8 and S14). CL19777Contig1_314088 and CL19455Contig1_54014 target glycerol-3-phosphate transporter (Additional file 1: Table S10). Group2_Unigene_BMK.9543_1378570 target acetyl-CoA-carboxylase (Additional file 1: Table S10), which catalyze the ATP-dependent carboxylation of acetyl-CoA to form malonyl-CoA, the rate limiting and first committed reaction in fatty acid synthesis [64]. So these three pre-miRNA control different fatty acid synthesis and accumulation in the two camellia species. For all significant differentially expressed miRNAs, the largest fold change was observed for Group2_Unigene_BMK.38504_1137263 (Fatty acid hydroxylase), followed by CL9644Contig1_380257 (Carboxylesterase), and Group1_Unigene_BMK.23434_588836 (3-ketoacyl-CoA synthase III) and Group2_Unigene_BMK.34335_1,093,229 (3-ketoacyl-CoA synthase III) (Additional file 1: Table S10 and Table S14). So the oil content of *C. oleifera* is higher than *C. meiocarpa* and this is attributable to four pre-miRNAs, of which Group2_Unigene_BMK.38504_1137263 and CL9644Contig1_380257 regulating fatty acid catabolism and Group1_Unigene_BMK.23434_588836 and Group2_Unigene_BMK.34335_1,093,229 regulating fatty acid synthesis.

## Conclusion

The present study identified 274 candidate miRNAs, with 248 and 246 unique sequences to *C. meiocarpa* and *C. oleifera*, respectively*.* Integrated GO and KEGG function annotation, produced 23 miRNAs regulating 131 target genes, all were annotated as lipid metabolism, regulating fatty acid biosynthesis, accumulation and catabolism during seed natural drying. Lipid metabolism primarily focused on fatty acid catabolism, with five miRNAs (Group1_Unigene_BMK.37987_703484, Group2_nigene_BMK.25259_1025465 Group2_Unigene_BMK.25259_1025498, Group2_Unigene_BMK.63506_1315063, and Group2_Unigene_BMK.38504_1137258) playing important roles in decreasing the rate of lipid breakdown and additional two miRNAs (Group2_Unigene_BMK.34335_1,093,229 and Group1_Unigene_BMK.23434_588836) regulating fatty acid synthesis. Across the two species, four pre-miRNAs were identified to regulate lipid metabolism, with Group2_Unigene_BMK.38504_1137263 and CL9644Contig1_380257, and Group1_Unigene_BMK.23434_588836 and Group2_Unigene_BMK.34335_1,093,229 regulating fatty acid catabolism and synthesis, respectively.

To our knowledge, this work provides the first small RNA expression analysis of lipid metabolism in camellia seed during natural drying as well as the first comparative miRNA profiling analysis between *C. Meiocarpa* and *C. oleifera* that exhibit significantly different fatty acid profiles.

## Additional file

Additional file 1: Figure S1. Length distributions of miRNAs in two camellia species. **Table S1.** qRT-PCR primer sequences. **Table S2.** Length distributions of small RNAs in *C. meiocarpa* during seed natural drying. **Table S3.** Length distributions of small RNAs in *C. oleifera* during seed natural drying. **Table S4.** miRNAs transcript abundance in the two camellia species during seed natural drying. **Table S5.** All conservative miRNA discovered in the two camellia species during seed natural drying. **Table S6.** All novel miRNA discovered in the two camellia species during seed natural drying. **Table S7.** GO terms related with the lipid metabolism in the two camellia species during seed natural drying. **Table S8.** KEGG enrichment related with the lipid metabolism in the two camellia species during seed natural drying. **Table S9.** miRNA of lipid metabolism targets and their putative functions. **Table S10.** Differentially expressed miRNAs of lipid metabolism during *C. meiocarpa* seed natural drying. **Table S11.** Differentially expressed miRNAs of lipid metabolism during *C. oleifera* seed natural drying. **Table S12.** Differentially expressed miRNAs of lipid metabolism between two camellia species during seed natural drying. (DOCX 168 kb)
