# Supplementary material for: High throughput sequencing of small RNAs reveals dynamic microRNAs expression of lipid metabolism during Camellia oleifera and C. meiocarpa seed natural drying
Source: BMC Genomics. 2017 Jul 20;18:546. doi: 10.1186/s12864-017-3923-z (PMC5520325; doi:10.1186/s12864-017-3923-z)
Supplement: Additional file 1: Figure S1. — Length distributions of miRNAs in two camellia species. Table S1. qRT-PCR primer sequences. Table S2. Length distributions of small RNAs in C. meiocarpa during seed natural drying. Table S3. Length distributions of small RNAs in C. oleifera during seed natural drying. Table S4. miRNAs transcript abundance in the two camellia species during seed natural drying. Table S5. All conservative miRNA discovered in the two camellia species during seed natural drying. Table S6. All novel miRNA discovered in the two camellia species during seed natural drying. Table S7. GO terms related with the lipid metabolism in the two camellia species during seed natural drying. Table S8. KEGG enrichment related with the lipid metabolism in the two camellia species during seed natural drying. Table S9. miRNA of lipid metabolism targets and their putative functions. Table S10. Differentially expressed miRNAs of lipid metabolism during C. meiocarpa seed natural drying. Table S11. Differentially expressed miRNAs of lipid metabolism during C. oleifera seed natural drying. Table S12. Differentially expressed miRNAs of lipid metabolism between two camellia species during seed natural drying. (DOCX 168 kb) [file 12864_2017_3923_MOESM1_ESM.docx]

Additional file 1

Figure S1. Length distributions of miRNAs in two camellia species.

| Table S1. qRT-PCR primer sequences | | |
| --- | --- | --- |
| **miRNA** | **Forward primer (5'-3')** | **Reverse primer (5'-3')** |
| Group1_Unigene_BMK.45675_802511 | AGAGAGAGAGAGAGAGAGAGAGTG | CGTCGAGCATTTGAATTTCTAGA |
| Group2_Unigene_BMK.63506_1315063 | GGGAAAGGGAAAGGGAAAAGGT | CGTCGAGCATTTGAATTTCTAGA |
| Group1_Unigene_BMK.37987_703484 | TTCCCAATCCCTCCCATTCCTA | CGTCGAGCATTTGAATTTCTAGA |
| Group2_Unigene_BMK.38504_1137258 | GGCTTGTGTTTGGATCATGGATTT | CGTCGAGCATTTGAATTTCTAGA |
| 5.8s | ACGTCTGCCTGGGTGTCACAA | GCGAGCACAGAATTAATACGAC |

| Table S2. Length distributions of small RNAs in *C.* *meiocarpa* during seed natural drying | | | | | | | |
| --- | --- | --- | --- | --- | --- | --- | --- |
| **Length** | **S01** | **S02** | **S03** | **S04** | **S05** | **Average** | **%** |
| **18** | 25,742 | 17,268 | 32,391 | 33,165 | 40,392 | 29,792 | 1.26 |
| **19** | 44,009 | 33,246 | 51,240 | 54,284 | 65,675 | 49,691 | 2.11 |
| **20** | 80,441 | 67,641 | 98,916 | 107,604 | 117,595 | 94,439 | 4.01 |
| **21** | 310,038 | 381,491 | 390,554 | 499,086 | 505,499 | 417,334 | 17.72 |
| **22** | 295,263 | 388,667 | 343,701 | 440,054 | 457,902 | 385,117 | 16.35 |
| **23** | 130,122 | 140,943 | 160,214 | 127,681 | 151,193 | 142,031 | 6.03 |
| **24** | 685,602 | 1,017,513 | 844,941 | 747,709 | 834,473 | 826,048 | 35.07 |
| **25** | 59,211 | 79,493 | 67,813 | 70,030 | 85,407 | 72,391 | 3.07 |
| **26** | 76,921 | 118,113 | 73,737 | 80,034 | 120,837 | 93,928 | 3.99 |
| **27** | 123,739 | 172,401 | 146,802 | 112,966 | 232,639 | 157,709 | 6.70 |
| **28** | 33,822 | 42,395 | 38,395 | 35,814 | 54,529 | 40,991 | 1.74 |
| **29** | 21,121 | 27,987 | 21,739 | 23,880 | 31,123 | 25,170 | 1.07 |
| **30** | 16,955 | 21,916 | 18,065 | 20,222 | 27,334 | 20,898 | 0.89 |

| Table S3. Length distributions of small RNAs in *C.* *oleifera* during seed natural drying | | | | | | | |
| --- | --- | --- | --- | --- | --- | --- | --- |
| **Length** | **S06** | **S07** | **S08** | **S09** | **S10** | **Average** | **%** |
| **18** | 56,633 | 19,942 | 36,221 | 42,514 | 61,712 | 43,404 | 1.81 |
| **19** | 80,563 | 30,746 | 58,057 | 62,705 | 87,756 | 63,965 | 2.67 |
| **20** | 161,725 | 61,344 | 103,029 | 95,831 | 128,096 | 110,005 | 4.59 |
| **21** | 675,038 | 313,783 | 464,975 | 375,088 | 429,636 | 451,704 | 18.85 |
| **22** | 554,543 | 383,253 | 479,922 | 321,414 | 414,228 | 430,672 | 17.97 |
| **23** | 150,993 | 131,344 | 152,745 | 129,256 | 139,280 | 140,724 | 5.87 |
| **24** | 796,468 | 672,798 | 734,122 | 601,654 | 532,217 | 667,452 | 27.85 |
| **25** | 67,899 | 91,663 | 98,852 | 90,147 | 87,604 | 87,233 | 3.64 |
| **26** | 72,838 | 202,152 | 133,665 | 137,632 | 122,307 | 133,719 | 5.58 |
| **27** | 96,843 | 174,320 | 135,249 | 136,354 | 135,196 | 135,592 | 5.66 |
| **28** | 36,991 | 81,647 | 56,563 | 51,764 | 42,205 | 53,834 | 2.25 |
| **29** | 28,706 | 66,598 | 38,734 | 39,241 | 30,231 | 40,702 | 1.70 |
| **30** | 24,279 | 65,711 | 35,433 | 38,384 | 25,188 | 37,799 | 1.58 |

| Table S4. miRNAs transcript abundance in the two camellia species during seed natural drying | | | | | | | | | | | | |
| --- | --- | --- | --- | --- | --- | --- | --- | --- | --- | --- | --- | --- |
| **Number** | **Pre-miRNA** | **S01** | **S02** | **S03** | **S04** | **S05** |  | **S06** | **S07** | **S08** | **S09** | **S10** |
|  |  | ***C. Meiocarpa*** | | | | |  | ***C. oleifera*** | | | | |
| 1 | CL10456Contig1_470022 | 1 | 0 | 0 | 1 | 0 |  | 1 | 0 | 0 | 0 | 0 |
| 2 | CL14260Contig1_478773 | 0 | 0 | 0 | 0 | 1 |  | 0 | 0 | 0 | 0 | 0 |
| 3 | CL17638Contig1_50456 | 507 | 451 | 888 | 1,285 | 1,228 |  | 1,548 | 388 | 1,067 | 762 | 921 |
| 4 | CL17766Contig1_485769 | 12 | 2 | 8 | 10 | 10 |  | 8 | 18 | 23 | 9 | 13 |
| 5 | CL18763Contig1_311943 | 270 | 18 | 32 | 46 | 79 |  | 126 | 46 | 25 | 105 | 45 |
| 6 | CL19035Contig1_399746 | 0 | 1 | 2 | 1 | 0 |  | 0 | 1 | 1 | 0 | 1 |
| 7 | CL1911Contig1_184453 | 0 | 0 | 0 | 0 | 3 |  | 0 | 0 | 0 | 0 | 0 |
| 8 | CL19817Contig1_140074 | 7,270 | 7,937 | 12,411 | 26,950 | 18,464 |  | 47,498 | 5,880 | 14,979 | 13,793 | 22,552 |
| 9 | CL19869Contig1_140190 | 0 | 0 | 2 | 0 | 0 |  | 0 | 0 | 0 | 0 | 0 |
| 10 | CL19882Contig1_401776 | 1 | 0 | 0 | 0 | 0 |  | 1 | 0 | 0 | 0 | 0 |
| 11 | CL19980Contig1_228041 | 0 | 1 | 1 | 0 | 0 |  | 0 | 0 | 0 | 0 | 0 |
| 12 | CL20032Contig1_314672 | 1 | 1 | 0 | 0 | 0 |  | 0 | 0 | 0 | 0 | 0 |
| 13 | CL21369Contig1_405539 | 10,283 | 11,269 | 16,998 | 37,052 | 26,522 |  | 61,649 | 9,194 | 22,808 | 20,517 | 29,634 |
| 14 | CL2149Contig1_11711 | 10 | 12 | 18 | 20 | 16 |  | 28 | 10 | 8 | 16 | 20 |
| 15 | CL2149Contig1_11712 | 10 | 12 | 18 | 20 | 16 |  | 28 | 10 | 8 | 16 | 20 |
| 16 | CL21889Contig1_319550 | 0 | 0 | 0 | 0 | 2 |  | 0 | 0 | 0 | 0 | 0 |
| 17 | CL2190Contig1_98714 | 8,632 | 9,522 | 10,111 | 17,237 | 15,797 |  | 13,915 | 8,478 | 13,130 | 10,135 | 10,001 |
| 18 | CL21958Contig1_407004 | 0 | 0 | 0 | 0 | 0 |  | 2 | 1 | 1 | 0 | 0 |
| 19 | CL22098Contig1_407310 | 1,137 | 1,216 | 1,200 | 1,982 | 1,643 |  | 735 | 1,191 | 1,664 | 915 | 999 |
| 20 | CL22479Contig1_234494 | 0 | 0 | 0 | 1 | 0 |  | 1 | 0 | 1 | 0 | 1 |
| 21 | CL22574Contig1_61569 | 2,691 | 2,608 | 4,023 | 4,580 | 4,292 |  | 7,623 | 2,373 | 5,191 | 3,364 | 3,987 |
| 22 | CL22681Contig1_147702 | 0 | 0 | 1 | 0 | 0 |  | 0 | 0 | 0 | 0 | 0 |
| 23 | CL22738Contig1_61999 | 0 | 0 | 1 | 0 | 0 |  | 0 | 0 | 0 | 0 | 1 |
| 24 | CL22956Contig1_322565 | 0 | 0 | 2 | 0 | 0 |  | 0 | 0 | 0 | 0 | 0 |
| 25 | CL23613Contig1_324766 | 1 | 0 | 0 | 0 | 2 |  | 0 | 0 | 0 | 0 | 0 |
| 26 | CL24215Contig1_413571 | 10,283 | 11,269 | 16,998 | 37,052 | 26,522 |  | 61,649 | 9,194 | 22,808 | 20,517 | 29,634 |
| 27 | CL24280Contig1_502539 | 0 | 0 | 1 | 1 | 0 |  | 0 | 0 | 0 | 0 | 0 |
| 28 | CL25590Contig1_70607 | 1 | 3 | 0 | 2 | 0 |  | 0 | 0 | 1 | 0 | 0 |
| 29 | CL25590Contig1_70619 | 0 | 0 | 1 | 0 | 0 |  | 0 | 0 | 0 | 0 | 0 |
| 30 | CL26738Contig1_509111 | 27 | 25 | 41 | 47 | 34 |  | 71 | 21 | 31 | 40 | 36 |
| 31 | CL28847Contig1_77575 | 1 | 1 | 0 | 4 | 0 |  | 1 | 1 | 2 | 2 | 2 |
| 32 | CL29078Contig1_514119 | 2 | 4 | 9 | 14 | 26 |  | 12 | 5 | 0 | 3 | 8 |
| 33 | CL29150Contig1_514235 | 0 | 3 | 2 | 1 | 4 |  | 3 | 0 | 1 | 0 | 0 |
| 34 | CL29290Contig1_252046 | 141 | 182 | 236 | 234 | 280 |  | 114 | 116 | 195 | 149 | 55 |
| 35 | CL29784Contig1_515758 | 1 | 9 | 4 | 3 | 6 |  | 7 | 4 | 3 | 2 | 2 |
| 36 | CL30269Contig1_166900 | 1 | 0 | 0 | 0 | 0 |  | 0 | 0 | 0 | 0 | 0 |
| 37 | CL31317Contig1_256905 | 0 | 2 | 0 | 0 | 0 |  | 0 | 0 | 1 | 2 | 0 |
| 38 | CL4803Contig1_366120 | 0 | 0 | 2 | 0 | 0 |  | 1 | 0 | 0 | 0 | 0 |
| 39 | CL5425Contig1_367528 | 18 | 13 | 6 | 8 | 13 |  | 6 | 1 | 8 | 4 | 2 |
| 40 | CL5691Contig1_23074 | 0 | 0 | 0 | 0 | 0 |  | 2 | 0 | 0 | 0 | 0 |
| 41 | CL5786Contig1_196525 | 0 | 0 | 0 | 0 | 0 |  | 0 | 0 | 0 | 0 | 1 |
| 42 | CL6211Contig1_461735 | 1 | 2 | 2 | 1 | 1 |  | 0 | 0 | 0 | 0 | 0 |
| 43 | CL6830Contig1_463164 | 2 | 1 | 1 | 1 | 1 |  | 2 | 1 | 1 | 0 | 0 |
| 44 | CL7276Contig1_201674 | 0 | 4 | 2 | 1 | 2 |  | 1 | 1 | 1 | 0 | 1 |
| 45 | CL7327Contig1_464071 | 41 | 39 | 22 | 13 | 21 |  | 62 | 42 | 46 | 20 | 3 |
| 46 | CL7462Contig1_115244 | 47 | 67 | 114 | 69 | 80 |  | 131 | 38 | 96 | 98 | 61 |
| 47 | CL830Contig2_440985 | 3,030 | 3,237 | 4,129 | 8,686 | 8,702 |  | 10,389 | 2,214 | 5,018 | 3,316 | 4,339 |
| 48 | CL9633Contig1_292728 | 0 | 0 | 1 | 0 | 0 |  | 0 | 0 | 0 | 1 | 0 |
| 49 | Group1_Unigene_BMK.11707_529992 | 36 | 28 | 73 | 85 | 104 |  | 110 | 64 | 115 | 95 | 175 |
| 50 | Group1_Unigene_BMK.11707_529997 | 36 | 28 | 73 | 85 | 104 |  | 112 | 65 | 117 | 95 | 175 |
| 51 | Group1_Unigene_BMK.13350_537542 | 22 | 30 | 29 | 35 | 29 |  | 31 | 24 | 57 | 26 | 17 |
| 52 | Group1_Unigene_BMK.14512_543060 | 0 | 0 | 1 | 0 | 2 |  | 0 | 0 | 2 | 0 | 2 |
| 53 | Group1_Unigene_BMK.15584_548367 | 0 | 2 | 0 | 0 | 0 |  | 4 | 0 | 0 | 0 | 1 |
| 54 | Group1_Unigene_BMK.15797_549272 | 177 | 132 | 438 | 361 | 353 |  | 393 | 264 | 365 | 369 | 531 |
| 55 | Group1_Unigene_BMK.16438_552036 | 0 | 1 | 0 | 0 | 0 |  | 1 | 0 | 0 | 2 | 1 |
| 56 | Group1_Unigene_BMK.18362_561528 | 0 | 1 | 0 | 0 | 2 |  | 0 | 0 | 0 | 0 | 0 |
| 57 | Group1_Unigene_BMK.19312_566326 | 3 | 4 | 8 | 5 | 5 |  | 5 | 4 | 6 | 3 | 2 |
| 58 | Group1_Unigene_BMK.20515_572808 | 0 | 6 | 0 | 4 | 3 |  | 15 | 24 | 15 | 8 | 7 |
| 59 | Group1_Unigene_BMK.23434_588836 | 20 | 21 | 33 | 25 | 24 |  | 19 | 20 | 15 | 9 | 10 |
| 60 | Group1_Unigene_BMK.28128_617362 | 2 | 1 | 0 | 2 | 0 |  | 1 | 0 | 0 | 1 | 0 |
| 61 | Group1_Unigene_BMK.2988_630525 | 7,209 | 14,268 | 12,463 | 21,865 | 26,435 |  | 21,580 | 12,092 | 18,626 | 16,094 | 15,330 |
| 62 | Group1_Unigene_BMK.3001_631551 | 63 | 99 | 104 | 63 | 114 |  | 83 | 67 | 63 | 65 | 40 |
| 63 | Group1_Unigene_BMK.30453_635412 | 6 | 2 | 0 | 1 | 1 |  | 0 | 0 | 0 | 1 | 0 |
| 64 | Group1_Unigene_BMK.30485_635795 | 0 | 1 | 0 | 0 | 0 |  | 0 | 0 | 0 | 0 | 0 |
| 65 | Group1_Unigene_BMK.35816_681660 | 4 | 0 | 0 | 0 | 0 |  | 0 | 0 | 0 | 0 | 0 |
| 66 | Group1_Unigene_BMK.36331_686670 | 0 | 0 | 0 | 1 | 0 |  | 0 | 2 | 0 | 1 | 0 |
| 67 | Group1_Unigene_BMK.37364_696840 | 0 | 0 | 0 | 0 | 0 |  | 1 | 2 | 0 | 0 | 0 |
| 68 | Group1_Unigene_BMK.37987_703484 | 302 | 563 | 747 | 807 | 602 |  | 650 | 280 | 434 | 325 | 359 |
| 69 | Group1_Unigene_BMK.38037_703962 | 0 | 0 | 0 | 0 | 0 |  | 0 | 0 | 0 | 0 | 1 |
| 70 | Group1_Unigene_BMK.38221_705979 | 26 | 54 | 65 | 85 | 74 |  | 71 | 50 | 62 | 32 | 40 |
| 71 | Group1_Unigene_BMK.39624_721083 | 0 | 0 | 0 | 0 | 2 |  | 0 | 0 | 0 | 0 | 0 |
| 72 | Group1_Unigene_BMK.39687_721930 | 0 | 0 | 0 | 2 | 0 |  | 0 | 0 | 0 | 0 | 0 |
| 73 | Group1_Unigene_BMK.41654_746253 | 3 | 5 | 3 | 1 | 3 |  | 1 | 0 | 0 | 0 | 1 |
| 74 | Group1_Unigene_BMK.42489_757217 | 179 | 154 | 160 | 264 | 176 |  | 196 | 79 | 171 | 99 | 183 |
| 75 | Group1_Unigene_BMK.43619_773032 | 70 | 60 | 93 | 91 | 113 |  | 84 | 39 | 43 | 98 | 38 |
| 76 | Group1_Unigene_BMK.43625_773061 | 0 | 0 | 2 | 1 | 0 |  | 0 | 0 | 0 | 0 | 0 |
| 77 | Group1_Unigene_BMK.44055_779389 | 22 | 62 | 20 | 47 | 29 |  | 19 | 47 | 57 | 32 | 33 |
| 78 | Group1_Unigene_BMK.44889_790637 | 2 | 1 | 1 | 1 | 0 |  | 0 | 0 | 0 | 0 | 0 |
| 79 | Group1_Unigene_BMK.4944_845482 | 25 | 64 | 32 | 39 | 38 |  | 31 | 51 | 24 | 12 | 17 |
| 80 | Group1_Unigene_BMK.53795_860370 | 0 | 0 | 1 | 0 | 0 |  | 1 | 1 | 0 | 0 | 1 |
| 81 | Group1_Unigene_BMK.61847_888828 | 0 | 0 | 0 | 0 | 0 |  | 0 | 1 | 2 | 0 | 0 |
| 82 | Group1_Unigene_BMK.62619_891469 | 4 | 4 | 4 | 2 | 8 |  | 8 | 1 | 4 | 3 | 2 |
| 83 | Group1_Unigene_BMK.65220_901312 | 7 | 10 | 9 | 5 | 5 |  | 7 | 7 | 6 | 4 | 2 |
| 84 | Group1_Unigene_BMK.6670_907230 | 7,066 | 7,676 | 12,080 | 26,220 | 17,875 |  | 46,732 | 5,718 | 14,572 | 13,438 | 22,248 |
| 85 | Group1_Unigene_BMK.68667_914583 | 0 | 0 | 0 | 0 | 0 |  | 3 | 0 | 0 | 0 | 0 |
| 86 | Group1_Unigene_BMK.68908_915592 | 1 | 0 | 0 | 0 | 0 |  | 0 | 0 | 1 | 0 | 0 |
| 87 | Group1_Unigene_BMK.69336_917544 | 0 | 0 | 0 | 0 | 0 |  | 0 | 0 | 2 | 0 | 0 |
| 88 | Group1_Unigene_BMK.70411_921527 | 0 | 0 | 0 | 0 | 0 |  | 0 | 2 | 0 | 0 | 0 |
| 89 | Group1_Unigene_BMK.71687_926459 | 3 | 1 | 1 | 3 | 6 |  | 1 | 0 | 2 | 27 | 1 |
| 90 | Group1_Unigene_BMK.7537_936473 | 6 | 3 | 1 | 4 | 4 |  | 4 | 0 | 0 | 4 | 6 |
| 91 | Group1_Unigene_BMK.8250_939783 | 0 | 0 | 1 | 0 | 0 |  | 0 | 0 | 0 | 1 | 0 |
| 92 | Group2_Unigene_BMK.10496_950042 | 2 | 0 | 1 | 0 | 0 |  | 0 | 0 | 2 | 0 | 0 |
| 93 | Group2_Unigene_BMK.11710_955285 | 0 | 1 | 1 | 0 | 0 |  | 0 | 0 | 0 | 0 | 0 |
| 94 | Group2_Unigene_BMK.11969_956441 | 0 | 2 | 2 | 0 | 0 |  | 2 | 1 | 1 | 0 | 0 |
| 95 | Group2_Unigene_BMK.13012_961462 | 11 | 18 | 10 | 11 | 18 |  | 15 | 6 | 12 | 7 | 10 |
| 96 | Group2_Unigene_BMK.14792_969121 | 5 | 4 | 6 | 2 | 8 |  | 2 | 2 | 2 | 0 | 0 |
| 97 | Group2_Unigene_BMK.14792_969122 | 5 | 4 | 6 | 2 | 8 |  | 2 | 2 | 2 | 0 | 0 |
| 98 | Group2_Unigene_BMK.15411_971925 | 0 | 0 | 0 | 0 | 0 |  | 0 | 0 | 0 | 1 | 1 |
| 99 | Group2_Unigene_BMK.18440_986990 | 0 | 0 | 0 | 0 | 0 |  | 0 | 2 | 0 | 0 | 0 |
| 100 | Group2_Unigene_BMK.22100_1005948 | 781 | 1,558 | 1,377 | 2,167 | 1,623 |  | 5,523 | 1,955 | 3,640 | 2,874 | 3,674 |
| 101 | Group2_Unigene_BMK.24954_1023233 | 3,053 | 5,120 | 4,056 | 6,493 | 4,349 |  | 2,940 | 3,081 | 3,408 | 2,509 | 2,486 |
| 102 | Group2_Unigene_BMK.25157_1024818 | 36 | 28 | 73 | 85 | 104 |  | 110 | 64 | 115 | 95 | 175 |
| 103 | Group2_Unigene_BMK.25157_1024821 | 36 | 28 | 73 | 85 | 104 |  | 112 | 65 | 117 | 95 | 175 |
| 104 | Group2_Unigene_BMK.25259_1025465 | 302 | 563 | 747 | 807 | 602 |  | 650 | 280 | 434 | 325 | 359 |
| 105 | Group2_Unigene_BMK.25259_1025498 | 302 | 563 | 747 | 807 | 602 |  | 650 | 280 | 434 | 325 | 359 |
| 106 | Group2_Unigene_BMK.2550_1027137 | 0 | 1 | 2 | 5 | 0 |  | 2 | 0 | 1 | 2 | 1 |
| 107 | Group2_Unigene_BMK.26971_1036600 | 0 | 1 | 0 | 0 | 0 |  | 0 | 0 | 3 | 0 | 0 |
| 108 | Group2_Unigene_BMK.31029_1065059 | 10,283 | 11,269 | 16,998 | 37,052 | 26,522 |  | 61,649 | 9,194 | 22,808 | 20,517 | 29,634 |
| 109 | Group2_Unigene_BMK.32903_1080416 | 2 | 7 | 2 | 1 | 4 |  | 8 | 1 | 0 | 4 | 0 |
| 110 | Group2_Unigene_BMK.34335_1093229 | 20 | 21 | 33 | 25 | 24 |  | 19 | 20 | 15 | 9 | 10 |
| 111 | Group2_Unigene_BMK.35270_1102408 | 0 | 0 | 0 | 2 | 0 |  | 0 | 0 | 0 | 0 | 0 |
| 112 | Group2_Unigene_BMK.35892_1108432 | 8 | 8 | 4 | 2 | 6 |  | 12 | 6 | 3 | 5 | 1 |
| 113 | Group2_Unigene_BMK.36958_1119923 | 0 | 0 | 0 | 0 | 0 |  | 0 | 0 | 1 | 0 | 0 |
| 114 | Group2_Unigene_BMK.39451_1148377 | 0 | 0 | 0 | 0 | 0 |  | 1 | 0 | 0 | 0 | 0 |
| 115 | Group2_Unigene_BMK.39605_1150110 | 0 | 1 | 1 | 0 | 0 |  | 2 | 1 | 0 | 1 | 0 |
| 116 | Group2_Unigene_BMK.39959_1154630 | 3 | 5 | 4 | 0 | 1 |  | 3 | 1 | 2 | 2 | 1 |
| 117 | Group2_Unigene_BMK.42016_1180980 | 0 | 0 | 0 | 1 | 0 |  | 0 | 2 | 0 | 1 | 0 |
| 118 | Group2_Unigene_BMK.42740_1191230 | 3 | 4 | 8 | 5 | 5 |  | 5 | 4 | 6 | 3 | 2 |
| 119 | Group2_Unigene_BMK.42927_1193756 | 0 | 1 | 3 | 0 | 1 |  | 0 | 1 | 1 | 0 | 1 |
| 120 | Group2_Unigene_BMK.42987_1194606 | 0 | 2 | 0 | 0 | 0 |  | 0 | 0 | 0 | 0 | 0 |
| 121 | Group2_Unigene_BMK.44131_1210968 | 179 | 154 | 160 | 264 | 176 |  | 196 | 79 | 171 | 99 | 183 |
| 122 | Group2_Unigene_BMK.44348_1214144 | 1 | 0 | 0 | 1 | 1 |  | 2 | 1 | 1 | 1 | 2 |
| 123 | Group2_Unigene_BMK.44717_1219504 | 0 | 1 | 0 | 0 | 1 |  | 0 | 0 | 0 | 1 | 0 |
| 124 | Group2_Unigene_BMK.44748_1220217 | 0 | 0 | 3 | 1 | 0 |  | 0 | 0 | 0 | 0 | 0 |
| 125 | Group2_Unigene_BMK.46880_1254292 | 2 | 2 | 0 | 1 | 0 |  | 0 | 0 | 0 | 1 | 0 |
| 126 | Group2_Unigene_BMK.48663_1261074 | 0 | 2 | 1 | 5 | 0 |  | 4 | 1 | 2 | 1 | 1 |
| 127 | Group2_Unigene_BMK.49487_1263796 | 4,216 | 6,531 | 3,323 | 7,213 | 5,059 |  | 7,347 | 1,882 | 5,122 | 2,688 | 4,015 |
| 128 | Group2_Unigene_BMK.49608_1264296 | 8 | 5 | 4 | 1 | 6 |  | 4 | 3 | 2 | 0 | 2 |
| 129 | Group2_Unigene_BMK.49791_1264903 | 0 | 0 | 0 | 0 | 0 |  | 0 | 2 | 0 | 0 | 0 |
| 130 | Group2_Unigene_BMK.5048_1267308 | 0 | 0 | 0 | 0 | 0 |  | 1 | 0 | 0 | 0 | 1 |
| 131 | Group2_Unigene_BMK.50808_1268477 | 0 | 0 | 0 | 1 | 0 |  | 1 | 0 | 0 | 1 | 0 |
| 132 | Group2_Unigene_BMK.56446_1288539 | 0 | 0 | 0 | 0 | 0 |  | 0 | 1 | 0 | 0 | 0 |
| 133 | Group2_Unigene_BMK.56446_1288542 | 0 | 0 | 0 | 0 | 0 |  | 0 | 1 | 0 | 0 | 0 |
| 134 | Group2_Unigene_BMK.58781_1297340 | 121 | 151 | 94 | 150 | 98 |  | 218 | 97 | 149 | 85 | 125 |
| 135 | Group2_Unigene_BMK.5977_1300964 | 0 | 0 | 1 | 0 | 0 |  | 0 | 0 | 0 | 1 | 0 |
| 136 | Group2_Unigene_BMK.60353_1303142 | 1 | 0 | 0 | 0 | 0 |  | 0 | 0 | 0 | 0 | 1 |
| 137 | Group2_Unigene_BMK.60663_1304387 | 0 | 1 | 0 | 0 | 0 |  | 2 | 1 | 2 | 1 | 2 |
| 138 | Group2_Unigene_BMK.62572_1311447 | 0 | 1 | 3 | 1 | 1 |  | 0 | 0 | 0 | 0 | 0 |
| 139 | Group2_Unigene_BMK.64130_1317662 | 0 | 0 | 0 | 1 | 0 |  | 0 | 0 | 0 | 2 | 3 |
| 140 | Group2_Unigene_BMK.66156_1325568 | 0 | 0 | 1 | 0 | 2 |  | 0 | 0 | 2 | 0 | 2 |
| 141 | Group2_Unigene_BMK.68987_1337794 | 0 | 0 | 0 | 0 | 0 |  | 0 | 0 | 0 | 3 | 0 |
| 142 | Group2_Unigene_BMK.71224_1347474 | 23 | 46 | 89 | 90 | 168 |  | 129 | 74 | 41 | 90 | 47 |
| 143 | Group2_Unigene_BMK.71441_1348388 | 1 | 0 | 1 | 4 | 1 |  | 0 | 0 | 0 | 1 | 0 |
| 144 | Group2_Unigene_BMK.71697_1349603 | 0 | 1 | 0 | 0 | 0 |  | 0 | 0 | 1 | 0 | 0 |
| 145 | Group2_Unigene_BMK.71973_1350885 | 0 | 0 | 0 | 1 | 1 |  | 0 | 0 | 0 | 0 | 0 |
| 146 | Group2_Unigene_BMK.72865_1354993 | 0 | 5 | 0 | 1 | 1 |  | 0 | 0 | 0 | 1 | 0 |
| 147 | Group2_Unigene_BMK.7290_1355121 | 33 | 9 | 7 | 86 | 17 |  | 5 | 0 | 1 | 6 | 6 |
| 148 | Group2_Unigene_BMK.74236_1360820 | 0 | 0 | 1 | 1 | 2 |  | 3 | 0 | 1 | 1 | 0 |
| 149 | Group2_Unigene_BMK.74496_1361897 | 0 | 0 | 0 | 0 | 0 |  | 0 | 1 | 1 | 0 | 0 |
| 150 | Group2_Unigene_BMK.8370_1373185 | 0 | 2 | 0 | 0 | 0 |  | 0 | 0 | 0 | 0 | 0 |
| 151 | Group2_Unigene_BMK.9543_1378570 | 15 | 22 | 11 | 26 | 21 |  | 17 | 10 | 15 | 11 | 7 |
| 152 | CL1154Contig1_268165 | 4 | 4 | 0 | 5 | 7 |  | 3 | 2 | 2 | 3 | 6 |
| 153 | CL12087Contig1_125050 | 0 | 1 | 1 | 4 | 0 |  | 28 | 15 | 14 | 10 | 12 |
| 154 | CL12431Contig1_298491 | 3 | 6 | 2 | 2 | 6 |  | 6 | 2 | 2 | 4 | 0 |
| 155 | CL14060Contig1_389564 | 215 | 250 | 104 | 128 | 239 |  | 196 | 164 | 91 | 44 | 46 |
| 156 | CL1468Contig1_9115 | 0 | 0 | 0 | 0 | 0 |  | 0 | 1 | 0 | 0 | 0 |
| 157 | CL15915Contig1_306240 | 5 | 6 | 2 | 9 | 4 |  | 17 | 5 | 10 | 5 | 5 |
| 158 | CL16456Contig1_483269 | 3 | 0 | 1 | 3 | 1 |  | 2 | 1 | 2 | 1 | 0 |
| 159 | CL16983Contig1_484318 | 69 | 50 | 54 | 85 | 51 |  | 69 | 39 | 51 | 47 | 23 |
| 160 | CL17435Contig1_485238 | 0 | 1 | 1 | 1 | 0 |  | 0 | 1 | 0 | 0 | 0 |
| 161 | CL18311Contig1_311011 | 15 | 11 | 11 | 23 | 23 |  | 25 | 21 | 26 | 37 | 17 |
| 162 | CL1888Contig1_271219 | 526 | 773 | 886 | 1,079 | 987 |  | 1,362 | 920 | 1,213 | 1,065 | 879 |
| 163 | CL19455Contig1_54014 | 26 | 26 | 23 | 22 | 27 |  | 40 | 18 | 28 | 25 | 14 |
| 164 | CL19506Contig1_489507 | 0 | 0 | 0 | 1 | 0 |  | 1 | 1 | 1 | 0 | 2 |
| 165 | CL19777Contig1_314088 | 23 | 34 | 30 | 29 | 26 |  | 35 | 18 | 31 | 26 | 20 |
| 166 | CL21052Contig1_317401 | 51 | 73 | 45 | 62 | 37 |  | 59 | 52 | 94 | 67 | 34 |
| 167 | CL21760Contig1_495493 | 8 | 5 | 0 | 5 | 1 |  | 12 | 4 | 10 | 3 | 1 |
| 168 | CL22840Contig1_235405 | 1 | 0 | 2 | 0 | 0 |  | 2 | 0 | 0 | 3 | 1 |
| 169 | CL23127Contig1_498764 | 0 | 0 | 1 | 0 | 0 |  | 1 | 0 | 0 | 0 | 0 |
| 170 | CL23559Contig1_411515 | 0 | 0 | 0 | 2 | 2 |  | 2 | 1 | 0 | 0 | 0 |
| 171 | CL23878Contig1_325691 | 7 | 10 | 20 | 12 | 15 |  | 20 | 21 | 16 | 13 | 11 |
| 172 | CL24251Contig1_502434 | 1 | 2 | 0 | 4 | 3 |  | 4 | 1 | 2 | 0 | 0 |
| 173 | CL2440Contig1_359627 | 2 | 6 | 13 | 5 | 4 |  | 5 | 1 | 5 | 3 | 4 |
| 174 | CL25536Contig1_330427 | 3 | 4 | 2 | 5 | 1 |  | 1 | 0 | 2 | 0 | 1 |
| 175 | CL26040Contig1_71826 | 3 | 1 | 0 | 1 | 0 |  | 1 | 5 | 0 | 1 | 0 |
| 176 | CL26801Contig1_333530 | 0 | 0 | 8 | 7 | 2 |  | 1 | 2 | 2 | 3 | 1 |
| 177 | CL26875Contig1_159660 | 14 | 28 | 25 | 10 | 18 |  | 21 | 8 | 13 | 20 | 16 |
| 178 | CL2738Contig1_14043 | 2 | 6 | 1 | 3 | 7 |  | 7 | 10 | 10 | 6 | 4 |
| 179 | CL28094Contig1_336251 | 0 | 1 | 1 | 0 | 0 |  | 4 | 3 | 2 | 1 | 3 |
| 180 | CL28135Contig1_76088 | 1 | 2 | 0 | 2 | 0 |  | 1 | 0 | 3 | 0 | 1 |
| 181 | CL28290Contig1_424336 | 1 | 0 | 0 | 0 | 0 |  | 0 | 0 | 0 | 0 | 1 |
| 182 | CL2938Contig1_449792 | 20 | 28 | 22 | 39 | 16 |  | 41 | 18 | 35 | 28 | 24 |
| 183 | CL2942Contig1_14758 | 1 | 5 | 4 | 1 | 6 |  | 5 | 1 | 0 | 0 | 2 |
| 184 | CL296Contig1_437746 | 8 | 5 | 18 | 72 | 299 |  | 63 | 1 | 12 | 23 | 20 |
| 185 | CL30127Contig1_80060 | 0 | 0 | 0 | 0 | 0 |  | 0 | 0 | 0 | 1 | 0 |
| 186 | CL30170Contig1_254198 | 1 | 1 | 1 | 1 | 0 |  | 1 | 1 | 3 | 0 | 1 |
| 187 | CL4378Contig1_191450 | 11 | 8 | 10 | 14 | 15 |  | 35 | 13 | 22 | 17 | 12 |
| 188 | CL5007Contig1_279728 | 2 | 4 | 3 | 1 | 2 |  | 3 | 2 | 1 | 2 | 1 |
| 189 | CL8605Contig1_290728 | 0 | 2 | 2 | 1 | 4 |  | 3 | 1 | 0 | 2 | 2 |
| 190 | CL8820Contig1_466849 | 3 | 4 | 4 | 4 | 0 |  | 3 | 3 | 6 | 4 | 3 |
| 191 | CL948Contig1_6755 | 0 | 5 | 0 | 2 | 1 |  | 0 | 1 | 0 | 0 | 0 |
| 192 | CL960Contig1_353479 | 0 | 1 | 0 | 1 | 1 |  | 1 | 0 | 0 | 0 | 2 |
| 193 | CL9644Contig1_380257 | 26 | 24 | 21 | 21 | 18 |  | 20 | 12 | 18 | 10 | 24 |
| 194 | Group1_Unigene_BMK.12513_533812 | 12 | 13 | 12 | 5 | 19 |  | 18 | 2 | 12 | 5 | 9 |
| 195 | Group1_Unigene_BMK.14663_543730 | 0 | 0 | 0 | 0 | 0 |  | 0 | 1 | 0 | 1 | 2 |
| 196 | Group1_Unigene_BMK.16376_551753 | 4 | 7 | 4 | 8 | 4 |  | 8 | 3 | 4 | 4 | 5 |
| 197 | Group1_Unigene_BMK.2067_573693 | 4 | 1 | 4 | 3 | 5 |  | 0 | 1 | 1 | 1 | 1 |
| 198 | Group1_Unigene_BMK.21471_577819 | 4 | 17 | 5 | 21 | 34 |  | 26 | 5 | 7 | 11 | 2 |
| 199 | Group1_Unigene_BMK.21704_578939 | 1 | 2 | 4 | 1 | 0 |  | 2 | 1 | 1 | 0 | 0 |
| 200 | Group1_Unigene_BMK.23831_591394 | 0 | 2 | 3 | 5 | 3 |  | 3 | 0 | 1 | 3 | 0 |
| 201 | Group1_Unigene_BMK.24633_595825 | 646 | 808 | 926 | 895 | 826 |  | 1,988 | 413 | 767 | 792 | 478 |
| 202 | Group1_Unigene_BMK.25646_601906 | 14 | 9 | 8 | 25 | 26 |  | 22 | 7 | 23 | 19 | 1 |
| 203 | Group1_Unigene_BMK.28669_621396 | 1 | 2 | 8 | 0 | 2 |  | 2 | 2 | 1 | 4 | 0 |
| 204 | Group1_Unigene_BMK.29062_624230 | 13 | 17 | 8 | 14 | 11 |  | 17 | 5 | 5 | 9 | 2 |
| 205 | Group1_Unigene_BMK.31136_640883 | 2,045 | 3,415 | 3,020 | 3,571 | 3,794 |  | 4,265 | 2,901 | 4,112 | 3,701 | 2,653 |
| 206 | Group1_Unigene_BMK.34430_668680 | 0 | 1 | 0 | 0 | 0 |  | 2 | 0 | 0 | 0 | 0 |
| 207 | Group1_Unigene_BMK.34588_670216 | 0 | 1 | 1 | 1 | 0 |  | 0 | 1 | 0 | 0 | 0 |
| 208 | Group1_Unigene_BMK.35266_676587 | 81 | 114 | 82 | 109 | 125 |  | 203 | 34 | 69 | 100 | 219 |
| 209 | Group1_Unigene_BMK.35465_678535 | 1,728 | 823 | 981 | 686 | 1,028 |  | 715 | 671 | 1,211 | 937 | 293 |
| 210 | Group1_Unigene_BMK.36099_684338 | 0 | 4 | 0 | 2 | 0 |  | 40 | 27 | 24 | 5 | 65 |
| 211 | Group1_Unigene_BMK.36375_687138 | 1 | 1 | 0 | 0 | 0 |  | 2 | 0 | 0 | 0 | 0 |
| 212 | Group1_Unigene_BMK.37183_695405 | 1 | 6 | 5 | 14 | 5 |  | 4 | 7 | 2 | 2 | 3 |
| 213 | Group1_Unigene_BMK.37987_703482 | 463 | 799 | 740 | 1,129 | 983 |  | 1,411 | 535 | 937 | 821 | 484 |
| 214 | Group1_Unigene_BMK.39098_715925 | 0 | 0 | 1 | 0 | 1 |  | 0 | 0 | 0 | 0 | 0 |
| 215 | Group1_Unigene_BMK.4105_738225 | 2 | 0 | 1 | 2 | 2 |  | 1 | 3 | 3 | 1 | 0 |
| 216 | Group1_Unigene_BMK.42407_756074 | 14 | 36 | 15 | 21 | 20 |  | 16 | 13 | 9 | 15 | 13 |
| 217 | Group1_Unigene_BMK.44414_784344 | 3 | 3 | 0 | 0 | 0 |  | 5 | 0 | 1 | 2 | 0 |
| 218 | Group1_Unigene_BMK.45108_793960 | 0 | 0 | 0 | 0 | 0 |  | 1 | 1 | 0 | 0 | 0 |
| 219 | Group1_Unigene_BMK.45124_794127 | 2 | 2 | 0 | 0 | 0 |  | 1 | 1 | 4 | 1 | 3 |
| 220 | Group1_Unigene_BMK.45675_802511 | 4 | 4 | 8 | 1 | 10 |  | 9 | 4 | 4 | 5 | 3 |
| 221 | Group1_Unigene_BMK.46148_809785 | 1 | 0 | 2 | 0 | 0 |  | 2 | 0 | 0 | 3 | 1 |
| 222 | Group1_Unigene_BMK.49067_844300 | 0 | 4 | 2 | 0 | 1 |  | 2 | 1 | 4 | 1 | 1 |
| 223 | Group1_Unigene_BMK.52032_854311 | 3 | 5 | 3 | 1 | 5 |  | 12 | 5 | 2 | 3 | 10 |
| 224 | Group1_Unigene_BMK.60465_884171 | 1 | 5 | 6 | 0 | 3 |  | 2 | 0 | 1 | 0 | 6 |
| 225 | Group1_Unigene_BMK.61925_889138 | 1 | 1 | 1 | 3 | 1 |  | 1 | 1 | 1 | 0 | 0 |
| 226 | Group1_Unigene_BMK.62191_890098 | 2 | 1 | 4 | 1 | 1 |  | 0 | 0 | 1 | 0 | 2 |
| 227 | Group1_Unigene_BMK.68128_912297 | 0 | 0 | 0 | 0 | 0 |  | 1 | 1 | 5 | 1 | 1 |
| 228 | Group1_Unigene_BMK.69742_918987 | 0 | 1 | 3 | 1 | 5 |  | 0 | 2 | 1 | 4 | 0 |
| 229 | Group1_Unigene_BMK.73698_934628 | 2 | 4 | 1 | 0 | 1 |  | 2 | 3 | 1 | 1 | 3 |
| 230 | Group1_Unigene_BMK.744_936047 | 30 | 70 | 40 | 66 | 65 |  | 55 | 80 | 70 | 49 | 37 |
| 231 | Group2_Unigene_BMK.10601_950447 | 2 | 1 | 1 | 0 | 1 |  | 0 | 1 | 0 | 1 | 1 |
| 232 | Group2_Unigene_BMK.12810_960476 | 0 | 3 | 5 | 0 | 1 |  | 3 | 0 | 0 | 1 | 1 |
| 233 | Group2_Unigene_BMK.14145_966151 | 2 | 1 | 2 | 3 | 4 |  | 0 | 0 | 1 | 1 | 1 |
| 234 | Group2_Unigene_BMK.17316_981512 | 9 | 16 | 12 | 8 | 5 |  | 9 | 18 | 17 | 15 | 14 |
| 235 | Group2_Unigene_BMK.18125_985418 | 12 | 35 | 61 | 12 | 11 |  | 5 | 3 | 10 | 142 | 9 |
| 236 | Group2_Unigene_BMK.20139_995743 | 2 | 2 | 1 | 3 | 1 |  | 1 | 0 | 1 | 1 | 1 |
| 237 | Group2_Unigene_BMK.23778_1015561 | 5 | 15 | 9 | 19 | 11 |  | 34 | 4 | 8 | 14 | 12 |
| 238 | Group2_Unigene_BMK.23997_1016824 | 2 | 3 | 4 | 1 | 2 |  | 0 | 1 | 1 | 2 | 1 |
| 239 | Group2_Unigene_BMK.24252_1018543 | 1 | 1 | 1 | 0 | 0 |  | 1 | 2 | 2 | 1 | 0 |
| 240 | Group2_Unigene_BMK.24449_1019737 | 79 | 71 | 145 | 160 | 332 |  | 130 | 115 | 141 | 235 | 130 |
| 241 | Group2_Unigene_BMK.25259_1025467 | 463 | 799 | 740 | 1,129 | 983 |  | 1,411 | 535 | 937 | 821 | 484 |
| 242 | Group2_Unigene_BMK.25259_1025496 | 463 | 799 | 740 | 1,129 | 983 |  | 1,411 | 535 | 937 | 821 | 484 |
| 243 | Group2_Unigene_BMK.28329_1045828 | 0 | 0 | 0 | 0 | 0 |  | 1 | 0 | 0 | 1 | 0 |
| 244 | Group2_Unigene_BMK.29302_1052092 | 181 | 223 | 272 | 352 | 297 |  | 261 | 144 | 165 | 115 | 154 |
| 245 | Group2_Unigene_BMK.30181_1058640 | 2 | 0 | 3 | 0 | 2 |  | 0 | 1 | 0 | 4 | 0 |
| 246 | Group2_Unigene_BMK.30664_1062471 | 386 | 771 | 523 | 896 | 788 |  | 711 | 941 | 603 | 421 | 344 |
| 247 | Group2_Unigene_BMK.30670_1062502 | 0 | 1 | 0 | 0 | 0 |  | 2 | 0 | 0 | 0 | 0 |
| 248 | Group2_Unigene_BMK.31794_1071090 | 2,045 | 3,415 | 3,020 | 3,571 | 3,794 |  | 4,265 | 2,901 | 4,112 | 3,701 | 2,653 |
| 249 | Group2_Unigene_BMK.33056_1081836 | 26 | 22 | 9 | 39 | 15 |  | 279 | 40 | 27 | 131 | 48 |
| 250 | Group2_Unigene_BMK.34396_1093900 | 0 | 2 | 3 | 5 | 3 |  | 3 | 0 | 1 | 3 | 0 |
| 251 | Group2_Unigene_BMK.35753_1107175 | 0 | 1 | 0 | 0 | 0 |  | 0 | 2 | 2 | 1 | 0 |
| 252 | Group2_Unigene_BMK.35875_1108373 | 0 | 0 | 0 | 1 | 0 |  | 5 | 27 | 24 | 13 | 0 |
| 253 | Group2_Unigene_BMK.36747_1117795 | 1 | 2 | 1 | 5 | 1 |  | 2 | 2 | 3 | 2 | 4 |
| 254 | Group2_Unigene_BMK.38504_1137258 | 35 | 31 | 29 | 40 | 33 |  | 45 | 25 | 24 | 13 | 25 |
| 255 | Group2_Unigene_BMK.38504_1137263 | 6 | 3 | 4 | 3 | 4 |  | 2 | 0 | 2 | 2 | 1 |
| 256 | Group2_Unigene_BMK.39199_1145135 | 1 | 2 | 0 | 0 | 0 |  | 2 | 0 | 0 | 1 | 1 |
| 257 | Group2_Unigene_BMK.41943_1179945 | 0 | 0 | 1 | 0 | 0 |  | 1 | 0 | 0 | 0 | 0 |
| 258 | Group2_Unigene_BMK.42599_1188665 | 0 | 2 | 1 | 0 | 1 |  | 4 | 0 | 1 | 1 | 1 |
| 259 | Group2_Unigene_BMK.44346_1214103 | 7 | 26 | 9 | 8 | 17 |  | 12 | 8 | 7 | 6 | 4 |
| 260 | Group2_Unigene_BMK.45260_1228779 | 0 | 2 | 1 | 2 | 1 |  | 4 | 1 | 3 | 1 | 0 |
| 261 | Group2_Unigene_BMK.48938_1261992 | 0 | 0 | 1 | 0 | 0 |  | 0 | 0 | 0 | 1 | 0 |
| 262 | Group2_Unigene_BMK.5248_1274425 | 39 | 22 | 44 | 79 | 82 |  | 116 | 45 | 116 | 84 | 134 |
| 263 | Group2_Unigene_BMK.5286_1275805 | 10 | 14 | 14 | 8 | 21 |  | 8 | 15 | 7 | 8 | 5 |
| 264 | Group2_Unigene_BMK.52890_1275908 | 4 | 7 | 10 | 7 | 1 |  | 5 | 0 | 4 | 2 | 2 |
| 265 | Group2_Unigene_BMK.53901_1279310 | 5 | 10 | 7 | 4 | 1 |  | 3 | 0 | 3 | 0 | 5 |
| 266 | Group2_Unigene_BMK.58728_1297086 | 702 | 996 | 595 | 544 | 393 |  | 939 | 481 | 588 | 413 | 267 |
| 267 | Group2_Unigene_BMK.58814_1297424 | 2 | 2 | 1 | 2 | 3 |  | 0 | 0 | 0 | 0 | 0 |
| 268 | Group2_Unigene_BMK.60122_1302355 | 2,036 | 2,839 | 2,012 | 2,132 | 2,969 |  | 2,368 | 1,819 | 1,865 | 1,639 | 943 |
| 269 | Group2_Unigene_BMK.63506_1315063 | 58 | 47 | 89 | 49 | 53 |  | 96 | 49 | 34 | 50 | 20 |
| 270 | Group2_Unigene_BMK.66230_1325887 | 0 | 3 | 1 | 2 | 2 |  | 0 | 1 | 0 | 1 | 0 |
| 271 | Group2_Unigene_BMK.7016_1342668 | 9 | 9 | 9 | 4 | 9 |  | 9 | 5 | 6 | 3 | 3 |
| 272 | Group2_Unigene_BMK.74684_1362690 | 0 | 0 | 0 | 0 | 0 |  | 0 | 0 | 0 | 0 | 1 |
| 273 | Group2_Unigene_BMK.75_1363922 | 3 | 3 | 1 | 4 | 5 |  | 11 | 4 | 2 | 1 | 1 |
| 274 | Group2_Unigene_BMK.983_1379933 | 646 | 808 | 926 | 895 | 826 |  | 1,988 | 413 | 767 | 792 | 478 |

| Table S5. All conservative miRNA discovered in the two camellia species during seed natural drying | | | | | | | | |
| --- | --- | --- | --- | --- | --- | --- | --- | --- |
| **Number** | **Pre**-**miRNA** | **Mature miRNA** | **miRNA family** | **Mature sequence** | **Length (nt)** | **LP(nt)** | **Score total** | **Total read count** |
| 1 | Group1_Unigene_BMK.2988_630525 | ath-miR165a-3p | MIR166 | ucggaccaggcuucauucccc | 21 | 108 | 78775.9 | 154506 |
| 2 | CL24215Contig1_413571 | ath-miR156a-5p |  | ugacagaagagagugagcac | 20 | 110 | 125258.5 | 245680 |
| 3 | Group1_Unigene_BMK.6670_907230 | ath-miR156a-5p |  | ugacagaagagagugagcac | 20 | 108 | 91341.5 | 179153 |
| 4 | CL19817Contig1_140074 | ath-miR156a-5p |  | ugacagaagagagugagcac | 20 | 108 | 90709.6 | 177914 |
| 5 | CL21369Contig1_405539 | ath-miR156a-5p |  | ugacagaagagagugagcac | 20 | 110 | 127144.0 | 249378 |
| 6 | Group2_Unigene_BMK.31029_1065059 | ath-miR156a-5p |  | ugacagaagagagugagcac | 20 | 110 | 127144.0 | 249378 |
| 7 | CL2190Contig1_98714 | ath-miR162a-3p | MIR162_2 | ucgauaaaccucugcauccag | 21 | 111 | 59792.1 | 117272 |
| 8 | Group2_Unigene_BMK.24954_1023233 | ath-miR3440b-5p |  | auuucuugguugcuuuacucc | 21 | 111 | 19249.1 | 37748 |
| 9 | CL830Contig2_440985 | ath-miR157a-5p |  | uugacagaagagagagagcac | 21 | 111 | 30866.5 | 60534 |
| 10 | Group2_Unigene_BMK.49487_1263796 | ath-miR396a-5p | MIR396 | uuccacagcuuucuugaacuu | 21 | 111 | 25254.0 | 49526 |
| 11 | CL22098Contig1_407310 | gra-miR7486i | MIR1878 | acugacguguucaacuugaauugg | 24 | 114 | 8443.7 | 16552 |
| 12 | CL17638Contig1_50456 | vvi-miR3631b-3p |  | uguuggaugaaucguugucag | 21 | 109 | 0.7 | 8920 |
| 13 | Group1_Unigene_BMK.37987_703484 | osa-miR2118e | MIR2118 | uucccaaucccucccauuccua | 22 | 112 | 2869.1 | 5619 |
| 14 | Group2_Unigene_BMK.25259_1025498 | osa-miR2118e | MIR482 | uucccaaucccucccauuccua | 22 | 112 | 2869.1 | 5619 |
| 15 | Group2_Unigene_BMK.25259_1025465 | osa-miR2118e | MIR482 | uucccaaucccucccauuccua | 22 | 111 | 2868.9 | 5619 |
| 16 | CL22574Contig1_61569 | ath-miR167a-5p | MIR167_1 | ugaagcugccagcaugaucua | 21 | 112 | 8417.4 | 16501 |
| 17 | Group2_Unigene_BMK.22100_1005948 | ath-miR167a-5p | MIR167_1 | ugaagcugccagcaugaucuga | 22 | 112 | 13003.2 | 25496 |
| 18 | CL29290Contig1_252046 | osa-miR1859 | MIR482 | uuuccuauuccucccauaccua | 22 | 112 | 5.5 | 1921 |
| 19 | Group2_Unigene_BMK.44131_1210968 | sbi-miR6232a-3p |  | uggauguagcaaagagaagcu | 21 | 111 | 879.9 | 1716 |
| 20 | Group1_Unigene_BMK.42489_757217 | sbi-miR6232a-3p |  | uggauguagcaaagagaagcu | 21 | 110 | 879.9 | 1716 |
| 21 | CL7462Contig1_115244 | ath-miR394a | MIR394 | uuggcauucuguccaccucc | 20 | 110 | 4.5 | 798 |
| 22 | Group2_Unigene_BMK.58781_1297340 | ath-miR171b-3p | MIR171_1 | uugagccgcgucaauaucucu | 21 | 110 | 4.7 | 1290 |
| 23 | Group1_Unigene_BMK.11707_529997 | ppt-miR535a | MIR535 | ugacaacgagagagagcacgc | 21 | 111 | 509.2 | 989 |
| 24 | Group2_Unigene_BMK.25157_1024821 | ppt-miR535a | MIR535 | ugacaacgagagagagcacgc | 21 | 111 | 509.2 | 989 |
| 25 | Group1_Unigene_BMK.11707_529992 | ppt-miR535a | MIR535 | ugacaacgagagagagcacgc | 21 | 111 | 459.6 | 892 |
| 26 | Group2_Unigene_BMK.25157_1024818 | ppt-miR535a | MIR535 | ugacaacgagagagagcacgc | 21 | 111 | 459.6 | 892 |
| 27 | Group1_Unigene_BMK.15797_549272 | mtr-miR7701-3p |  | guagguucguuugucguuggggcua | 25 | 108 | 0.5 | 3316 |
| 28 | CL26738Contig1_509111 | ath-miR160a-5p | MIR160 | ugccuggcucccuguaugcca | 21 | 111 | 212.8 | 408 |
| 29 | Group1_Unigene_BMK.38221_705979 | vvi-miR3634-3p | bantam | uuuccgaccuaguuuucaaac | 21 | 111 | 0.3 | 488 |
| 30 | CL18763Contig1_311943 | ptc-miR398c-5p | MIR398 | ggagcgacuugagaucacauc | 21 | 111 | 642.1 | 1250 |
| 31 | CL7327Contig1_464071 | osa-miR167e-3p |  | ugaucauguggcucuggguggcau | 24 | 112 | 175.4 | 335 |
| 32 | Group1_Unigene_BMK.44055_779389 | csi-miR3950 |  | auuuucgggguacuuuuuugu | 21 | 108 | 196.8 | 377 |
| 33 | Group1_Unigene_BMK.4944_845482 | mtr-miR5210 |  | caaaugugacauuucggcgacuaa | 24 | 115 | 201.8 | 388 |
| 34 | Group2_Unigene_BMK.34335_1093229 | osa-miR1861a | MIR5067 | agaucuugcguuggaacuccaagg | 24 | 114 | 4.8 | 211 |
| 35 | Group1_Unigene_BMK.23434_588836 | osa-miR1861a | MIR5067 | agaucuugcguuggaacuccaagg | 24 | 114 | 4.8 | 211 |
| 36 | CL2149Contig1_11711 | ath-miR160a-5p | MIR160 | ugccuggcucccuguaugcc | 20 | 108 | 0.4 | 158 |
| 37 | CL2149Contig1_11712 | ath-miR160a-5p | MIR160 | ugccuggcucccuguaugcc | 20 | 108 | 0.5 | 160 |
| 38 | Group1_Unigene_BMK.65220_901312 | bdi-miR5202 |  | uuacgugauuuugugaaauaacuu | 24 | 114 | 3.9 | 72 |
| 39 | Group2_Unigene_BMK.13012_961462 | ath-miR830-3p | MIR6297 | aaacuauuuuuggucgccgaaagu | 24 | 114 | 3.9 | 76 |
| 40 | CL29078Contig1_514119 | gma-miR5035-3p |  | uguuuagaucuugaauuugug | 21 | 111 | 4.4 | 83 |
| 41 | Group2_Unigene_BMK.7290_1355121 | aqc-miR171f | MIR397 | uaauugagugcagcguugaug | 21 | 106 | 171.6 | 328 |
| 42 | CL17766Contig1_485769 | osa-miR167d-3p |  | caucaugcuuauggauucuacaac | 24 | 114 | 5.5 | 131 |
| 43 | Group1_Unigene_BMK.43619_773032 | bdi-miR5183 |  | gauuuggaaagagaaagggaaagg | 24 | 94 | 110.4 | 208 |
| 44 | Group1_Unigene_BMK.3001_631551 | bdi-miR7761-3p |  | acuugaucaauaggauuauacgag | 24 | 114 | 6.0 | 62 |
| 45 | Group2_Unigene_BMK.42740_1191230 | osa-miR2118a |  | uucucgaucacuucggccaaggag | 24 | 114 | 1.0 | 50 |
| 46 | Group1_Unigene_BMK.19312_566326 | osa-miR2118a |  | uucucgaucacuucggccaaggag | 24 | 114 | 1.0 | 50 |
| 47 | Group2_Unigene_BMK.39959_1154630 | gra-miR8756 | MIR6020 | aggacuguuuuuccgaucaucuuc | 24 | 114 | 4.1 | 22 |
| 48 | CL5425Contig1_367528 | osa-miR5079a |  | uuuggaucucaaccauugauggug | 24 | 114 | 5.9 | 35 |
| 49 | Group1_Unigene_BMK.13350_537542 | osa-miR2095-3p | MIR5185 | uuuccauuuccuuuuccaaauccu | 24 | 95 | 4.9 | 39 |
| 50 | Group2_Unigene_BMK.44748_1220217 | mtr-miR1507-5p |  | cgaguuguggauguucuuaacgug | 24 | 114 | 0.6 | 4 |
| 51 | Group2_Unigene_BMK.62572_1311447 | gma-miR5778 | MIR169_2 | ugacgaacuguuuuugaaaacaac | 24 | 114 | 1.4 | 7 |
| 52 | Group2_Unigene_BMK.9543_1378570 | bdi-miR5185m-3p |  | uuugaaaaggaaaaggaaaaaggu | 24 | 113 | 5.0 | 64 |
| 53 | CL29784Contig1_515758 | bdi-miR7777-5p.1 |  | guuugguugacauaguaaaaugau | 24 | 114 | 4.0 | 61 |
| 54 | Group2_Unigene_BMK.49608_1264296 | tae-miR9674a-5p |  | ucaucaucugugauucuaacgugu | 24 | 114 | 4.3 | 51 |
| 55 | Group1_Unigene_BMK.20515_572808 | mtr-miR5296 |  | uuuuugugggacagcgaauaaagu | 24 | 111 | 82.1 | 152 |
| 56 | Group1_Unigene_BMK.43625_773061 | aly-miR398b-5p |  | ugguugauggaacaucguauuggc | 24 | 114 | 0.8 | 3 |
| 57 | CL29150Contig1_514235 | ptc-miR7815 | MIR396 | aucuucaaucaggcuguagaaguu | 24 | 114 | 4.8 | 17 |
| 58 | Group2_Unigene_BMK.42927_1193756 | tae-miR5086 | MIR1878 | ccauuggucgugucgaaacagagu | 24 | 113 | 10.5 | 13 |
| 59 | Group1_Unigene_BMK.69336_917544 | mtr-miR1510a-5p |  | uugucuuaugugggccugagg | 21 | 114 | 16.1 | 23 |
| 60 | Group2_Unigene_BMK.2550_1027137 | bdi-miR9497 | MIR395 | auucugaacacuuuuuug | 18 | 108 | 4.2 | 12 |
| 61 | Group2_Unigene_BMK.66156_1325568 | osa-miR5081 |  | caauuuguagaacuugaacaugug | 24 | 114 | 3.1 | 10 |
| 62 | Group1_Unigene_BMK.14512_543060 | osa-miR5081 |  | caauuuguagaacuugaacaugug | 24 | 114 | 3.1 | 10 |
| 63 | CL7276Contig1_201674 | mtr-miR7697-3p | MIR829 | gccgaucauuugauucgaacucaa | 24 | 112 | 6.0 | 5 |
| 64 | CL6211Contig1_461735 | osa-miR167d-3p |  | aaucaugccuggaagaacagaggu | 24 | 114 | 1.7 | 6 |
| 65 | CL24280Contig1_502539 | osa-miR5793 |  | agaggacgucuuuggaguggccga | 24 | 114 | 1.9 | 3 |
| 66 | Group2_Unigene_BMK.48663_1261074 | pta-miR159a |  | uuggauuggguuaaagguuaguc | 23 | 113 | 5.9 | 17 |
| 67 | Group1_Unigene_BMK.18362_561528 | osa-miR1880 | MIR5565 | auccaagccuguugauaucucauc | 24 | 114 | 5.5 | 11 |
| 68 | CL22681Contig1_147702 | mtr-miR5224a |  | acgaggacggugcggaccaagguc | 24 | 114 | 0.5 | 2 |
| 69 | Group1_Unigene_BMK.8250_939783 | mtr-miR2587a |  | augaccguccgguuguccuggugc | 24 | 114 | 2.9 | 5 |
| 70 | Group2_Unigene_BMK.14792_969122 | stu-miR8012 | MIR169_2 | gugacuuuuggcuacuaaacaacc | 24 | 113 | 4.8 | 21 |
| 71 | Group2_Unigene_BMK.14792_969121 | stu-miR8012 | MIR169_2 | gugacuuuuggcuacuaaacaacc | 24 | 113 | 3.7 | 22 |
| 72 | CL19980Contig1_228041 | aly-miR167d-3p |  | uggucaucagccggagcugcc | 21 | 111 | 1.0 | 3 |
| 73 | Group1_Unigene_BMK.44889_790637 | ptc-miR7835 |  | aaugggauauagaaucuugacggu | 24 | 114 | 1.5 | 5 |
| 74 | CL19869Contig1_140190 | ptc-miR160e-3p |  | ccaugaggugggcuuggaag | 20 | 97 | 0.0 | 2 |
| 75 | Group2_Unigene_BMK.74236_1360820 | aly-miR4231 | MIR477 | aagaccuuuugugacggcucaugc | 24 | 102 | 4.5 | 17 |
| 76 | CL22956Contig1_322565 | osa-miR812o-3p | MIR1883 | caacgguccgguccgaaaauggaa | 24 | 115 | 0.3 | 2 |
| 77 | CL9633Contig1_292728 | osa-miR2876-5p |  | gauugcuguggauuuggagaaga | 23 | 114 | 0.1 | 2 |
| 78 | Group1_Unigene_BMK.7537_936473 | sbi-miR5568d-3p | MIR5157 | caaguugugcugucucaaaaaguc | 24 | 114 | 5.3 | 30 |
| 79 | Group2_Unigene_BMK.15411_971925 | bdi-miR7750-3p |  | gauggucgcucgagcgug | 18 | 109 | 1.1 | 5 |
| 80 | CL22738Contig1_61999 | bdi-miR7726a-5p |  | agaugaugagagcuucgagccuga | 24 | 114 | 0.0 | 3 |
| 81 | Group2_Unigene_BMK.35892_1108432 | bra-miR9563b-3p |  | aaauuaagcuguaccaaacagggc | 24 | 114 | 4.8 | 38 |
| 82 | CL22479Contig1_234494 | ath-miR841a-3p |  | uuuucuagauauggugggucc | 21 | 109 | 3.2 | 8 |
| 83 | CL4803Contig1_366120 | pvu-miR2118 |  | augccgauccggaucguaggaucu | 24 | 114 | 0.1 | 3 |
| 84 | Group1_Unigene_BMK.41654_746253 | gma-miR482a-5p |  | ugaauuuggauccucuccuugagc | 24 | 114 | 0.2 | 23 |
| 85 | Group2_Unigene_BMK.71441_1348388 | gma-miR319p |  | auuuggaccaagccugacaguaag | 24 | 114 | 4.1 | 10 |
| 86 | CL25590Contig1_70619 | mtr-miR2590a | MIR393 | cucuaaagaauucucaucacucc | 23 | 112 | 0.3 | 2 |
| 87 | Group2_Unigene_BMK.11710_955285 | ptc-miR7840 |  | gaaggaguuguugcuugagcgacc | 24 | 114 | 0.4 | 3 |
| 88 | Group2_Unigene_BMK.5977_1300964 | ptc-miR319i |  | uugggcuggauacugaguuauc | 22 | 111 | 0.7 | 4 |
| 89 | Group1_Unigene_BMK.71687_926459 | zma-miR166h-5p |  | ugaaugaccaaaccucucaca | 21 | 92 | 29.8 | 50 |
| 90 | Group1_Unigene_BMK.53795_860370 | ath-miR8183 |  | uuuaguugugucaauuugaucucc | 24 | 114 | 1.4 | 5 |
| 91 | Group2_Unigene_BMK.10496_950042 | zma-miR171g-5p | MIR5272 | aauugacuaucucuggcacacagu | 24 | 114 | 1.2 | 5 |
| 92 | CL19035Contig1_399746 | sbi-miR5381 |  | cagaucugacuccggucuucggau | 24 | 114 | 1.6 | 7 |
| 93 | Group2_Unigene_BMK.11969_956441 | zma-miR395e-5p |  | uuucccuuucccuuuccuuuucc | 23 | 108 | 0.3 | 2 |
| 94 | CL10456Contig1_470022 | bdi-miR7714-5p | MIR395 | aauuuucuaccaaacacucacaug | 24 | 114 | 0.8 | 3 |
| 95 | CL6830Contig1_463164 | ath-miR168a-5p |  | gcgcuuggagcuuuuuaaaacacu | 24 | 113 | 3.5 | 10 |
| 96 | CL21889Contig1_319550 | ptc-miR396f | MIR7717 | guccacggagaaguaaacggcggc | 24 | 114 | 1.1 | 3 |
| 97 | CL1911Contig1_184453 | hvu-miR6207 |  | uggacgacgugucucucgug | 20 | 112 | 0.0 | 11 |
| 98 | Group1_Unigene_BMK.62619_891469 | osa-miR5821 |  | cggacggaugcggauugauuu | 21 | 95 | 0.2 | 20 |
| 99 | Group1_Unigene_BMK.39624_721083 | ath-miR869.1 |  | guugguucuuugguugauuuu | 21 | 111 | 0.3 | 2 |
| 100 | Group2_Unigene_BMK.32903_1080416 | sbi-miR6235-5p |  | augugagagguuugauaaaaaauu | 24 | 114 | 1.4 | 5 |
| 101 | CL23613Contig1_324766 | mtr-miR2630a | MIR2630 | cgguuuuggucacucauugaggc | 23 | 114 | 0.5 | 3 |
| 102 | Group2_Unigene_BMK.71973_1350885 | bdi-miR5174c-3p | MIR6457 | caauuuugaagccuccgcucgagc | 24 | 114 | 0.2 | 2 |
| 103 | Group2_Unigene_BMK.44348_1214144 | ath-miR319a |  | uuggacuggugagcucaucauc | 22 | 112 | 2.9 | 10 |
| 104 | Group2_Unigene_BMK.72865_1354993 | lja-miR7531 |  | cguguuuuagugacuccgcucgag | 24 | 114 | 3.7 | 9 |
| 105 | Group2_Unigene_BMK.44717_1219504 | osa-miR2100-5p | MIR9408 | cucucucaaagaacuguuguuccu | 24 | 114 | 0.9 | 4 |
| 106 | CL5786Contig1_196525 | gma-miR4996 |  | gagaagcugaucuauguaugaca | 23 | 109 | 0.7 | 2 |
| 107 | CL14260Contig1_478773 | osa-miR5515 | MIR7726 | acgaugguccaggcacugcacgcg | 24 | 114 | 0.3 | 2 |
| 108 | Group2_Unigene_BMK.71224_1347474 | ath-miR319a | MIR159 | uuggacugaagggggcuccc | 20 | 110 | 1.4 | 7 |
| 109 | Group2_Unigene_BMK.39451_1148377 | ath-miR782 | MIR5224 | ucaaacacaucgggaacauug | 21 | 113 | 0.0 | 2 |
| 110 | Group2_Unigene_BMK.60353_1303142 | ath-miR169a-5p | MIR169_2 | gagccaaggauacugaucaucacu | 24 | 113 | 1.3 | 4 |
| 111 | Group1_Unigene_BMK.30453_635412 | gra-miR7486i |  | ucugacgucgccucagcucggc | 22 | 112 | 0.0 | 11 |
| 112 | Group1_Unigene_BMK.15584_548367 | ath-miR472-5p |  | guggucgacuguggaugucacggu | 24 | 114 | 0.8 | 12 |
| 113 | Group1_Unigene_BMK.68667_914583 | gma-miR9767 |  | auggaaugguuggaggccggcaug | 24 | 101 | 3.3 | 6 |
| 114 | CL5691Contig1_23074 | ath-miR166a-5p |  | cgacuguuuggacccggaggguug | 24 | 114 | 0.0 | 2 |
| 115 | CL21958Contig1_407004 | mtr-miR5257 | MIR5998 | ucaaguagaguuugugcucuggugc | 25 | 115 | 6.9 | 5 |
| 116 | Group2_Unigene_BMK.60663_1304387 | gma-miR5761a |  | cuuugugugcucgaacagugaagu | 24 | 114 | 3.9 | 10 |
| 117 | Group2_Unigene_BMK.5048_1267308 | mdm-miR482a-5p |  | aggaauggaaaaugcucaagaaug | 24 | 114 | 0.5 | 3 |
| 118 | Group2_Unigene_BMK.39605_1150110 | cre-miR912 |  | uggauugagggauuuggag | 20 | 109 | 0.4 | 3 |
| 119 | CL19882Contig1_401776 | vvi-miR3631a-5p |  | cauguugaugggacuugggcagug | 24 | 111 | 0.1 | 2 |
| 120 | Group1_Unigene_BMK.16438_552036 | ath-miR420 |  | aaaacuaauuacaaaguagagggug | 25 | 114 | 2.5 | 6 |
| 121 | Group1_Unigene_BMK.37364_696840 | osa-miR2101-5p |  | ucauguuugggccuaauacuaa | 22 | 113 | 0.0 | 4 |
| 122 | CL31317Contig1_256905 | bdi-miR5175a | MIR3630 | cagaauuucucuacuacucugcgu | 24 | 114 | 7.5 | 7 |
| 123 | Group1_Unigene_BMK.38037_703962 | ppt-miR1029 |  | ucucucucucggcuuggag | 20 | 109 | 1.3 | 3 |
| 124 | Group2_Unigene_BMK.50808_1268477 | bdi-miR7729a-5p |  | aguuuucaucgccggagacagugc | 24 | 114 | 0.2 | 3 |
| 125 | Group2_Unigene_BMK.64130_1317662 | mtr-miR5753 | MIR5780 | guuuugaucuccuguugaagaugg | 24 | 114 | 1.2 | 6 |
| 126 | CL28847Contig1_77575 | stu-miR8016 | MIR482 | uuuuuugaaauuagacugaccucc | 24 | 114 | 3.4 | 9 |
| 127 | Group1_Unigene_BMK.30485_635795 | ath-miR157a-3p |  | ucucucuaucucucuaca | 18 | 108 | 0.0 | 2 |
| 128 | Group2_Unigene_BMK.68987_1337794 | ptc-miR396f |  | auccacggaggaccuuacucgccg | 24 | 114 | 0.3 | 3 |
| 129 | Group2_Unigene_BMK.42016_1180980 | gra-miR7504c |  | gagagaaauguaccguuggguggg | 24 | 114 | 0.8 | 4 |
| 130 | Group1_Unigene_BMK.36331_686670 | gra-miR7504c |  | gagagaaauguaccguuggguggg | 24 | 114 | 0.8 | 4 |
| 131 | Group1_Unigene_BMK.28128_617362 | osa-miR5073 |  | uuuugguguuacgcucgagcgggu | 24 | 114 | 1.3 | 6 |
| 132 | Group2_Unigene_BMK.46880_1254292 | gma-miR1512a-3p | MIR474 | gcuuuaagugacucggucuggccg | 24 | 114 | 2.0 | 6 |
| 133 | Group2_Unigene_BMK.18440_986990 | osa-miR2929 |  | gucaagggucacugguucaacugu | 24 | 114 | 0.6 | 5 |
| 134 | Group1_Unigene_BMK.70411_921527 | sbi-miR5568e-5p | MIR5272 | cauguuuuagaucuggacacugcc | 24 | 114 | 0.1 | 2 |
| 135 | Group2_Unigene_BMK.49791_1264903 | sbi-miR5568e-5p | MIR5272 | cauguuuuagaucuggacacugcc | 24 | 114 | 0.0 | 2 |
| 136 | Group2_Unigene_BMK.74496_1361897 | ath-miR2938 | MIR2655 | aaucuuuuuaagccucuuaacaug | 24 | 114 | 0.3 | 2 |
| 137 | Group1_Unigene_BMK.61847_888828 | mtr-miR5256 | MIR8674 | aaauggaucacagacacucacgu | 23 | 113 | 0.3 | 3 |
| 138 | Group2_Unigene_BMK.56446_1288539 | gma-miR9767 | MIR8710 | uuggaaugaugaaccuuaguc | 21 | 114 | 1.8 | 2 |
| 139 | Group2_Unigene_BMK.56446_1288542 | gma-miR9767 | MIR8710 | uuggaaugaugaaccuuaguc | 21 | 114 | 1.8 | 2 |
| 140 | Group2_Unigene_BMK.26971_1036600 | ptc-miR7825 | MIR5303 | cugaagaaucugaaccuaauucuc | 24 | 112 | 1.5 | 4 |
| 141 | Group2_Unigene_BMK.71697_1349603 | mtr-miR2607 |  | augugauuugucacuacugcguug | 24 | 114 | 0.6 | 2 |
| 142 | Group1_Unigene_BMK.68908_915592 | cpa-miR8153 | MIR6143 | agcacuguauucaaccugguggag | 24 | 114 | 2.0 | 4 |
| 143 | Group2_Unigene_BMK.36958_1119923 | ghr-miR7494 | MIR7494 | ugcuuguggaucaauugau | 19 | 109 | 0.0 | 2 |
| 144 | CL25590Contig1_70607 | bdi-miR5176-3p |  | agugaugagaauucuuuagagugu | 24 | 114 | 2.7 | 8 |
| 145 | CL30269Contig1_166900 | stu-miR8041a-3p |  | cugauguauuuggauuugggaucu | 24 | 114 | 0.0 | 2 |
| 146 | Group2_Unigene_BMK.42987_1194606 | osa-miR5519 | MIR2629 | cggcagaaguuccucaaca | 19 | 109 | 0.0 | 2 |
| 147 | Group2_Unigene_BMK.8370_1373185 | bdi-miR9480a |  | caugugagugguugguaaaauagu | 24 | 111 | 0.6 | 2 |
| 148 | CL20032Contig1_314672 | ath-miR780.2 |  | cucuucgucgcgagaucugggaag | 24 | 114 | 0.4 | 2 |
| 149 | Group1_Unigene_BMK.35816_681660 | zma-miR171b-5p | MIR5565 | cauauuggccgagaugauuaucau | 24 | 114 | 1.2 | 4 |
| 150 | Group1_Unigene_BMK.39687_721930 | zma-miR827-5p | MIR6161 | auuguuggaauuccgugcaagagu | 24 | 114 | 0.0 | 3 |
| 151 | Group2_Unigene_BMK.35270_1102408 | zma-miR827-5p | MIR6161 | auuguuggaauuccgugcaagagu | 24 | 114 | 0.0 | 3 |

| Table S6. All novel miRNA discovered in the two camellia species during seed natural drying | | | | | | | |
| --- | --- | --- | --- | --- | --- | --- | --- |
| **Number** | Pre-**miRNA** | **miRNA family** | **Mature sequence** | **Length (nt)** | **LP(nt)** | **score total** | **total read count** |
| 1 | Group1_Unigene_BMK.31136_640883 | MIR482 | uuuccaaguccacccauuccua | 22 | 108 | 19652.3 | 38545 |
| 2 | Group2_Unigene_BMK.31794_1071090 | MIR482 | uuuccaaguccacccauuccua | 22 | 112 | 19652.3 | 38545 |
| 3 | Group2_Unigene_BMK.60122_1302355 | MIR1520 | guuauuggauggugauaccacgug | 24 | 113 | 1.2 | 20652 |
| 4 | CL1888Contig1_271219 | MIR482 | uuuccuaaaccacccauuccuc | 22 | 112 | 1.8 | 12196 |
| 5 | Group1_Unigene_BMK.37987_703482 | MIR482 | uuuccaagaccacccaugccga | 22 | 112 | 2.1 | 8405 |
| 6 | Group2_Unigene_BMK.25259_1025467 | MIR482 | uuuccaagaccacccaugccga | 22 | 109 | 2.1 | 8409 |
| 7 | Group2_Unigene_BMK.25259_1025496 | MIR482 | uuuccaagaccacccaugccga | 22 | 112 | 2.2 | 8409 |
| 8 | Group2_Unigene_BMK.983_1379933 |  | gugcugucuaucgucgucaug | 21 | 110 | 6401.9 | 12555 |
| 9 | Group1_Unigene_BMK.24633_595825 |  | gugcugucuaucgucgucaug | 21 | 110 | 6401.9 | 12555 |
| 10 | Group2_Unigene_BMK.58728_1297086 | MIR5565 | uucccuaauugaugucggacaauu | 24 | 114 | 3033.1 | 5947 |
| 11 | Group1_Unigene_BMK.35465_678535 | MIR167_1 | uuccggcugugacaguguaggcac | 24 | 114 | 2.1 | 12014 |
| 12 | Group2_Unigene_BMK.29302_1052092 | MIR773 | auugccgacucaaaaacauuugcg | 24 | 114 | 1591.4 | 3121 |
| 13 | Group2_Unigene_BMK.30664_1062471 |  | auggcucaaaacuguuaaaacgac | 24 | 114 | 2.3 | 2957 |
| 14 | Group2_Unigene_BMK.24449_1019737 |  | auggagccucgagugaagagc | 21 | 111 | 830.9 | 1631 |
| 15 | CL14060Contig1_389564 |  | gauacaucgugcgauugggagugu | 24 | 114 | 2.1 | 2288 |
| 16 | CL16983Contig1_484318 | MIR393 | uaaaugcgaucccuugggaau | 21 | 111 | 348.2 | 681 |
| 17 | Group1_Unigene_BMK.35266_676587 | MIR535 | gugcucucuaucgucgucaugc | 22 | 111 | 691.9 | 1355 |
| 18 | Group1_Unigene_BMK.744_936047 |  | aaguuaguuuguuuggcauauucu | 24 | 113 | 0.6 | 698 |
| 19 | Group2_Unigene_BMK.18125_985418 |  | uuuggucauuuauuugguaga | 21 | 94 | 0.1 | 103 |
| 20 | CL21052Contig1_317401 |  | auugugucuaauugguauguuugu | 24 | 113 | 1.6 | 600 |
| 21 | CL26875Contig1_159660 |  | cuuuccccuuucccuuuccaaauc | 24 | 108 | 68.1 | 134 |
| 22 | CL296Contig1_437746 | MIR159 | cuccgaucgaaugagacccuuc | 22 | 112 | 1.3 | 588 |
| 23 | Group2_Unigene_BMK.63506_1315063 |  | ggaaagggaaagggaaaaggu | 21 | 95 | 0.2 | 300 |
| 24 | CL18311Contig1_311011 |  | auggccugacagcaaaugcucacg | 24 | 114 | 2.2 | 211 |
| 25 | Group1_Unigene_BMK.42407_756074 |  | acuacaaauuuggucgcggaaggu | 24 | 114 | 176.5 | 345 |
| 26 | Group2_Unigene_BMK.52890_1275908 | MIR477 | ggaaaucuuuggggagaaugaacg | 24 | 114 | 1.9 | 126 |
| 27 | Group2_Unigene_BMK.17316_981512 |  | ugguagaauauuugguaauucauu | 24 | 114 | 2.0 | 69 |
| 28 | Group2_Unigene_BMK.7016_1342668 |  | cuuuuaagcugcaccaaacagggc | 24 | 114 | 0.5 | 56 |
| 29 | Group2_Unigene_BMK.44346_1214103 | MIR8032 | aauaucagucgcuauaaguagguu | 24 | 104 | 1.4 | 214 |
| 30 | CL2440Contig1_359627 | MIR1861 | cauacuugcagcagaaucugagaa | 24 | 114 | 0.6 | 39 |
| 31 | CL23878Contig1_325691 |  | aucuuuggcaaguagaauagcagg | 24 | 114 | 0.8 | 174 |
| 32 | CL2938Contig1_449792 |  | aaagugugugaacaaaauuguucu | 24 | 114 | 141.0 | 275 |
| 33 | CL26801Contig1_333530 |  | aaauggugggcugugguccuugug | 24 | 111 | 1.5 | 66 |
| 34 | Group2_Unigene_BMK.33056_1081836 | MIR160 | ucggugagugacucauacagg | 21 | 111 | 347.2 | 689 |
| 35 | Group2_Unigene_BMK.53901_1279310 | MIR2592 | uagacggcuagccugaauaagggu | 24 | 106 | 23.5 | 45 |
| 36 | Group1_Unigene_BMK.21704_578939 |  | uccaaacuguacccauuguagaug | 24 | 114 | 0.5 | 22 |
| 37 | Group1_Unigene_BMK.2067_573693 | MIR3630 | guguccgacacucucucuaacgug | 24 | 113 | 1.2 | 23 |
| 38 | Group2_Unigene_BMK.23778_1015561 |  | uuaauggucugaguuguaucuu | 22 | 112 | 0.6 | 223 |
| 39 | Group1_Unigene_BMK.29062_624230 | MIR533 | cuaggaugucacggcugugaggag | 24 | 114 | 44.5 | 95 |
| 40 | Group2_Unigene_BMK.23997_1016824 |  | ccgauccccgauugugaaaaucuc | 24 | 114 | 1.1 | 21 |
| 41 | CL9644Contig1_380257 |  | cuguguuuggaucauggauu | 20 | 95 | 1.4 | 45 |
| 42 | Group1_Unigene_BMK.28669_621396 |  | uaugaauaaagauugggacucccu | 24 | 108 | 1.8 | 26 |
| 43 | Group2_Unigene_BMK.38504_1137258 |  | uuguguuuggaucauggauuu | 21 | 112 | 32.9 | 63 |
| 44 | Group1_Unigene_BMK.23831_591394 | MIR7696 | aaguucuguuuugauaauucaaac | 24 | 114 | 0.8 | 20 |
| 45 | Group2_Unigene_BMK.34396_1093900 | MIR7696 | aaguucuguuuugauaauucaaac | 24 | 114 | 0.8 | 20 |
| 46 | Group1_Unigene_BMK.62191_890098 |  | uguaacgguugcgguaacugg | 21 | 91 | 1.2 | 16 |
| 47 | CL15915Contig1_306240 | MIR7993 | ucauuccauggacuaacucaug | 22 | 112 | 1.8 | 139 |
| 48 | Group1_Unigene_BMK.25646_601906 |  | cauugccucgguuugcgugccc | 22 | 112 | 77.8 | 153 |
| 49 | CL2942Contig1_14758 | MIR5067 | gugugcauucggacucagaaggug | 24 | 114 | 1.6 | 32 |
| 50 | Group1_Unigene_BMK.16376_551753 | MIR398 | aguuccagcuguaaacacuacu | 22 | 112 | 1.0 | 97 |
| 51 | Group1_Unigene_BMK.69742_918987 |  | auagcucgguuguugaagacuagg | 24 | 100 | 1.7 | 25 |
| 52 | CL25536Contig1_330427 |  | caaucuccucgacgucguaguagg | 24 | 114 | 1.5 | 25 |
| 53 | Group1_Unigene_BMK.21471_577819 |  | uggaaaggaguacaaugugau | 21 | 109 | 0.7 | 90 |
| 54 | Group2_Unigene_BMK.5286_1275805 |  | gaaaaagauagaugaaaugugaau | 24 | 114 | 1.6 | 25 |
| 55 | Group1_Unigene_BMK.46148_809785 | MIR159 | aggagugauggaggcucucagagu | 24 | 114 | 0.3 | 11 |
| 56 | CL22840Contig1_235405 | MIR159 | aggagugauggaggcucucagagu | 24 | 114 | 0.3 | 11 |
| 57 | Group1_Unigene_BMK.12513_533812 |  | cguuacguaacgguuguggugguu | 24 | 114 | 1.3 | 59 |
| 58 | CL21760Contig1_495493 |  | ucuugaccugugauuucuuuggga | 24 | 114 | 60.8 | 117 |
| 59 | Group1_Unigene_BMK.49067_844300 | MIR5387 | aagcuccggccugggcucaagugg | 24 | 114 | 11.9 | 21 |
| 60 | Group1_Unigene_BMK.60465_884171 | MIR7997 | cuauguuacauggacucuuc | 20 | 114 | 1.2 | 12 |
| 61 | Group1_Unigene_BMK.36099_684338 |  | ugugugcuacuuucggacucc | 21 | 111 | 94.9 | 195 |
| 62 | Group2_Unigene_BMK.12810_960476 |  | ucuuagcucgguccugaagcgugu | 24 | 114 | 0.9 | 15 |
| 63 | CL23127Contig1_498764 |  | cugucucuggauuucaauacggcc | 24 | 114 | 3.5 | 5 |
| 64 | Group2_Unigene_BMK.41943_1179945 |  | cugucucuggauuucaauacggcc | 24 | 114 | 3.5 | 5 |
| 65 | CL19777Contig1_314088 |  | agacuguguuugguucauggauuu | 24 | 111 | 1.5 | 18 |
| 66 | CL28290Contig1_424336 | MIR839 | ucacucaugggaggcuugugcucu | 24 | 92 | 3.5 | 7 |
| 67 | CL948Contig1_6755 |  | auuaccaacgguaugaugucucuu | 24 | 114 | 0.5 | 23 |
| 68 | Group2_Unigene_BMK.38504_1137263 |  | agauuguguuuggaucaugga | 21 | 113 | 1.4 | 29 |
| 69 | Group2_Unigene_BMK.45260_1228779 |  | uauauaggacugagacauagcugu | 24 | 114 | 0.4 | 17 |
| 70 | CL16456Contig1_483269 |  | ugugaauauucacauugacgugug | 24 | 113 | 0.4 | 14 |
| 71 | Group2_Unigene_BMK.42599_1188665 | MIR1023 | aucacacuccccguccccgauccc | 24 | 114 | 2.0 | 15 |
| 72 | Group1_Unigene_BMK.37183_695405 |  | uuuugccgacuaaaacugacuuu | 23 | 113 | 1.1 | 47 |
| 73 | CL30170Contig1_254198 |  | uggaagugcaagugagccugugu | 23 | 112 | 1.6 | 10 |
| 74 | CL19506Contig1_489507 |  | ucgagcaggaaaauuguagcu | 21 | 111 | 0.0 | 10 |
| 75 | Group2_Unigene_BMK.30181_1058640 | MIR7484 | cgugaaaaaccaguccgcucgagc | 24 | 113 | 1.4 | 17 |
| 76 | Group2_Unigene_BMK.24252_1018543 |  | guauauauauauagauauuugagc | 24 | 114 | 1.2 | 12 |
| 77 | Group2_Unigene_BMK.48938_1261992 | MIR6462 | uuuaauguucuuaugagucuuugc | 24 | 114 | 3.4 | 4 |
| 78 | CL4378Contig1_191450 |  | uguuuggaucauggauuuggg | 21 | 114 | 0.1 | 24 |
| 79 | CL12087Contig1_125050 | MIR1888 | cuuaccaguuagaguuuaugaaga | 24 | 114 | 1.5 | 87 |
| 80 | Group1_Unigene_BMK.39098_715925 |  | gugaccuccauugcacuu | 18 | 91 | 0.6 | 9 |
| 81 | Group2_Unigene_BMK.5248_1274425 |  | uggcugugaugauguuuuc | 19 | 108 | 0.1 | 14 |
| 82 | Group2_Unigene_BMK.66230_1325887 |  | aaaaaucacucuuaucggggacgg | 24 | 97 | 1.6 | 18 |
| 83 | CL960Contig1_353479 | MIR6297 | aauaugccgucgcaaaaaguuccg | 24 | 114 | 3.1 | 5 |
| 84 | CL28135Contig1_76088 | MIR5284 | guuuagucgccaaaaguggagguu | 24 | 114 | 1.3 | 12 |
| 85 | Group2_Unigene_BMK.14145_966151 |  | uucgaacucaauaaaaauuuaacg | 24 | 113 | 0.5 | 22 |
| 86 | Group1_Unigene_BMK.4105_738225 |  | uggccgguugacuggaacagu | 21 | 111 | 0.2 | 10 |
| 87 | CL28094Contig1_336251 |  | agggcuagcagaagagggaaaggc | 24 | 114 | 1.5 | 14 |
| 88 | CL12431Contig1_298491 |  | uauccguguccgugcuucuuagau | 24 | 112 | 1.0 | 24 |
| 89 | CL19455Contig1_54014 |  | uguuuggaucauggauuuggg | 21 | 110 | 1.4 | 51 |
| 90 | Group2_Unigene_BMK.35875_1108373 |  | uuggaucuuuggccauuauguauu | 24 | 114 | 0.3 | 77 |
| 91 | Group2_Unigene_BMK.28329_1045828 | MIR6151 | aaucugugaagcguagaaucgaug | 24 | 114 | 3.0 | 6 |
| 92 | Group1_Unigene_BMK.14663_543730 |  | agacuuuugccgacuaaaaugagu | 24 | 114 | 1.0 | 12 |
| 93 | Group2_Unigene_BMK.10601_950447 | MIR8771 | aaggcgaucaggaacucaacggcc | 24 | 114 | 0.1 | 9 |
| 94 | Group1_Unigene_BMK.34588_670216 |  | uuugugucuaauguuaagaaaauu | 24 | 97 | 3.0 | 5 |
| 95 | CL17435Contig1_485238 |  | uuugugucuaauguuaagaaaauu | 24 | 114 | 3.0 | 5 |
| 96 | CL8605Contig1_290728 |  | auaaauaggacucauuucccaagg | 24 | 111 | 0.1 | 13 |
| 97 | Group2_Unigene_BMK.20139_995743 | MIR166 | uuucggaucagucuucacugc | 21 | 111 | 1.3 | 14 |
| 98 | Group1_Unigene_BMK.73698_934628 | MIR6140 | aaaugucaguacaauagauuuggu | 24 | 104 | 1.4 | 19 |
| 99 | CL5007Contig1_279728 |  | aaacuagugguguaaguagaauca | 24 | 114 | 0.1 | 20 |
| 100 | Group2_Unigene_BMK.58814_1297424 |  | auagagccgaacuuguugaauaug | 24 | 114 | 0.8 | 14 |
| 101 | Group1_Unigene_BMK.61925_889138 |  | uuucucugaagagaauuucucugg | 24 | 85 | 0.4 | 10 |
| 102 | Group1_Unigene_BMK.45675_802511 |  | agagagagagagagagagagagug | 24 | 81 | 1.5 | 21 |
| 103 | Group1_Unigene_BMK.36375_687138 |  | ggaucaguggcgaagaggagggug | 24 | 114 | 2.1 | 10 |
| 104 | Group2_Unigene_BMK.36747_1117795 | MIR1878 | auuuuuagcgacuaaauuuuguuu | 24 | 111 | 0.8 | 14 |
| 105 | Group2_Unigene_BMK.75_1363922 |  | uuaggaucauggaaaaaauaucaug | 25 | 115 | 0.4 | 35 |
| 106 | CL2738Contig1_14043 | MIR5272 | auuugauuuuuguucggaaguuca | 24 | 114 | 1.5 | 57 |
| 107 | Group1_Unigene_BMK.52032_854311 |  | uucaucuaacuugaucgucggagg | 24 | 110 | 0.3 | 17 |
| 108 | CL1154Contig1_268165 |  | uuucuugugguaguaauugcgaaca | 25 | 110 | 1.1 | 39 |
| 109 | CL24251Contig1_502434 |  | uuuacaaugauggaugaugauu | 22 | 112 | 1.6 | 18 |
| 110 | CL23559Contig1_411515 |  | aaaaaagacacagaagggagcaua | 24 | 114 | 1.3 | 11 |
| 111 | Group1_Unigene_BMK.44414_784344 |  | cgggcccgaguuuugcuggaggug | 24 | 109 | 1.2 | 14 |
| 112 | Group1_Unigene_BMK.34430_668680 |  | acugcccggagccggauaugacgua | 25 | 111 | 3.0 | 4 |
| 113 | Group2_Unigene_BMK.30670_1062502 |  | acugcccggagccggauaugacgua | 25 | 115 | 3.1 | 4 |
| 114 | CL8820Contig1_466849 |  | aauaaauuuagucgccaaaagugu | 24 | 114 | 4.2 | 9 |
| 115 | CL26040Contig1_71826 | bantam | gggaugcuuagcuuugacaaugau | 24 | 114 | 0.4 | 11 |
| 116 | Group1_Unigene_BMK.45124_794127 |  | auccauaucgucuggaagaucgg | 23 | 111 | 9.2 | 17 |
| 117 | Group2_Unigene_BMK.39199_1145135 |  | uuuccaucuguugauucugagucu | 24 | 114 | 0.9 | 13 |
| 118 | Group1_Unigene_BMK.68128_912297 | MIR159 | agcuccuuucgguccaaugac | 21 | 109 | 0.1 | 9 |
| 119 | Group1_Unigene_BMK.45108_793960 |  | aaggccuucugcucucugcugguu | 24 | 114 | 1.7 | 4 |
| 120 | Group2_Unigene_BMK.35753_1107175 |  | aguggacuguggaccugcgguaug | 24 | 114 | 0.0 | 6 |
| 121 | Group2_Unigene_BMK.74684_1362690 |  | uccgaugguggugcuguuguagc | 23 | 78 | 2.1 | 5 |
| 122 | CL30127Contig1_80060 |  | aguaagcgaaguacuuaugag | 20 | 111 | 2.3 | 3 |
| 123 | CL1468Contig1_9115 |  | gugggcuuggaccauuuggauu | 22 | 110 | 3.4 | 6 |

| Table S7. GO terms related with the lipid metabolism in the two camellia species during seed natural drying | | | |
| --- | --- | --- | --- |
| **Number** | **Go term** | **Target RNA** | **miRNA** |
|  | **Biological Process** |  |  |
| 1 | Acetyl-CoA biosynthetic process (GO:0006085) | CL351Contig1 | Group2_Unigene_BMK.18125_985418 |
| 2 | Acetyl-CoA metabolic process (GO:0006084) | Group2_Unigene_BMK.40874 | Group1_Unigene_BMK.30485_635795 |
|  |  | Group2_Unigene_BMK.25116 | Group1_Unigene_BMK.45675_802511 |
|  |  | Group1_Unigene_BMK.20889 | Group1_Unigene_BMK.30485_63579 |
|  |  | Group1_Unigene_BMK.39097 | Group1_Unigene_BMK.45675_802511 |
|  |  | CL7050Contig1 | Group1_Unigene_BMK.45675_802511 |
|  |  | Group1_Unigene_BMK.28170 | Group1_Unigene_BMK.30485_635795 |
|  |  | CL21673Contig1 | Group1_Unigene_BMK.45675_802511 |
|  |  | Group1_Unigene_BMK.15031 | Group1_Unigene_BMK.30485_635795 |
|  |  | CL1757Contig1 | Group1_Unigene_BMK.45675_802511 |
| 3 | Cellular lipid metabolic process (GO:0044255) | CL21165Contig1 | Group1_Unigene_BMK.30485_635795 |
| 4 | Diacylglycerol biosynthetic process (GO:0006651) | Group2_Unigene_BMK.40118 | Group1_Unigene_BMK.45675_802511 |
| 5 | Fatty acid beta-oxidation (GO:0006635) | Group1_Unigene_BMK.46735 | Group1_Unigene_BMK.45675_802511 |
|  |  | CL23075Contig1 | Group1_Unigene_BMK.30485_635795 |
|  |  | CL5846Contig1 | Group1_Unigene_BMK.30485_635795 |
|  |  | CL19463Contig1 | Group1_Unigene_BMK.45675_802511 |
|  |  | CL12771Contig1 | Group1_Unigene_BMK.30485_635795 |
|  |  | CL27395Contig1 | Group1_Unigene_BMK.45675_802511 |
|  |  | Group2_Unigene_BMK.30851 | Group2_Unigene_BMK.63506_1315063 |
|  |  | Group1_Unigene_BMK.26463 | Group1_Unigene_BMK.45675_802511 |
|  |  | CL5564Contig1 | Group2_Unigene_BMK.38504_1137258; CL9644Contig1_38025 |
|  |  | Group2_Unigene_BMK.44073 | Group1_Unigene_BMK.45675_802511 |
|  |  | CL25070Contig1 | Group1_Unigene_BMK.45675_802511 |
|  |  | CL19312Contig1 | Group1_Unigene_BMK.30485_635795 |
|  |  | CL23960Contig1 | Group1_Unigene_BMK.30485_635795; Group1_Unigene_BMK.45675_802511 |
|  |  | CL27582Contig1 | Group1_Unigene_BMK.45675_802511 |
|  |  | CL17211Contig1 | Group1_Unigene_BMK.45675_802511; Group1_Unigene_BMK.30485_63579 |
| 6 | Fatty acid biosynthetic process (GO:0006633) | CL31045Contig1 | Group1_Unigene_BMK.23434_588836; Group2_Unigene_BMK.34335_1093229 |
|  |  | CL29425Contig1 | Group1_Unigene_BMK.30485_635795; Group1_Unigene_BMK.45675_802511 |
|  |  | CL5821Contig1 | Group1_Unigene_BMK.45675_802511; Group1_Unigene_BMK.30485_635795 |
|  |  | CL11379Contig1 | Group1_Unigene_BMK.45675_802511; Group1_Unigene_BMK.30485_635795 |
|  |  | Group2_Unigene_BMK.32595 | Group1_Unigene_BMK.30485_63579 |
|  |  | Group1_Unigene_BMK.70820 | Group1_Unigene_BMK.30485_635795 |
|  |  | CL27791Contig1 | Group2_Unigene_BMK.39605_1150110 |
|  |  | CL21007Contig1 | Group1_Unigene_BMK.45675_802511 |
|  |  | CL24499Contig1 | Group1_Unigene_BMK.30485_63579 |
|  |  | CL20249Contig1 | Group1_Unigene_BMK.45675_802511 |
|  |  | CL8756Contig1 | Group2_Unigene_BMK.38504_1137258 |
|  |  | Group2_Unigene_BMK.35819 | Group2_Unigene_BMK.38504_1137258; CL19777Contig1_314088; Group2_Unigene_BMK.38504_113726 |
|  |  | Group2_Unigene_BMK.26367 | Group1_Unigene_BMK.30485_635795 |
|  |  | Group1_Unigene_BMK.25598 | Group1_Unigene_BMK.45675_802511 |
|  |  | CL32923Contig1 | Group1_Unigene_BMK.45675_802511 |
|  |  | Group1_Unigene_BMK.46529 | Group1_Unigene_BMK.45675_802511 |
|  |  | CL12653Contig1 | Group1_Unigene_BMK.45675_802511; Group1_Unigene_BMK.30485_635795 |
|  |  | Group2_Unigene_BMK.40874 | Group1_Unigene_BMK.30485_635795 |
|  |  | Group1_Unigene_BMK.23434 | Group2_Unigene_BMK.34335_1093229; Group1_Unigene_BMK.23434_588836 |
|  |  | Group2_Unigene_BMK.34335 | Group1_Unigene_BMK.23434_588836; Group2_Unigene_BMK.34335_1093229 |
|  |  | CL21434Contig1 | Group1_Unigene_BMK.45675_802511 |
|  |  | Group1_Unigene_BMK.35439 | Group1_Unigene_BMK.30485_635795 |
|  |  | CL2766Contig1 | Group1_Unigene_BMK.45675_802511 |
| 7 | Fatty acid catabolic process (GO:0009062) | Group1_Unigene_BMK.36844 | Group1_Unigene_BMK.45675_802511 |
|  |  | CL5944Contig1 | Group1_Unigene_BMK.45675_802511; Group1_Unigene_BMK.30485_63579 |
|  |  | Group2_Unigene_BMK.11169 | CL29078Contig1_514119 |
| 8 | Fatty acid metabolic process (GO:0006631) | Group2_Unigene_BMK.23869 | Group1_Unigene_BMK.30485_635795 |
| 9 | Fatty acid omega-oxidation (GO:0010430) | Group1_Unigene_BMK.14765 | Group1_Unigene_BMK.30485_635795 |
| 10 | Glycerol-3-phosphate metabolic process (GO:0006072) | CL6049Contig1 | Group2_Unigene_BMK.38504_1137258 |
|  |  | Group2_Unigene_BMK.38338 | Group1_Unigene_BMK.30485_635795 |
| 11 | Glycerophosphate shuttle (GO:0006127) | CL6049Contig1 | Group2_Unigene_BMK.38504_113725 |
| 12 | Biological Process: lipid biosynthetic process (GO:0008610);(1) | CL21165Contig1 | Group1_Unigene_BMK.30485_635795 |
| 13 | Biological Process: lipid catabolic process (GO:0016042);(4) | CL6167Contig1 | Group1_Unigene_BMK.30485_635795 |
|  |  | Group1_Unigene_BMK.19724 | Group1_Unigene_BMK.30485_63579 |
|  |  | Group2_Unigene_BMK.31430 | Group1_Unigene_BMK.45675_802511 |
|  |  | CL30312Contig1 | Group1_Unigene_BMK.45675_802511 |
| 14 | Lipid metabolic process (GO:0006629) | CL17263Contig1 | Group1_Unigene_BMK.45675_802511 |
|  |  | Group1_Unigene_BMK.32553 | Group1_Unigene_BMK.30485_635795 |
|  |  | CL19243Contig1 | Group1_Unigene_BMK.45675_802511 |
|  |  | Group2_Unigene_BMK.14673 | Group2_Unigene_BMK.38504_1137258; Group2_Unigene_BMK.38504_1137263 |
|  |  | Group1_Unigene_BMK.34487 | Group1_Unigene_BMK.30485_635795 |
|  |  | Group2_Unigene_BMK.42037 | Group1_Unigene_BMK.30485_63579 |
|  |  | CL20012Contig1 | Group1_Unigene_BMK.45675_802511 |
|  |  | CL2022Contig1 | Group1_Unigene_BMK.45675_80251 |
|  |  | Group1_Unigene_BMK.7430 | Group2_Unigene_BMK.32903_1080416 |
|  |  | Group1_Unigene_BMK.62256 | Group1_Unigene_BMK.30485_635795 |
|  |  | Group2_Unigene_BMK.41953 | Group1_Unigene_BMK.30485_635795 |
|  |  | Group1_Unigene_BMK.18205 | Group1_Unigene_BMK.30485_635795 |
|  |  | CL21543Contig1 | Group1_Unigene_BMK.45675_802511 |
|  |  | Group2_Unigene_BMK.31328 | Group1_Unigene_BMK.30485_635795 |
|  |  | Group1_Unigene_BMK.45898 | Group1_Unigene_BMK.38037_703962 |
|  |  | Group1_Unigene_BMK.44396 | Group1_Unigene_BMK.30485_635795 |
|  |  | Group1_Unigene_BMK.27637 | Group2_Unigene_BMK.38504_1137258; Group2_Unigene_BMK.38504_1137263 |
| 15 | Lipid storage (GO:0019915) | Group1_Unigene_BMK.44901 | Group1_Unigene_BMK.60465_884171 |
|  |  | Group1_Unigene_BMK.44888 | Group1_Unigene_BMK.37183_695405 |
|  |  | CL7109Contig1 | Group1_Unigene_BMK.45675_802511 |
| 16 | Lipid transport (GO:0006869) | Group2_Unigene_BMK.44073 | Group1_Unigene_BMK.45675_802511 |
|  |  | CL17211Contig1 | Group1_Unigene_BMK.45675_802511; Group1_Unigene_BMK.30485_635795 |
|  |  | CL4329Contig1 | Group1_Unigene_BMK.45675_802511 |
|  |  | Group2_Unigene_BMK.27382 | Group1_Unigene_BMK.45675_802511 |
|  |  | Group2_Unigene_BMK.29185 | Group1_Unigene_BMK.30485_635795 |
|  |  | CL32467Contig1 | Group1_Unigene_BMK.45675_802511 |
|  |  | CL23402Contig1 | Group1_Unigene_BMK.30485_635795 |
|  |  | Group2_Unigene_BMK.10939 | Group1_Unigene_BMK.30485_635795 |
| 17 | Very long-chain fatty acid metabolic process (GO:0000038) | Group1_Unigene_BMK.20909 | roup1_Unigene_BMK.45675_802511 |
| 18 | Wax biosynthetic process (GO:0010025) | CL15901Contig1 | Group1_Unigene_BMK.45675_802511 |
|  |  | CL32952Contig1 | Group1_Unigene_BMK.45675_802511 |
|  | **Molecular Function** |  |  |
| 1 | 1,2-diacylglycerol 3-beta-galactosyltransferase activity (GO:0046509) | CL7167Contig1 | Group1_Unigene_BMK.30485_635795 |
| 2 | 11-beta-hydroxysteroid dehydrogenase (NADP+) activity (GO:0070524) | Group1_Unigene_BMK.33995 | Group1_Unigene_BMK.30485_635795 |
| 3 | 2-alkenal reductase [NAD(P)] activity (GO:0032440) | CL26013Contig1 | Group1_Unigene_BMK.45675_802511 |
|  |  | CL930Contig1 | Group1_Unigene_BMK.30485_635795; Group2_Unigene_BMK.39605_1150110 |
|  |  | CL19595Contig1 | Group1_Unigene_BMK.21471_577819; Group1_Unigene_BMK.39098_715925 |
|  |  | Group1_Unigene_BMK.46796 | Group2_Unigene_BMK.58781_1297340 |
|  |  | CL1064Contig1 | CL19455Contig1_54014; unconservative_CL4378 |
|  |  | Contig1_191450 | CL9644Contig1_380257; Group2_Unigene_BMK.38504_1137258; Group2_Unigene_BMK.38504_1137263 |
|  |  | Group2_Unigene_BMK.23454 | Group1_Unigene_BMK.30485_635795 |
|  |  | Group1_Unigene_BMK.61619 | Group1_Unigene_BMK.45675_802511 |
|  |  | Group2_Unigene_BMK.36757 | Group1_Unigene_BMK.30485_635795 |
|  |  | CL17419Contig1 | Group1_Unigene_BMK.30485_635795 |
|  |  | Group2_Unigene_BMK.28684 | Group1_Unigene_BMK.43619_773032 |
|  |  | CL30915Contig1 | Group1_Unigene_BMK.30485_635795 |
|  |  | Group2_Unigene_BMK.40934 | Group1_Unigene_BMK.30485_635795 |
|  |  | CL3197Contig1 | Group1_Unigene_BMK.45675_802511 |
|  |  | Group1_Unigene_BMK.14545 | Group1_Unigene_BMK.30485_635795 |
|  |  | CL22738Contig1 | CL22738Contig1_61999 |
|  |  | CL18888Contig1 | Group1_Unigene_BMK.30485_635795; Group1_Unigene_BMK.45675_802511 |
|  |  | Group1_Unigene_BMK.37050 | Group1_Unigene_BMK.30485_635795 |
|  |  | Group1_Unigene_BMK.41784 | Group1_Unigene_BMK.45675_802511 |
|  |  | Group2_Unigene_BMK.71852 | Group1_Unigene_BMK.30485_635795 |
|  |  | Group1_Unigene_BMK.34072 | Group1_Unigene_BMK.45675_802511 |
|  |  | Group2_Unigene_BMK.45297 | Group2_Unigene_BMK.58781_1297340 |
|  |  | CL10089Contig1 | Group1_Unigene_BMK.45675_802511 |
|  |  | CL23606Contig1 | Group1_Unigene_BMK.30485_635795 |
|  |  | Group2_Unigene_BMK.19048 | Group1_Unigene_BMK.30485_635795 |
|  |  | CL30007Contig1 | Group1_Unigene_BMK.30485_635795 |
|  |  | CL31017Contig1 | Group1_Unigene_BMK.30485_635795 |
|  |  | CL17414Contig1 | Group1_Unigene_BMK.30485_635795 |
|  |  | CL32954Contig1 | Group1_Unigene_BMK.30485_635795 |
|  |  | Group1_Unigene_BMK.39684 | Group1_Unigene_BMK.39098_715925 |
|  |  | CL26688Contig1 | Group1_Unigene_BMK.30485_635795 |
|  |  | CL23587Contig1 | Group1_Unigene_BMK.45675_802511; Group1_Unigene_BMK.30485_635795 |
|  |  | CL10139Contig1 | Group1_Unigene_BMK.30485_635795 |
|  |  | CL865Contig1 | Group1_Unigene_BMK.30485_635795 |
| 4 | 3-hydroxyacyl-CoA dehydrogenase activity (GO:0003857) | Group1_Unigene_BMK.70039 | Group1_Unigene_BMK.30485_635795 |
|  |  | CL5944Contig1 | Group1_Unigene_BMK.45675_802511; Group1_Unigene_BMK.30485_635795 |
| 5 | 3-oxoacyl-[acyl-carrier-protein] reductase (NADPH) activity (GO:0004316) | Group2_Unigene_BMK.28614 | Group1_Unigene_BMK.45675_802511 |
|  |  | Group2_Unigene_BMK.22643 | Group1_Unigene_BMK.30485_635795 |
|  |  | Group1_Unigene_BMK.24337 | Group1_Unigene_BMK.30485_635795 |
|  |  | Group1_Unigene_BMK.68180 | Group1_Unigene_BMK.30485_635795 |
| 6 | 3-oxoacyl-[acyl-carrier-protein] synthase activity (GO:0004315) | Group1_Unigene_BMK.20909 | Group1_Unigene_BMK.45675_802511 |
|  |  | Group1_Unigene_BMK.70820 | Group1_Unigene_BMK.30485_635795 |
| 7 | 3R-hydroxyacyl-CoA dehydratase activity (GO:0080023) | CL22852Contig1 | Group1_Unigene_BMK.30485_635795 |
| 8 | N-acyltransferase activity (GO:0016410) | CL575Contig1 | Group1_Unigene_BMK.30485_635795 |
| 9 | S-acyltransferase activity (GO:0016417) | CL19312Contig1 | Group1_Unigene_BMK.30485_635795 |
| 10 | Acetyl-CoA C-acetyltransferase activity (GO:0003985) | CL3162Contig1 | Group1_Unigene_BMK.45675_802511 |
| 11 | Acetyl-CoA carboxylase activity (GO:0003989) | Group2_Unigene_BMK.32595 | Group1_Unigene_BMK.30485_635795 |
|  |  | Group2_Unigene_BMK.40874 | Group1_Unigene_BMK.30485_635795 |
|  |  | CL12653Contig1 | Group1_Unigene_BMK.45675_802511; Group1_Unigene_BMK.30485_635795 |
| 12 | Acyl-[acyl-carrier-protein] desaturase activity (GO:0045300) | Group2_Unigene_BMK.26367 | Group1_Unigene_BMK.30485_635795 |
|  |  | CL32923Contig1 | Group1_Unigene_BMK.45675_802511 |
|  |  | CL21434Contig1 | Group1_Unigene_BMK.45675_802511 |
| 13 | Delta12-fatty acid dehydrogenase activity (GO:0016720) | CL2766Contig1 | Group1_Unigene_BMK.45675_802511 |
| 14 | Diacylglycerol O-acyltransferase activity (GO:0004144) | Group1_Unigene_BMK.43406 | Group1_Unigene_BMK.45675_802511; Group1_Unigene_BMK.30485_635795 |
| 15 | Diacylglycerol kinase activity (GO:0004143) | CL22146Contig1 | Group2_Unigene_BMK.63506_1315063 |
|  |  | Group2_Unigene_BMK.42700 | Group2_Unigene_BMK.63506_1315063 |
|  |  | Group1_Unigene_BMK.41516 | Group1_Unigene_BMK.30485_635795 |
| 16 | Enoyl-CoA hydratase activity (GO:0004300) | CL5944Contig1 | Group1_Unigene_BMK.45675_802511; Group1_Unigene_BMK.30485_635795 |
|  |  | Group1_Unigene_BMK.41573 | Group1_Unigene_BMK.45675_802511; Group1_Unigene_BMK.30485_635795 |
| 17 | Fatty acid elongase activity (GO:0009922) | Group1_Unigene_BMK.35439 | Group1_Unigene_BMK.30485_635795 |
|  |  | CL32952Contig1 | Group1_Unigene_BMK.45675_802511 |
| 18 | Lipid binding (GO:0008289) | CL4329Contig1 | Group1_Unigene_BMK.45675_802511 |
|  |  | CL29298Contig1 | Group1_Unigene_BMK.2988_630525 |
|  |  | Group2_Unigene_BMK.43682 | Group1_Unigene_BMK.2988_630525 |
| 19 | Lipid transporter activity (GO:0005319) | CL32467Contig1 | Group1_Unigene_BMK.45675_802511 |
| 20 | Long-chain-(S)-2-hydroxy-long-chain-acid oxidase activity (GO:0052853) | Group2_Unigene_BMK.40297 | Group1_Unigene_BMK.45675_802511 |
| 21 | Long-chain-enoyl-CoA hydratase activity (GO:0016508) | CL5944Contig1 | Group1_Unigene_BMK.45675_802511; Group1_Unigene_BMK.30485_63579 |
| 22 | Medium-chain-(S)-2-hydroxy-acid oxidase activity (GO:0052854) | Group2_Unigene_BMK.40297 | Group1_Unigene_BMK.45675_802511 |
| 23 | Oleoyl-[acyl-carrier-protein] hydrolase activity (GO:0004320) | CL20249Contig1 | Group1_Unigene_BMK.45675_802511 |
| 24 | Omega peptidase activity (GO:0008242) | CL30218Contig1 | Group1_Unigene_BMK.30485_635795 |
| 25 | Omega-3 fatty acid desaturase activity (GO:0042389) | CL2766Contig1 | Group1_Unigene_BMK.45675_802511 |
| 26 | Palmitoyl-(protein) hydrolase activity (GO:0008474) | CL541Contig1 | Group1_Unigene_BMK.30485_635795 |
| 27 | Palmitoyl-[acyl-carrier-protein] hydrolase activity (GO:0016296) | CL20249Contig1 | Group1_Unigene_BMK.45675_802511 |
| 28 | Phosphatidylcholine phospholipase C activity (GO:0034480) | Group1_Unigene_BMK.23336 | Group1_Unigene_BMK.30485_635795 |
| 29 | Phosphatidylcholine-sterol O-acyltransferase activity (GO:0004607) | Group1_Unigene_BMK.32553 | Group1_Unigene_BMK.30485_635795 |
| 30 | Phosphatidyltransferase activity (GO:0030572) | CL32558Contig1 | Group1_Unigene_BMK.30485_635795 |
| 31 | Phospholipase A2 activity (GO:0004623) | Group2_Unigene_BMK.31430 | Group1_Unigene_BMK.45675_802511 |
|  |  | CL3301Contig1 | Group1_Unigene_BMK.30485_635795 |
| 32 | Phosphotransferase activity, for other substituted phosphate groups (GO:0016780) | Group1_Unigene_BMK.31579 | Group1_Unigene_BMK.30485_635795 |
| 33 | Phosphotransferase activity, phosphate group as acceptor (GO:0016776) | CL20147Contig1 | Group1_Unigene_BMK.45675_802511 |

| Table S8. KEGG enrichment related with the lipid metabolism in the two camellia species during seed natural drying | | | |
| --- | --- | --- | --- |
| **Number** | **Pathway** | **Target RNA** | **miRNA** |
| 1 | **Glycolysis / Gluconeogenesis(19)** | CL201Contig1 | Group1_Unigene_BMK.45675_802511 |
|  |  | CL28361Contig1 | Group1_Unigene_BMK.30485_635795 |
|  |  | CL31257Contig1 | Group1_Unigene_BMK.30485_635795 |
|  |  | CL7050Contig1 | Group1_Unigene_BMK.45675_802511 |
|  |  | CL9159Contig1 | Group1_Unigene_BMK.30485_635795 |
|  |  | CL9825Contig1 | Group1_Unigene_BMK.30485_635795 |
|  |  | Group1_Unigene_BMK.22348 | Group1_Unigene_BMK.30485_635795 |
|  |  | Group1_Unigene_BMK.23026 | Group1_Unigene_BMK.30485_635795 |
|  |  | Group1_Unigene_BMK.23788 | Group1_Unigene_BMK.45675_802511 |
|  |  | Group1_Unigene_BMK.23950 | Group1_Unigene_BMK.45675_802511 |
|  |  | Group1_Unigene_BMK.30900 | Group1_Unigene_BMK.45675_802511; Group1_Unigene_BMK.30485_635795; Group2_Unigene_BMK.23997_1016824; Group2_Unigene_BMK.42599_1188665; |
|  |  | Group1_Unigene_BMK.33835 | Group1_Unigene_BMK.45675_802511; Group1_Unigene_BMK.30485_635795 Group2_Unigene_BMK.38504_1137258; Group1_Unigene_BMK.43619_773032; CL19455Contig1_54014; CL4378Contig1_191450; |
|  |  | Group1_Unigene_BMK.42373 | CL9644Contig1_380257; Group2_Unigene_BMK.63506_1315063; CL4378Contig1_191450; CL19455Contig1_54014; CL19777Contig1_314088; |
|  |  | Group2_Unigene_BMK.23997 | Group2_Unigene_BMK.42599_1188665; Group2_Unigene_BMK.23997_1016824 |
|  |  | Group2_Unigene_BMK.26599 | Group1_Unigene_BMK.45675_802511 |
|  |  | Group2_Unigene_BMK.33010 | Group1_Unigene_BMK.45675_802511 |
|  |  | Group2_Unigene_BMK.35872 | Group1_Unigene_BMK.30485_635795 |
|  |  | Group2_Unigene_BMK.42133 | Group1_Unigene_BMK.30485_635795 |
|  |  | Group2_Unigene_BMK.44620 | CL19455Contig1_54014; Group2_Unigene_BMK.38504_1137258; CL4378Contig1_191450; CL9644Contig1_380257; |
| 2 | **Fatty acid biosynthesis(12)** | CL12653Contig1 | Group1_Unigene_BMK.45675_802511; Group1_Unigene_BMK.30485_635795 |
|  |  | CL20249Contig1 | Group1_Unigene_BMK.45675_802511 |
|  |  | CL21434Contig1 | Group1_Unigene_BMK.45675_802511 |
|  |  | CL24499Contig1 | Group1_Unigene_BMK.30485_635795 |
|  |  | CL32923Contig1 | Group1_Unigene_BMK.45675_802511 |
|  |  | CL4Contig7 | Group1_Unigene_BMK.45675_802511 |
|  |  | Group1_Unigene_BMK.20909 | Group1_Unigene_BMK.45675_802511 |
|  |  | Group1_Unigene_BMK.70820 | Group1_Unigene_BMK.30485_635795 |
|  |  | Group2_Unigene_BMK.26367 | Group1_Unigene_BMK.30485_635795 |
|  |  | Group2_Unigene_BMK.28614 | Group1_Unigene_BMK.45675_802511 |
|  |  | Group2_Unigene_BMK.32595 | Group1_Unigene_BMK.30485_635795 |
|  |  | Group2_Unigene_BMK.40874 | Group1_Unigene_BMK.30485_635795 |
| 3 | **Fatty acid elongation in mitochondria(1)** | CL541Contig1 | Group1_Unigene_BMK.30485_635795 |
| 4 | **Fatty acid metabolism(4)** | CL3162Contig1 | Group1_Unigene_BMK.45675_802511 |
|  |  | CL9825Contig1 | Group1_Unigene_BMK.30485_635795 |
|  |  | Group1_Unigene_BMK.21562 | Group1_Unigene_BMK.30485_635795 |
|  |  | Group2_Unigene_BMK.45431 | Group1_Unigene_BMK.30485_635795 |
| 5 | **Glycerolipid metabolism(9)** | CL21165Contig1 | Group1_Unigene_BMK.30485_635795 |
|  |  | CL22146Contig1 | Group2_Unigene_BMK.63506_1315063 |
|  |  | CL6765Contig1 | Group1_Unigene_BMK.30485_635795 |
|  |  | CL7167Contig1 | Group1_Unigene_BMK.30485_635795 |
|  |  | CL9825Contig1 | Group1_Unigene_BMK.30485_635795 |
|  |  | Group1_Unigene_BMK.43406 | Group1_Unigene_BMK.45675_802511; Group1_Unigene_BMK.30485_635795 |
|  |  | Group2_Unigene_BMK.38338 | Group1_Unigene_BMK.30485_635795 |
|  |  | Group2_Unigene_BMK.40118 | Group1_Unigene_BMK.45675_802511 |
|  |  | Group2_Unigene_BMK.42700 | Group2_Unigene_BMK.63506_1315063 |
| 6 | **Glycerophospholipid metabolism(10)** | CL18487Contig1 | Group1_Unigene_BMK.30485_635795; Group1_Unigene_BMK.45675_802511 |
|  |  | CL20012Contig1 | Group1_Unigene_BMK.45675_802511 |
|  |  | CL22146Contig1 | Group2_Unigene_BMK.63506_1315063 |
|  |  | CL32558Contig1 | Group1_Unigene_BMK.30485_635795 |
|  |  | CL6049Contig1 | Group2_Unigene_BMK.38504_1137258 |
|  |  | Group1_Unigene_BMK.23336 | Group1_Unigene_BMK.30485_635795 |
|  |  | Group1_Unigene_BMK.31579 | Group1_Unigene_BMK.30485_635795 |
|  |  | Group1_Unigene_BMK.32553 | Group1_Unigene_BMK.30485_635795 |
|  |  | Group2_Unigene_BMK.40118 | Group1_Unigene_BMK.45675_802511 |
|  |  | Group2_Unigene_BMK.42700 | Group2_Unigene_BMK.63506_1315063 |
| 7 | **Ether lipid metabolism(2)** | Group1_Unigene_BMK.23336 | Group1_Unigene_BMK.30485_635795 |
|  |  | Group1_Unigene_BMK.31579 | Group1_Unigene_BMK.30485_635795 |
| 8 | **Arachidonic acid metabolism(1)** | CL3Contig3 | Group2_Unigene_BMK.63506_1315063; Group1_Unigene_BMK.13350_537542 |
| 9 | **Sphingolipid metabolism(5)** | CL13176Contig1 | Group1_Unigene_BMK.45675_802511 |
|  |  | CL21007Contig1 | Group1_Unigene_BMK.45675_802511 |
|  |  | Group1_Unigene_BMK.29878 | Group1_Unigene_BMK.45675_802511; Group1_Unigene_BMK.30485_635795 |
|  |  | Group1_Unigene_BMK.45452 | Group1_Unigene_BMK.45675_802511 |
|  |  | Group2_Unigene_BMK.22527 | Group1_Unigene_BMK.30485_635795; Group1_Unigene_BMK.45675_802511 |
| 10 | **Pyruvate metabolism(20)** | CL12653Contig1 | unconservative_Group1_Unigene_BMK.45675_802511; Group1_Unigene_BMK.30485_635795 |
|  |  | CL23638Contig1 | Group1_Unigene_BMK.30485_635795 |
|  |  | CL28361Contig1 | Group1_Unigene_BMK.30485_635795 |
|  |  | CL29425Contig1 | Group1_Unigene_BMK.30485_635795; Group1_Unigene_BMK.45675_802511 |
|  |  | CL30465Contig1 | Group1_Unigene_BMK.45675_802511 |
|  |  | CL30563Contig1 | Group1_Unigene_BMK.30485_635795 |
|  |  | CL31257Contig1 | Group1_Unigene_BMK.30485_635795 |
|  |  | CL3162Contig1 | Group1_Unigene_BMK.45675_802511 |
|  |  | CL4952Contig1 | Group1_Unigene_BMK.45675_802511; Group1_Unigene_BMK.30485_635795 |
|  |  | CL4Contig7 | Group1_Unigene_BMK.45675_802511 |
|  |  | CL7050Contig1 | Group1_Unigene_BMK.45675_802511 |
|  |  | CL9825Contig1 | Group1_Unigene_BMK.30485_635795 |
|  |  | Group1_Unigene_BMK.14706 | Group2_Unigene_BMK.42987_1194606 |
|  |  | Group1_Unigene_BMK.23950 | Group1_Unigene_BMK.45675_802511 |
|  |  | Group1_Unigene_BMK.46529 | Group1_Unigene_BMK.45675_802511 |
|  |  | Group2_Unigene_BMK.32595 | Group1_Unigene_BMK.30485_635795 |
|  |  | Group2_Unigene_BMK.33010 | Group1_Unigene_BMK.45675_802511 |
|  |  | Group2_Unigene_BMK.35872 | Group1_Unigene_BMK.30485_635795 |
|  |  | Group2_Unigene_BMK.36413 | Group1_Unigene_BMK.45675_802511 |
|  |  | Group2_Unigene_BMK.40874 | Group1_Unigene_BMK.30485_635795 |
| 11 | **Biosynthesis of unsaturated fatty acids(7)** | CL21434Contig1 | Group1_Unigene_BMK.45675_802511 |
|  |  | CL22852Contig1 | Group1_Unigene_BMK.30485_635795 |
|  |  | CL2766Contig1 | Group1_Unigene_BMK.45675_802511 |
|  |  | CL32923Contig1 | Group1_Unigene_BMK.45675_802511 |
|  |  | CL32952Contig1 | Group1_Unigene_BMK.45675_802511 |
|  |  | Group2_Unigene_BMK.26367 | Group1_Unigene_BMK.30485_635795 |
|  |  | Group2_Unigene_BMK.28614 | Group1_Unigene_BMK.45675_802511 |
| 12 | **Starch and sucrose metabolism(28)** | CL10586Contig1 | Group1_Unigene_BMK.30485_635795 |
|  |  | CL10783Contig1 | Group1_Unigene_BMK.45675_802511 |
|  |  | CL11673Contig1 | Group1_Unigene_BMK.30485_635795 |
|  |  | CL13081Contig1 | Group1_Unigene_BMK.45675_802511 |
|  |  | CL13679Contig1 | Group1_Unigene_BMK.45675_802511 |
|  |  | CL13947Contig1 | Group1_Unigene_BMK.45675_802511 |
|  |  | CL1468Contig1 | CL1468Contig1_9115 |
|  |  | CL15838Contig1 | Group1_Unigene_BMK.45675_802511 |
|  |  | CL1973Contig1 | Group1_Unigene_BMK.45675_802511 |
|  |  | CL19905Contig1 | Group1_Unigene_BMK.30485_635795 |
|  |  | CL201Contig1 | Group1_Unigene_BMK.45675_802511 |
|  |  | CL21511Contig1 | Group1_Unigene_BMK.45675_802511 |
|  |  | CL24524Contig1 | Group1_Unigene_BMK.30485_635795 |
|  |  | CL25382Contig1 | Group1_Unigene_BMK.30485_635795 |
|  |  | CL28985Contig1 | Group1_Unigene_BMK.30485_635795 |
|  |  | CL3Contig12 | Group1_Unigene_BMK.43619_773032; Group2_Unigene_BMK.11969_956441; Group1_Unigene_BMK.13350_537542; CL26875Contig1_159660; Group2_Unigene_BMK.63506_1315063; |
|  |  | Group1_Unigene_BMK.29886 | Group1_Unigene_BMK.30485_635795; Group1_Unigene_BMK.45675_802511 |
|  |  | Group1_Unigene_BMK.30900 | Group1_Unigene_BMK.45675_802511 |
|  |  | Group1_Unigene_BMK.31207 | Group1_Unigene_BMK.30485_635795 |
|  |  | Group1_Unigene_BMK.35139 | Group1_Unigene_BMK.30485_635795 |
|  |  | Group1_Unigene_BMK.41328 | Group1_Unigene_BMK.45675_802511 |
|  |  | Group1_Unigene_BMK.44817 | Group1_Unigene_BMK.30485_635795 |
|  |  | Group1_Unigene_BMK.6380 | Group1_Unigene_BMK.30485_635795 |
|  |  | Group2_Unigene_BMK.23540 | Group1_Unigene_BMK.30485_635795 |
|  |  | Group2_Unigene_BMK.23997 | Group2_Unigene_BMK.42599_1188665; Group2_Unigene_BMK.23997_1016824 |
|  |  | Group2_Unigene_BMK.42133 | Group1_Unigene_BMK.30485_635795 |
|  |  | Group2_Unigene_BMK.42904 | Group1_Unigene_BMK.30485_635795 |
|  |  | Group2_Unigene_BMK.7152 | Group1_Unigene_BMK.30485_635795 |

| Table S9. miRNA of lipid metabolism targets and their putative functions | | | | |
| --- | --- | --- | --- | --- |
| **Pre-miRNA** | **miRNA** | **miRNA family** | **Target location** | **Target function** |
| **Fatty acid synthesis** | | | | |
| Group2_Unigene_BMK.9543_1378570 | bdi-miR5185m-3p | Unkown | Group2_Unigene_BMK.33511 | Biotin--[acetyl-CoA-carboxylase] ligase |
| Group2_Unigene_BMK.11969_956441 | zma-miR395e-5p | Unkown | CL27787Contig1 | Enoyl-(Acyl carrier protein) reductase |
| Group1_Unigene_BMK.23434_588836 | osa-miR1861a | MIR5067 | Group1_Unigene_BMK.23434 | 3-ketoacyl-CoA synthase III |
|  |  |  | Group2_Unigene_BMK.34335 | 3-ketoacyl-CoA synthase III |
|  |  |  | CL31045Contig1 | 3-ketoacyl-CoA synthase III |
| Group2_Unigene_BMK.39605_1150110 | cre-miR912 | Unkown | CL27791Contig1 | 3-ketoacyl-CoA synthase III |
| Group2_Unigene_BMK.34335_1093229 | osa-miR1861a | MIR5067 | Group2_Unigene_BMK.34335 | 3-ketoacyl-CoA synthase III |
|  |  |  | Group1_Unigene_BMK.23434 | 3-ketoacyl-CoA synthase III |
|  |  |  | CL31045Contig1 | 3-ketoacyl-CoA synthase III |
| **Fatty acid accumulation** | | | | |
| CL22738Contig1_61999 | bdi-miR7726a-5p | Unkown | CL22738Contig1 | S-acyltransferase |
| Group1_Unigene_BMK.37364_696840 | osa-miR2101-5p | Unkown | CL4217Contig1 | S-acyltransferase |
|  |  |  | Group2_Unigene_BMK.43738 | S-acyltransferase |
| CL19777Contig1_314088 | Unkown | Unkown | CL10827Contig1 | glycerol-3-phosphate transporter |
| CL4378Contig1_191450 | Unkown | Unkown | CL10827Contig1 | glycerol-3-phosphate transporter |
| CL19455Contig1_54014 | Unkown | Unkown | CL10827Contig1 | glycerol-3-phosphate transporter |
| **Fatty acid catabolism** |  |  |  |  |
| Group1_Unigene_BMK.37987_703484 | osa-miR2118e | MIR2118 | CL18627Contig1 | long-chain-alcohol oxidase FAO2 |
| Group2_Unigene_BMK.25259_1025465 | osa-miR2118e | MIR482 | CL18627Contig1 | long-chain-alcohol oxidase FAO2 |
| Group2_Unigene_BMK.25259_1025498 | osa-miR2118e | MIR482 | CL18627Contig1 | long-chain-alcohol oxidase FAO2 |
| Group1_Unigene_BMK.38037_703962 | ppt-miR1029 | Unkown | Group2_Unigene_BMK.73313 | triacylglycerol lipase |
|  |  |  | Group1_Unigene_BMK.33835 | glyceraldehyde-3-phosphate dehydrogenase |
| Group2_Unigene_BMK.50808_1268477 | bdi-miR7729a-5p | Unkown | Group2_Unigene_BMK.50808 | carboxylesterase |
| Group2_Unigene_BMK.24252_1018543 | Unkown | Unkown | CL18317Contig1 | Carboxylesterase |
| CL9644Contig1_380257 | Unkown | Unkown | Group2_Unigene_BMK.225 | Carboxylesterase |
| CL2440Contig1_359627 | Unkown | MIR1861 | CL2440Contig1 | lysophospholipase |
|  |  |  | Group1_Unigene_BMK.5361 | monoglyceride lipase |
| Group2_Unigene_BMK.38504_1137263 | Unkown | Unkown | Group2_Unigene_BMK.14673 | Lipases/Acylhydrolase |
|  |  |  | Group2_Unigene_BMK.35819 | Fatty acid hydroxylase |
|  |  |  | Group1_Unigene_BMK.27637 | Lipases/Acylhydrolase |
| Group2_Unigene_BMK.38504_1137258 | Unkown | Unkown | Group1_Unigene_BMK.27637 | Lipases/Acylhydrolase |
|  |  |  | Group2_Unigene_BMK.14673 | Lipases/Acylhydrolase |
|  |  |  | Group2_Unigene_BMK.35819 | Fatty acid hydroxylase |
|  |  |  | Group2_Unigene_BMK.225 | Carboxylesterase |
| Group2_Unigene_BMK.63506_1315063 | Unkown | Unkown | Group2_Unigene_BMK.42700 | Diacylglycerol kinase |
|  |  |  | CL22146Contig1 | Diacylglycerol kinase |
| **Fatty acid synthesis, accumulation, catabolism** | | | | |
| Group1_Unigene_BMK.30485_635795 | ath-miR157a-3p | Unkown | Group1_Unigene_BMK.69632 | Acetyl-CoA synthetase |
|  |  |  | CL9211Contig1 | Acetyl-CoA synthetase |
|  |  |  | Group2_Unigene_BMK.40874 | biotin carboxylase |
|  |  |  | CL24499Contig1 | hydroxyacyl-ACP dehydratase |
|  |  |  | Group1_Unigene_BMK.70820 | ketoacyl-ACP synthase III |
|  |  |  | Group1_Unigene_BMK.35439 | 3-ketoacyl-CoA synthase 11 |
|  |  |  | Group1_Unigene_BMK.24337 | Enoyl-(Acyl carrier protein) reductase |
|  |  |  | CL22925Contig1 | Enoyl-(Acyl carrier protein) reductase |
|  |  |  | Group1_Unigene_BMK.41573 | Enoyl-CoA hydratase |
|  |  |  | CL23751Contig1 | Enoyl-CoA hydratase |
|  |  |  | CL7225Contig1 | Enoyl-CoA hydratase |
|  |  |  | Group2_Unigene_BMK.22643 | Enoyl-(Acyl carrier protein) reductase |
|  |  |  | CL541Contig1 | Palmitoyl protein thioesterase |
|  |  |  | CL6349Contig1 | acyl-coenzyme A thioesteras |
|  |  |  | CL5821Contig1 | Delta(8)-fatty-acid desaturase |
|  |  |  | Group2_Unigene_BMK.26367 | stearoyl-acyl carrier protein desaturase |
|  |  |  | Group1_Unigene_BMK.21562 | Long chain acyl-CoA synthetase |
|  |  |  | Group2_Unigene_BMK.45431 | Long chain acyl-CoA synthetase |
|  |  |  | Group1_Unigene_BMK.29671 | acyltransferase |
|  |  |  | Group2_Unigene_BMK.62646 | acyltransferase |
|  |  |  | CL11379Contig1 | acyl carrier protein |
|  |  |  | CL32838Contig1 | lysophospholipase |
|  |  |  | Group1_Unigene_BMK.43406 | diacylglycerol O-acyltransferase |
|  |  |  | CL6765Contig1 | phospholipid:diacylglycerol acyltransferase |
|  |  |  | CL16839Contig1 | Phosphatidylinositol/phosphatidylcholine transfer protein |
|  |  |  | CL25768Contig1 | phosphatidylglycerol/phosphatidylinositol transfer protein |
|  |  |  | CL26688Contig1 | S-acyltransferase |
|  |  |  | CL19312Contig1 | S-acyltransferase |
|  |  |  | CL734Contig1 | Oleosin |
|  |  |  | CL6167Contig1 | Lipase |
|  |  |  | Group1_Unigene_BMK.62256 | lipase |
|  |  |  | Group1_Unigene_BMK.44396 | Lipase |
|  |  |  | Group2_Unigene_BMK.32595 | acetyl-coenzyme A carboxylase |
|  |  |  | CL12653Contig1 | acetyl-coenzyme A carboxylase |
|  |  |  | CL897Contig1 | acetate/butyrate--CoA ligase |
|  |  |  | Group2_Unigene_BMK.31328 | Acylhydrolase |
|  |  |  | Group1_Unigene_BMK.18205 | Acylhydrolase |
|  |  |  | Group1_Unigene_BMK.32553 | lecithin-cholesterol acyltransferase |
|  |  |  | CL32558Contig1 | CDP-alcohol phosphatidyltransferase |
|  |  |  | Group2_Unigene_BMK.7794 | Lipid phosphate phosphatase |
|  |  |  | Group1_Unigene_BMK.31579 | Choline/ethanolaminephosphotransferase |
|  |  |  | Group2_Unigene_BMK.10168 | Phosphatidylinositol/phosphatidylcholine transfer protein |
|  |  |  | CL7167Contig1 | Monogalactosyl diacylglycerol synthase |
|  |  |  | CL22852Contig1 | Very-long-chain (3R)-3-hydroxyacyl-CoA dehydratase |
|  |  |  | Group1_Unigene_BMK.41550 | Phospholipase A |
|  |  |  | CL2861Contig1 | Phospholipase A |
|  |  |  | Group1_Unigene_BMK.19724 | Phospholipase D |
|  |  |  | Group2_Unigene_BMK.42037 | Phosphoinositide phospholipase C |
|  |  |  | CL23511Contig1 | long-chain-alcohol O-fatty-acyltransferase |
|  |  |  | Group2_Unigene_BMK.225 | Carboxylesterase |
|  |  |  | Group1_Unigene_BMK.42775 | Carboxylesterase |
|  |  |  | Group2_Unigene_BMK.25623 | Carboxylesterase |
|  |  |  | CL18317Contig1 | Carboxylesterase |
|  |  |  | CL19766Contig1 | carboxylesterase |
|  |  |  | Group1_Unigene_BMK.41516 | Diacylglycerol kinase |
|  |  |  | CL18487Contig1 | CDP-diacylglycerol--serine O-phosphatidyltransferase |
| Group1_Unigene_BMK.45675_802511 | Unkown | Unkown | CL2018Contig1 | Acetyl-CoA synthetase |
|  |  |  | Group2_Unigene_BMK.45595 | Acetyl-CoA synthetase |
|  |  |  | Group1_Unigene_BMK.41573 | Enoyl-CoA hydratase |
|  |  |  | CL22013Contig1 | Enoyl-(Acyl carrier protein) reductase |
|  |  |  | CL32952Contig1 | very-long-chain enoyl-CoA reductase |
|  |  |  | Group1_Unigene_BMK.20909 | ketoacyl-ACP synthase II |
|  |  |  | CL21007Contig1 | delta(4)-desaturase |
|  |  |  | CL32923Contig1 | stearoy-l ACP desaturase |
|  |  |  | CL2766Contig1 | omega-3 fatty acid desaturase |
|  |  |  | CL20249Contig1 | Palmitoyl-acyl carrier protein thioesterase |
|  |  |  | CL21434Contig1 | acyl-[acyl-carrier-protein] desaturase |
|  |  |  | Group2_Unigene_BMK.43738 | S-acyltransferase |
|  |  |  | CL3162Contig1 | acetyl-CoA C-acetyltransferase |
|  |  |  | Group1_Unigene_BMK.24970 | acetyltransferase |
|  |  |  | Group2_Unigene_BMK.33271 | acyltransferase |
|  |  |  | CL11379Contig1 | acyl carrier protein |
|  |  |  | Group1_Unigene_BMK.25598 | Acyl carrier protein |
|  |  |  | Group1_Unigene_BMK.43406 | Diacylglycerol O-acyltransferase |
|  |  |  | Group2_Unigene_BMK.6287 | Diacylglycerol O-acyltransferase |
|  |  |  | Group2_Unigene_BMK.40118 | Lysophospholipid acyltransferase |
|  |  |  | CL25768Contig1 | phosphatidylglycerol/phosphatidylinositol transfer protein |
|  |  |  | CL26910Contig1 | Phosphatidylinositol/phosphatidylcholine transfer protein |
|  |  |  | CL22882Contig1 | Phosphatidylinositol/phosphatidylcholine transfer protein |
|  |  |  | CL422Contig1 | Oleosin |
|  |  |  | CL24190Contig1 | acetyl-CoA carboxylase |
|  |  |  | Group2_Unigene_BMK.43094 | acetyl-CoA carboxylase |
|  |  |  | CL12653Contig1 | acetyl-coenzyme A carboxylase |
|  |  |  | CL4Contig7 | acetyl-CoA carboxylase |
|  |  |  | CL19243Contig1 | Phosphoinositide phospholipase C |
|  |  |  | Group2_Unigene_BMK.31430 | Phospholipase A |
|  |  |  | CL2022Contig1 | Lipase |
|  |  |  | CL30312Contig1 | triacylglycerol lipase |
|  |  |  | CL18627Contig1 | Long-chain-alcohol oxidase FAO2 |
|  |  |  | Group1_Unigene_BMK.39524 | monooxygenase |
|  |  |  | Group1_Unigene_BMK.33241 | monooxygenase |
|  |  |  | CL12425Contig1 | diacylglycerol kinase |
|  |  |  | Group2_Unigene_BMK.25623 | carboxylesterase |
|  |  |  | CL18487Contig1 | Acyl-ACP thioesterase |
|  |  |  | Group1_Unigene_BMK.44917 | Acyl-CoA thioester hydrolase |
|  |  |  | CL24517Contig1 | Phospholipid methyltransferase |
|  |  |  | CL5944Contig1 | 3-hydroxyacyl-CoA dehydrogenase |

| Table S10. Differentially expressed miRNAs of lipid metabolism during *C.* *meiocarpa* seed natural drying | | | | | | |
| --- | --- | --- | --- | --- | --- | --- |
| **Pre**-**miRNA** | **S01** | **S02** | **FDR** | **log2FC** | **regulated** | **miRNA family** |
| Group1_Unigene_BMK.37987_703484 | 3263.101 | 4857.301 | 0.000 | 0.574 | up | MIR2118 |
| Group2_Unigene_BMK.25259_1025465 | 3263.101 | 4857.301 | 0.000 | 0.574 | up |  |
| Group2_Unigene_BMK.25259_1025498 | 3263.101 | 4857.301 | 0.000 | 0.574 | up |  |
|  | **S02** | **S03** |  |  |  |  |
| Group1_Unigene_BMK.37987_703484 | 4857.301 | 5408.928 | 0.008 | 0.155 | up | MIR2118 |
| Group2_Unigene_BMK.25259_1025465 | 4857.301 | 5408.928 | 0.008 | 0.155 | up | MIR482 |
| Group2_Unigene_BMK.25259_1025498 | 4857.301 | 5408.928 | 0.008 | 0.155 | up | MIR482 |
| Group2_Unigene_BMK.63506_1315063 | 405.494 | 644.437 | 0.006 | 0.668 | up |  |
|  | **S03** | **S04** |  |  |  |  |
| Group1_Unigene_BMK.45675_802511 | 57.927 | 3.843 | 0.002 | -3.914 | down |  |
| CL2440Contig1_359627 | 94.131 | 19.217 | 0.002 | -2.292 | down | MIR1861 |
| Group1_Unigene_BMK.23434_588836 | 238.949 | 96.083 | 0.000 | -1.314 | down | MIR5067 |
| Group2_Unigene_BMK.34335_1093229 | 238.949 | 96.083 | 0.000 | -1.314 | down | MIR5067 |
| CL19777Contig1_314088 | 217.226 | 111.456 | 0.005 | -0.963 | down |  |
| Group1_Unigene_BMK.37987_703484 | 5408.928 | 3101.544 | 0.000 | -0.802 | down | MIR2118 |
| Group2_Unigene_BMK.25259_1025465 | 5408.928 | 3101.544 | 0.000 | -0.802 | down | MIR482 |
| Group2_Unigene_BMK.25259_1025498 | 5408.928 | 3101.544 | 0.000 | -0.802 | down | MIR482 |
| Group2_Unigene_BMK.63506_1315063 | 644.437 | 188.322 | 0.000 | -1.775 | down |  |
|  | **S04** | **S05** |  |  |  |  |
| Group1_Unigene_BMK.45675_802511 | 3.843 | 47.648 | 0.006 | 3.632 | up |  |

| Table S11. Differentially expressed miRNAs of lipid metabolism during *C.* *oleifera* seed natural drying | | | | | | |
| --- | --- | --- | --- | --- | --- | --- |
| **Pre**-**miRNA** | **S06** | **S07** | **FDR** | **log2FC** | **regulated** | **miRNA family** |
| Group1_Unigene_BMK.23434_588836 | 49.935 | 223.486 | 0.000 | 2.162 | up | MIR5067 |
| Group2_Unigene_BMK.34335_1093229 | 49.935 | 223.486 | 0.000 | 2.162 | up | MIR5067 |
| Group2_Unigene_BMK.38504_1137258 | 118.266 | 279.358 | 0.001 | 1.240 | up |  |
| Group1_Unigene_BMK.37987_703484 | 1708.288 | 3128.806 | 0.000 | 0.873 | up | MIR2118 |
| Group2_Unigene_BMK.25259_1025465 | 1708.288 | 3128.806 | 0.000 | 0.873 | up | MIR482 |
| Group2_Unigene_BMK.25259_1025498 | 1708.288 | 3128.806 | 0.000 | 0.873 | up | MIR482 |
| Group2_Unigene_BMK.63506_1315063 | 252.301 | 547.541 | 0.000 | 1.118 | up |  |
|  | **S07** | **S08** |  |  |  |  |
| Group1_Unigene_BMK.23434_588836 | 223.486 | 84.351 | 0.003 | -1.406 | down | MIR5067 |
| Group2_Unigene_BMK.34335_1093229 | 223.486 | 84.351 | 0.003 | -1.406 | down | MIR5067 |
| Group2_Unigene_BMK.38504_1137258 | 279.358 | 134.961 | 0.006 | -1.050 | down |  |
| Group1_Unigene_BMK.37987_703484 | 3128.806 | 2440.547 | 0.000 | -0.358 | down | MIR2118 |
| Group2_Unigene_BMK.25259_1025465 | 3128.806 | 2440.547 | 0.000 | -0.358 | down | MIR482 |
| Group2_Unigene_BMK.25259_1025498 | 3128.806 | 2440.547 | 0.000 | -0.358 | down | MIR482 |
| Group2_Unigene_BMK.63506_1315063 | 547.541 | 191.195 | 0.000 | -1.518 | down |  |
|  | **S09** | **S10** |  |  |  |  |
| CL19455Contig1_54014 | 164.648 | 71.954 | 0.009 | -1.194 | down |  |
| Group1_Unigene_BMK.37987_703484 | 2140.425 | 1845.094 | 0.008 | -0.214 | down | MIR2118 |
| Group2_Unigene_BMK.25259_1025465 | 2140.425 | 1845.094 | 0.008 | -0.214 | down | MIR482 |
| Group2_Unigene_BMK.25259_1025498 | 2140.425 | 1845.094 | 0.008 | -0.214 | down | MIR482 |
| Group2_Unigene_BMK.63506_1315063 | 329.296 | 102.791 | 0.000 | -1.680 | down |  |

| Table S12. Differentially expressed miRNAs of lipid metabolism between two camellia species during seed natural drying | | | | | | |
| --- | --- | --- | --- | --- | --- | --- |
| **Pre**-**miRNA** | **S06** | **S01** | **FDR** | **log2FC** | **regulated** | **miRNA family** |
| Group2_Unigene_BMK.38504_1137263 | 5.256 | 64.830 | 0.003 | 3.625 | up |  |
| CL9644Contig1_380257 | 52.563 | 280.929 | 0.000 | 2.418 | up |  |
| Group1_Unigene_BMK.23434_588836 | 49.935 | 216.099 | 0.000 | 2.114 | up | MIR5067 |
| Group2_Unigene_BMK.34335_1093229 | 49.935 | 216.099 | 0.000 | 2.114 | up |  |
| Group2_Unigene_BMK.9543_1378570 | 44.678 | 162.075 | 0.001 | 1.859 | up |  |
| Group2_Unigene_BMK.38504_1137258 | 118.266 | 378.174 | 0.000 | 1.677 | up |  |
| CL19777Contig1_314088 | 91.985 | 248.514 | 0.001 | 1.434 | up |  |
| CL19455Contig1_54014 | 105.125 | 280.929 | 0.000 | 1.418 | up |  |
| Group1_Unigene_BMK.37987_703484 | 1708.288 | 3263.101 | 0.000 | 0.934 | up | MIR2118 |
| Group2_Unigene_BMK.25259_1025465 | 1708.288 | 3263.101 | 0.000 | 0.934 | up | MIR482 |
| Group2_Unigene_BMK.25259_1025498 | 1708.288 | 3263.101 | 0.000 | 0.934 | up | MIR482 |
| Group2_Unigene_BMK.63506_1315063 | 252.301 | 626.688 | 0.000 | 1.313 | up |  |
|  | **S07** | **S02** |  |  |  |  |
| Group1_Unigene_BMK.37987_703484 | 3128.806 | 4857.301 | 0.000 | 0.635 | up | MIR2118 |
| Group2_Unigene_BMK.25259_1025465 | 3128.806 | 4857.301 | 0.000 | 0.635 | up | MIR482 |
| Group2_Unigene_BMK.25259_1025498 | 3128.806 | 4857.301 | 0.000 | 0.635 | up | MIR482 |
|  | **S08** | **S03** |  |  |  |  |
| Group1_Unigene_BMK.23434_588836 | 84.351 | 238.949 | 0.001 | 1.502 | up | MIR5067 |
| Group2_Unigene_BMK.34335_1093229 | 84.351 | 238.949 | 0.001 | 1.502 | up |  |
| Group1_Unigene_BMK.37987_703484 | 2440.547 | 5408.928 | 0.000 | 1.148 | up | MIR2118 |
| Group2_Unigene_BMK.25259_1025465 | 2440.547 | 5408.928 | 0.000 | 1.148 | up | MIR482 |
| Group2_Unigene_BMK.25259_1025498 | 2440.547 | 5408.928 | 0.000 | 1.148 | up | MIR482 |
| Group2_Unigene_BMK.63506_1315063 | 191.195 | 644.437 | 0.000 | 1.753 | up |  |
|  | **S09** | **S04** |  |  |  |  |
| Group1_Unigene_BMK.37987_703484 | 2140.425 | 3101.544 | 0.000 | 0.535 | up | MIR2118 |
| Group2_Unigene_BMK.25259_1025465 | 2140.425 | 3101.544 | 0.000 | 0.535 | up | MIR482 |
| Group2_Unigene_BMK.25259_1025498 | 2140.425 | 3101.544 | 0.000 | 0.535 | up | MIR482 |
| Group2_Unigene_BMK.63506_1315063 | 329.296 | 188.322 | 0.003 | -0.806 | down |  |
|  | **S10** | **S05** |  |  |  |  |
| Group1_Unigene_BMK.37987_703484 | 1845.094 | 2868.415 | 0.000 | 0.637 | up | MIR2118 |
| Group2_Unigene_BMK.25259_1025465 | 1845.094 | 2868.415 | 0.000 | 0.637 | up | MIR482 |
| Group2_Unigene_BMK.25259_1025498 | 1845.094 | 2868.415 | 0.000 | 0.637 | up | MIR482 |
| Group2_Unigene_BMK.63506_1315063 | 102.791 | 252.535 | 0.000 | 1.297 | up |  |
